# Progranulin AAV gene therapy for frontotemporal dementia: translational studies and phase 1/2 trial interim results

**DOI:** 10.1038/s41591-024-02973-0

**Published:** 2024-05-14

**Authors:** Jeffrey Sevigny, Olga Uspenskaya, Laura Dean Heckman, Li Chin Wong, Daniel A. Hatch, Ambika Tewari, Rik Vandenberghe, David J. Irwin, Dario Saracino, Isabelle Le Ber, Rebekah Ahmed, Jonathan D. Rohrer, Adam L. Boxer, Sebastian Boland, Patricia Sheehan, Alissa Brandes, Suzanne R. Burstein, Benjamin M. Shykind, Sitharthan Kamalakaran, Carter W. Daniels, E. David Litwack, Erin Mahoney, Jenny Velaga, Ilan McNamara, Patricia Sondergaard, Syed A. Sajjad, Yvonne M. Kobayashi, Asa Abeliovich, Franz Hefti

**Affiliations:** 1 Prevail Therapeutics, a wholly owned subsidiary of Eli Lilly and Company, New York, NY USA; 2 Neurology Service, University Hospitals Leuven, Leuven, Belgium and Laboratory for Cognitive Neurology, Department of Neurosciences, Leuven Brain Institute, KU Leuven, Leuven, Belgium; 3 Department of Neurology, Penn Frontotemporal Degeneration Center, University of Pennsylvania, Philadelphia, PA USA; 4 Sorbonne Université, Paris Brain Institute - Institut du Cerveau, ICM, Inserm, CNRS UMR 7225 APHP - Hôpital Pitié-Salpêtrière, Paris, France; 5 Department of Neurology, Royal Prince Alfred Hospital, Sydney, NSW Australia; 6 Department of Neurodegenerative Disease, Dementia Research Center, UCL Queen Square Institute of Neurology, London, UK; 7 Department of Neurology, Memory and Aging Center, University of California, San Francisco, San Francisco, CA USA

**Keywords:** Neurodegenerative diseases, Target validation

## Abstract

GRN mutations cause progranulin haploinsufficiency, which eventually leads to frontotemporal dementia (FTD-GRN). PR006 is an investigational gene therapy delivering the granulin gene (*GRN*) using an adeno-associated virus serotype 9 (AAV9) vector. In non-clinical studies, PR006 transduced neurons derived from induced pluripotent stem cells of patients with FTD-GRN, resulted in progranulin expression and improvement of lipofuscin, lysosomal and neuroinflammation pathologies in Grn-knockout mice, and was well tolerated except for minimal, asymptomatic dorsal root ganglionopathy in non-human primates. We initiated a first-in-human phase 1/2 open-label trial. Here we report results of a pre-specified interim analysis triggered with the last treated patient of the low-dose cohort (*n* = 6) reaching the 12-month follow-up timepoint. We also include preliminary data from the mid-dose cohort (*n* = 7). Primary endpoints were safety, immunogenicity and change in progranulin levels in cerebrospinal fluid (CSF) and blood. Secondary endpoints were Clinical Dementia Rating (CDR) plus National Alzheimer’s Disease Coordinating Center (NACC) Frontotemporal Lobar Degeneration (FTLD) rating scale and levels of neurofilament light chain (NfL). One-time administration of PR006 into the cisterna magna was generally safe and well tolerated. All patients developed treatment-emergent anti-AAV9 antibodies in the CSF, but none developed anti-progranulin antibodies. CSF pleocytosis was the most common PR006-related adverse event. Twelve serious adverse events occurred, mostly unrelated to PR006. Deep vein thrombosis developed in three patients. There was one death (unrelated) occurring 18 months after treatment. CSF progranulin increased after PR006 treatment in all patients; blood progranulin increased in most patients but only transiently. NfL levels transiently increased after PR006 treatment, likely reflecting dorsal root ganglia toxicity. Progression rates, based on the CDR scale, were within the broad ranges reported for patients with FTD. These data provide preliminary insights into the safety and bioactivity of PR006. Longer follow-up and additional studies are needed to confirm the safety and potential efficacy of PR006. ClinicalTrials.gov identifier: NCT04408625.

## Abstract (web summary)

Interim results from the low-dose cohort of the PROCLAIM trial show that AAV-based delivery of the progranulin gene in patients with frontotemporal dementia was safe and was associated with increased CSF progranulin.

## Main

Frontotemporal dementia (FTD) comprises a heterogenous group of clinical syndromes characterized by progressive behavioral, executive, linguistic and motor dysfunction^1,2^. Up to 40% of FTD cases are familial, and approximately one-third of those cases are caused by mutations in one of three genes: *GRN*, coding for progranulin; *MAPT*, coding for tau; and *C9orf72* (chromosome 9 open reading frame 72). *GRN* mutations account for 5–10% of all patients with FTD and approximately 22% of familial FTD cases. Patients with FTD-GRN carry a single mutation in the *GRN* gene, which encodes the progranulin protein, resulting in haploinsufficiency and an approximately 50% reduction in progranulin levels^3^. *GRN* mutations are highly penetrant, and carriers have close to 100% risk of developing FTD^4–7^.

Progranulin is a secreted glycoprotein broadly expressed in the central nervous system (CNS) and periphery in a variety of cells. It is implicated in several physiological functions and roles, including as an activator of lysosome function, an anti-inflammatory and a growth factor^8,9^. The mechanisms by which progranulin deficiency results in FTD are not fully elucidated but likely relate to CNS lysosome dysfunction and inflammation. Progranulin regulates the maturation and processing of key lysosomal enzymes, such as cathepsin D, as demonstrated in mice and in cells derived from patients with FTD-GRN^10–12^. Progranulin deficiency leads to age-dependent reduction of lysosomal protein degradation and recycling in animal models of progranulin deficiency and in patient-derived cells^11,13^ and also leads to neuroinflammation, which is strongly implicated in FTD patient pathology^14,15^. Complete absence of progranulin in mice and humans results in accumulation of lipofuscin that is caused by incomplete lysosomal degradation processes^16,17^.

Given the known functions of progranulin and the adverse consequences of progranulin insufficiency, we and others hypothesized that increasing its level in the brain would attenuate the lysosomal dysfunction and attendant pathology and confer clinical benefit in patients with FTD-GRN. Various therapeutic modalities have been proposed, including gene replacement, gene editing, translational regulation of gene expression and protein replacement therapies^18^. In addition to our gene therapy program, clinical-stage programs include an anti-sortilin monoclonal antibody that inhibits the degradation of progranulin^19^ and a brain-penetrant progranulin biologic^20^. We are pursuing a progranulin gene therapy program because it directly corrects the underlying genetic deficit of FTD-GRN, generates progranulin within the cells rather than delivering it to extracellular compartments, requires a single therapeutic intervention only and is based on an established viral vector technology. PR006 is an adeno-associated virus serotype 9 (AAV9)-GRN vector construct designed to deliver functional copies of the *GRN* gene to neurons and other cells in the brain after a single suboccipital injection into the cisterna magna. Here we report the results from Investigational New Drug-enabling non-clinical efficacy and toxicology studies and from an ongoing open-label phase 1/2 trial in patients with FTD-GRN based on a pre-specified interim analysis.

## Results

**Extended Data Fig. 1:**
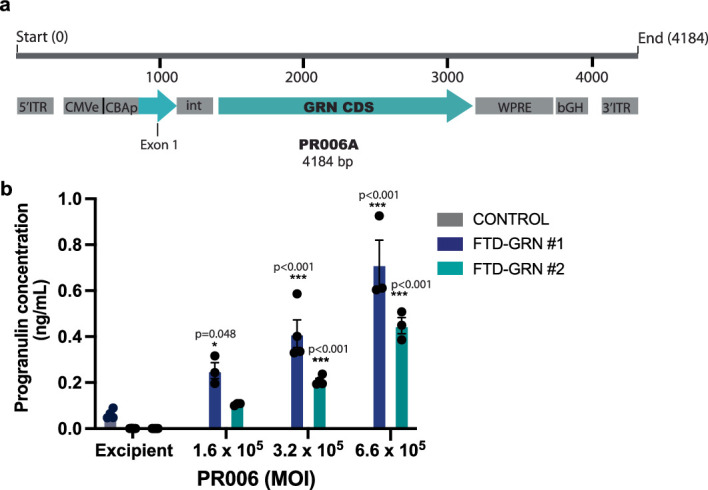
PR006 transduces FTD-GRN mutation carrier neuronal cultures in a dose-dependent manner. (**a**) PR006 consists of a non-replicating recombinant AAV of 4184 nucleotides which includes an expression cassette containing the codon optimized coding sequence of human Progranulin GRN. The viral particle (AAV capsid) is comprised of three capsid proteins, VP1, VP2, and VP3, with the theoretical molecular weights of approximately 87, 73, and 62 kDa. PR006 encapsidates the modified viral vector, including the GRN expression cassette. The vector contains flanking ITRs on each side of the expression cassette, the Cytomegalovirus enhancer (CMVe) and Chicken Beta Actin promoter (CBAp) to constitutively express the codon optimized coding sequence of human GRN. The 3′ region also contains a Woodchuck Hepatitis Virus (WHP) Posttranscriptional Regulatory Element (WPRE) element followed by a bovine growth hormone polyadenylation (bGH polyA) tail. Further information is provided in US Patent 10689625, which covers composition claims for PR006 vector and rAAV. (**b**) Neural stem cells (NSCs) were seeded at an equal density and differentiated into neurons. On day 7, neurons were transduced with excipient or the indicated amounts of PR006 for 72 hours. Secreted progranulin expression was measured from the cell media by ELISA and normalized to volume (n=3–4; mean ± SEM). Black dashed line represents endogenous levels of secreted progranulin from Control neurons (excipient-treated). Secreted progranulin was not detectable in excipient-treated FTD-GRN neurons. Statistics were determined using ANOVA followed by Tukey HSD and statistical comparison of each condition to excipient-treated Control neurons is indicated on the graph. MOI, multiplicity of infection.

PR006 is a non-replicating recombinant adeno-associated virus serotype 9 (rAAV9) containing a *GRN* expression cassette consisting of a cytomegalovirus immediate early enhancer (CMVe), a chicken β-actin (CBA) promoter and a GRN cDNA engineered for optimized human codon usage (rAAV9.CBA.GRN). Detailed information is provided in Extended Data Fig. 1.

### Non-clinical efficacy studies

To establish that PR006 can effectively transduce human cells, we tested it in two independent induced pluripotent stem cell (iPSC) lines derived from patients with FTD-GRN carrying heterozygous *GRN* mutations and differentiated into neurons. PR006 treatment resulted in robust transduction and dose-dependent expression of secreted progranulin in both cell lines (Extended Data Fig. 1).

**Fig. 1:**
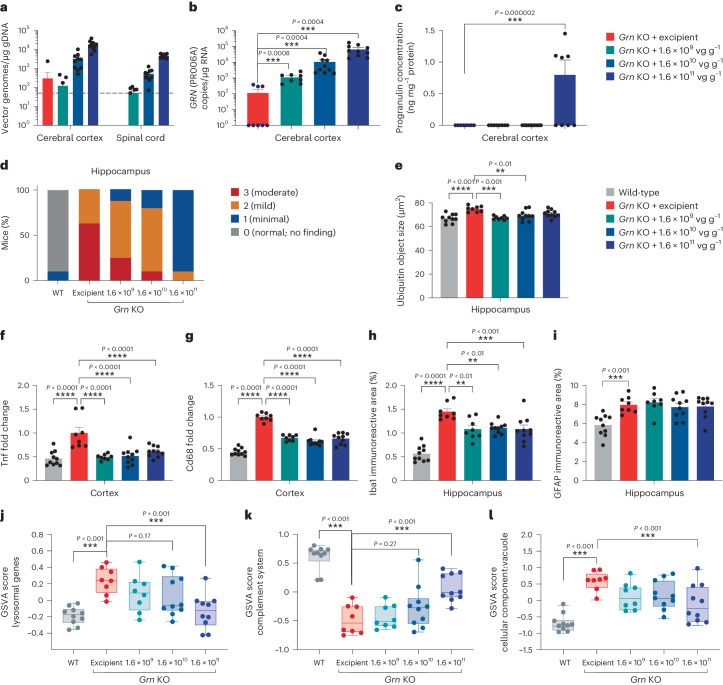
ICV delivery of PR006 in *Grn-*KO mice transduces cells throughout the CNS, increasing progranulin expression and reducing known pathologies. Four-month-old *Grn*-KO mice were dosed with PR006 or excipient by ICV administration (*n* = 10, four males and six females per group in all assays (**a**–**l**)). Three months after dosing, animals were euthanized. **a**, Presence of vector genome per microgram of gDNA on a log scale. Dashed line (50 vg μg^−1^ gDNA) represents the lower limit of detection. **b**, GRN RNA expression was assessed by qRT–PCR in the cerebral cortex. The number of GRN copies (specific to our codon-optimized PR006 sequence) was normalized to 1 µg of total RNA and is shown on a log scale. **c**, Progranulin protein levels were measured in the cortex and normalized to total protein concentration. **d**, Lipofuscin accumulation was semi-quantitatively scored in H&E-stained sections in the hippocampus. **e**, Immunohistochemical (IHC) analysis of ubiquitin was quantified in the hippocampus. The size of above-threshold immunoreactive objects was measured. **f**,**g**, mRNA levels of Tnf (**f**) and Cd68 (**g**) were measured by qRT–PCR in the cortex. **h**,**i**, IHC analysis of Iba1 (**h**) and GFAP (**i**) was quantified in fixed brain sections in the hippocampus. The percent of the area of interest (immunoreactive area) is shown (all data in **a**–**i** represented as mean ± s.e.m.). **j**–**l**, RNA sequencing in cerebral cortex samples is shown as the GSVA activity scores for curated gene sets Lysosome (**j**), Complement System (Hallmark pathway) (**k**) and Cellular Component: Vacuole (GO:0005773) (**l**) (data shown as box plots defined by the minimum, 1st quartile, 2nd quartile (median), 3rd quartile and the maximum). Statistical analysis was conducted using ANOVA followed by Dunnett’s test to compare to the excipient-treated *Grn*-KO mouse group, which kept the family-wise type I error rate at 0.05 rather than 0.15 as implied by the number of comparisons. Exact *P* values are reported in the figure where appropriate, accompanied by **P < 0.01, ***P < 0.001 and ****P < 0.0001. In **d**, excipient versus WT (*P* < 0.0001), versus 1.6 × 10^10^ (*P* = 0.0097) and versus 1.6 × 10^11^ (*P* < 0.0001) were significantly different.

Efficacy of PR006 treatment was established in *Grn-*knockout (KO) mice. Although mice heterozygous for the *Grn* mutation (−/+) more accurately reflect the FTD-GRN patient genotype, they display few of the associated phenotypes^21–23^. In contrast, *Grn*-KO mice (−/−) show lysosomal alterations, neuronal lipofuscin accumulation, ubiquitin accumulation, microgliosis and neuroinflammation, reflecting key pathological features of FTD-GRN^15,22,24–28^. PR006 was delivered via intracerebroventricular (ICV) administration to adult mice, and age-matched *Grn*^+/+^ mice served as controls. At 3 months after dosing, PR006-treated mice showed dose-dependent transduction of vector genomes, human GRN mRNA expression in the brain and human progranulin levels in the CNS (Fig. 1a–c).

**Extended Data Fig. 2:**
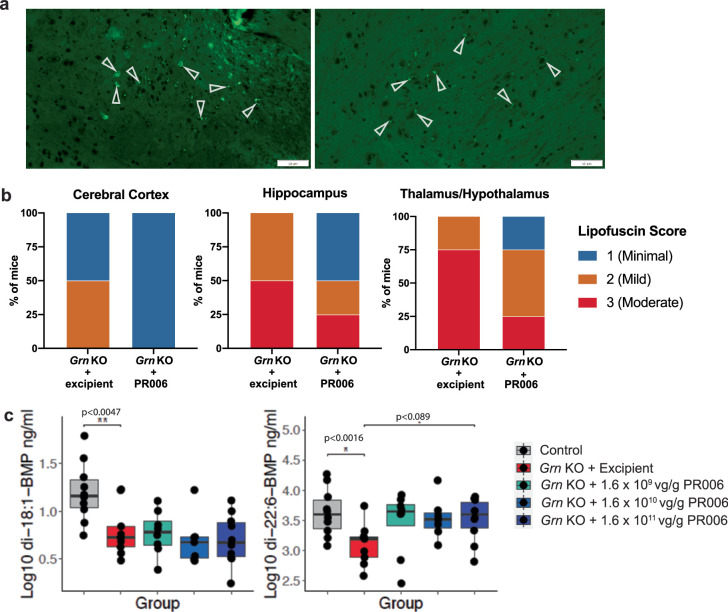
Reduced lysosomal and neuropathology defects in an aged FTD-GRN mouse model following PR006 treatment. Tissue samples were collected from 18-month-old *Grn-*KO mice 2 months after receiving ICV excipient (n=4, 2 males and 2 females) or 5.8 x 10^10^ vg (1.5 x 10^11^ vg/g brain) PR006 (n=5, 3 males and 2 females). (**a**) Representative lipofuscin images from 3 independent studies of the thalamus/hypothalamus region of brain sections (Left = *Grn* KO + Excipient; Right = *Grn* KO + PR006). White arrowheads indicate examples of lipofuscin accumulation. (**b**) A summary of lipofuscin severity scores in the cerebral cortex, hippocampus, and thalamus/hypothalamus of H&E-stained slides from brain sections that were evaluated for autofluorescent lipofuscin granules. Lipofuscin accumulation was semi-quantitatively scored by a blinded board-certified pathologist. (**c**) Urine samples were collected from *Grn-*KO mice 14 weeks after receiving ICV excipient or PR006 (n=9–10 per treatment group, 4–5 males and 4–5 females), while control (wild-type) urine (age, gender and strain matched) were purchased from BioIVT. BMPs were extracted by liquid-liquid extraction from all samples at the same time. BMP concentrations were measured using a SCIEX X500 LC-MS/MS system. Standard curves for di-22:6-BMP and di-18:1-BMP were prepared from corresponding authentic reference standards. Data show as boxplots defined by the minimum, 1^st^ quartile, 2^nd^ quartile (median), 3^rd^ quartile, and the maximum. Statistical analyses were performed using a Dunnett’s Test (a two-sided post-hoc test). Exact 0.05 < *p* < 0.0001 are reported in the figure where appropriate, accompanied by * = *p* < 0.05, ** = *p* < 0.01, *** = *p*< 0.001, ****p < 0.0001. In (b), excipient vs. PR006 was significant in Cerebral Cortex (*p* = 0.005), Hippocampus (*p* < 0.0001), and the Thalamus/Hypothalamus (*p* = 0.038).

Adult *Grn*-KO mice displayed neuronal lipofuscin accumulation in the brain in line with previous reports^24,25,28,29^. PR006 treatment led to a dose-dependent reduction in the severity scores of lipofuscin accumulation (Fig. 1d). Aged *Grn*-KO mice exhibited substantial lipofuscinosis, which was again reduced by PR006 treatment (Extended Data Fig. 2a,b). Ubiquitin-positive inclusions, a defining pathological feature of patients with FTD-GRN, also accumulate in the *Grn*-KO mouse model in an age-dependent manner^5,6,24,25,28^. Compared to wild-type (WT) controls, *Grn*-KO mice exhibited increased ubiquitin immunoreactivity throughout the brain, which was reduced to near WT levels by PR006 at the mid and low doses (Fig. 1e). Chronic CNS inflammation is a pathological feature in the brains of patients with FTD-GRN that is recapitulated in *Grn*-KO mice^15,28,30^. PR006 treatment decreased gene expression of the pro-inflammatory cytokine *Tnf* (TNFα) and *Cd68* (CD68), a marker of microglia (Fig. 1f,g). PR006 treatment also reduced levels of Iba1, a marker of microgliosis, and glial fibrillary acid protein (GFAP), a marker of astrocytosis (Fig. 1h,i).

In addition to the targeted gene expression analysis, we performed RNA sequencing and used gene set variation analysis (GSVA)^31^ to assess gene expression pathways altered in excipient-treated *Grn*-KO mice compared to age-matched WT controls. Previous studies identified significant changes in a subset of the Gene Ontology (GO) term (GO:0005773) ‘Lysosomal Genes’ set (a subset of 25 lysosomal-related genes shown to be dysregulated in *Grn*-KO mice described by Evers et al.^13^); the ‘Complement’ gene set from the gene set enrichment analysis Hallmark database (containing genes encoding components of the complement system of the innate immune system); and the ‘Vacuole’ genes (contains four genes reported to be dysregulated in *Grn*-KO mice described by Lui et al.^15^). Treatment with PR006 dose-dependently reversed the gene set deficiencies observed in the *Grn*-KO mice (Fig. 1j–l).

Bis(monoacylglycero)phosphate (BMP) is a glycerophospholipid enriched in the endolysosomal compartment of the cell and is often dysregulated in lysosomal storage diseases^32^. Two recent studies reported that *Grn*-KO mice exhibit low levels of BMP species in various tissues and urine^20,33^, prompting us to explore the utility of BMP as a translational biomarker in our PR006 program. Two prevalent BMP species, di-18:1-BMP and di-22:6-BMP, were measured in the urine of *Grn-*KO mice treated with PR006. We detected a robust decrease for both BMP species in *Grn*-KO mice, and treatment with PR006 restored di-22:6-BMP to levels similar to that of WT controls (Extended Data Fig. 2c). These results suggest that PR006 treatment restores lysosomal lipid metabolism, including BMP homeostasis in *Grn-*KO mice and, potentially, in patients with FTD-GRN.

### Non-clinical safety studies

**Extended Data Fig. 3:**
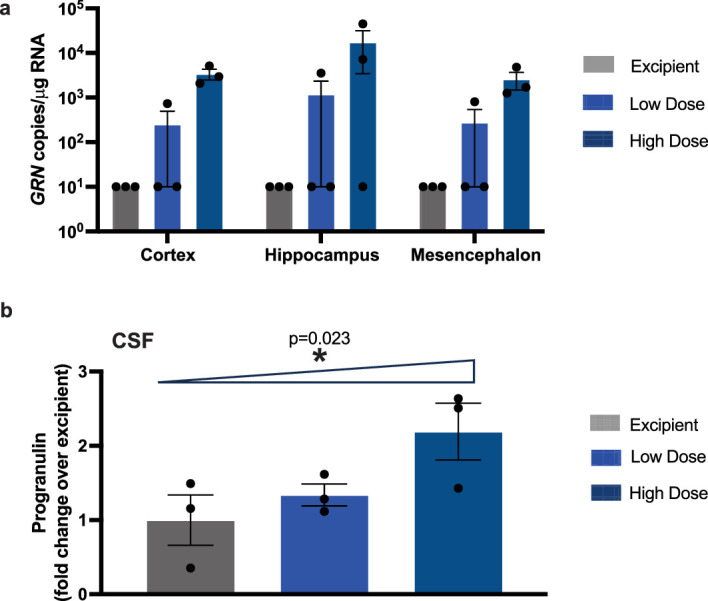
Expression of PR006 transgene (GRN) in non-human primates 6 months post dosing. PR006 was administered once via ICM injection in cynomolgus monkeys (n=3 per treatment group, 2 males and 1 female). Animals were sacrificed at study Day 183. The study evaluated 2 dose levels: a low dose of 2.9 × 10^11^ vg and a high dose of 2.9 × 10^12^ vg; with a brain weight estimate of 74 g in a cynomolgus monkey, the NHP species used in this study, this translates to approximately 3.9 × 10^9^ vg/g brain and 3.9 × 10^10^ vg/g brain. The study also included a control arm in which animals received 1.2 mL of excipient only. (**a**) Tissue samples were collected for qPCR analysis. *GRN* expression levels were determined in NHP cortex, hippocampus and ventral mesencephalon collected on Day 183 using RT-qPCR with primers designed for the PR006 human transgene. Data is presented as mean ± SEM. (**b**) Progranulin levels were measured in CSF samples from NHPs treated with excipient, low dose, or high dose of PR006 collected at Day 183. Progranulin levels were measured using a Simple Western (Jess) analysis. Data is presented as mean ± SEM; P-value: *p = .023, by one-way dose dependence response analysis using William’s trend test.

The toxicology of PR006 was evaluated in cynomolgus macaques, a non-human primate (NHP) species, under Good Laboratory Practice (GLP) conditions. PR006 was administered via intracisterna magna (ICM) injection, the clinical route of administration. Two dose levels were tested, 3.9 × 10^9^ vector genomes per gram (vg g^−1^) of brain and 3.9 × 10^10^ vg g^−1^ of brain, the highest technically feasible dose and 2.4× higher than the initial clinical dose. All animals survived until the scheduled necropsy up to 6 months after dosing. There were no adverse PR006-related clinical observations, body weight changes, ophthalmic observations or physical or neurological examination findings, and gross macroscopic examination at necropsy showed no drug-related abnormalities in any of the cohorts. In dorsal root ganglia (DRG), minimally increased cellularity, related to enhanced numbers of satellite glial cells, was observed in a few PR006-treated animals. This finding is consistent with a glial reaction to neuronal injury and is a known consequence of gene therapy test articles for CNS targets^34^. Postmortem analysis confirmed that PR006 treatment resulted in dose-dependent expression of *GRN* at the mRNA level in the brain and elevated levels of progranulin protein in the cerebrospinal fluid (CSF), also indicating that CSF progranulin levels reflect synthesis of this protein in the brain (Extended Data Fig. 3).

### Phase 1/2 clinical trial: design, patient disposition and dose selection

**Fig. 2:**
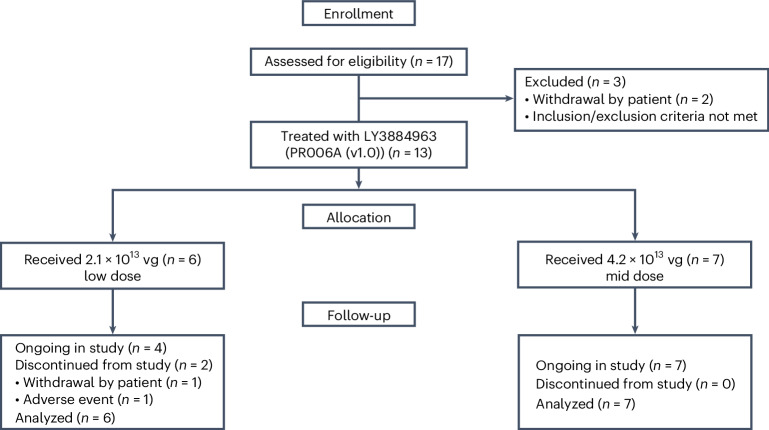
Patient disposition. Enrolled patients were 55–75 years old, had a confirmed pathogenic GRN mutation and had symptomatic FTD-GRN with CDR plus NACC FTLD sum of boxes score between 1 and 15. One patient was enrolled and remained in active screening at the time of submission for publication.

PR006 is being assessed in humans in a phase 1/2 clinical trial in patients with FTD-GRN. This multi-center, open-label, ascending-dose, first-in-human study was designed to evaluate safety, tolerability, immunogenicity, effects on progranulin levels and effects on other biomarkers. Primary endpoints are safety and progranulin levels; secondary endpoints are Clinical Dementia Rating (CDR) plus National Alzheimer’s Disease Coordinating Center (NACC) Frontotemporal Lobar Degeneration (FTLD) rating scale and levels of neurofilament light chain (NfL), and multiple exploratory endpoints include BMP levels. Demographic data and baseline characteristics of the patient population are listed in Table 1. Patient disposition is shown in Fig. 2. The data reported here were obtained as part of a pre-specified interim analysis when all patients in the low-dose cohort completed the 12-month follow-up period. We additionallly included, as part of this interim analysis, the available 6-month data from the mid-dose cohort.

**Table 1:** Demographics and baseline characteristics

Baseline characteristic/endpoint by timepoint	Low-dose PR006 *n* = 6^a^	Mid-dose PR006 *n* = 7^b^	Total *n* = 13
Age (years), mean (s.d.)	64.7 (6.92)	61.9 (4.60)	63.2 (5.71)
Sex (males), *n* (%)	3 (50.0%)	4 (57.1%)	7 (53.8)
Number of years diagnosed with FTD, mean (s.d.)	1.1 (0.68)	1.8 (2.45)	1.50 (1.82)
Phenotypic presentation of symptoms, *n* (%)			
bvFTD	2 (33.3%)	6 (85.7%)	8 (61.5%)
PPA	1 (16.7%)	0	1 (7.7%)
Other	0	0	0
Combination	3 (50.0%)	1 (14.3%)	4 (30.8%)
NACC FTLD CDR global score, *n*, mean (s.d.)			
Baseline	6, 1.42 (0.665)	7, 1.57 (0.535)	
NACC FTLD CDR sum of boxes, *n*, mean (s.d.)			
Baseline	6, 8.08 (5.073)	7, 9.42 (3.372)	
CSF PGRN (ng ml^−1^), *n*, mean (s.d.)			
Baseline	6, 2.36 (0.437)	7, 2.29 (0.510)	
Urine BMP, di-18:1, *n*, mean (s.d.)			
Baseline	5, 1.97 (1.698)	6, 3.91 (2.794)	
Urine BMP, di-22:6, *n*, mean (s.d.)			
Baseline	5, 9.13 (4.781)	6, 21.92 (11.238)	

PGRN, progranulin.

^a^Six patients received the low dose: five per protocol and one due to pharmacy dose preparation error.

^b^Seven patients received the mid dose: five per protocol and two reallocated from initially planned high-dose cohort, which was not used, because the mid-dose cohort achieved and exceeded the intended elevations of CSF PGRN levels in the study participants.

We selected 2.1 × 10^13^ vg (1.6 × 10^10^ vg g^−1^ brain) as the starting clinical dose for PR006 as it corresponds to the level at which robust biomarker and pathological benefits were seen in an animal model and maintains a reasonable and feasible safety margin based on the GLP NHP toxicology study. Furthermore, it takes into consideration the severity and absence of treatment options available for patients with FTD-GRN. The mid dose of 4.2 × 10^13^ vg represents a 2× increase over the low dose. A high dose was included in the original study protocol but was not implemented given the biomarker effects observed at the low and mid dose levels. Methods section contains a detailed outline of the dose rationale.

### Primary endpoint: safety, tolerability and immunogenicity

Treatment-emergent adverse events (TEAEs) occurred in all patients (Table 2). CSF pleocytosis, with or without an elevation of CSF protein, occurred in six patients and was the most common PR006-related adverse event. The CSF changes were detected at month 2 per protocol lumbar puncture and generally resolved over the ensuing 3–6 months. In five of the six patients, the CSF pleocytosis was asymptomatic; however, one patient developed decrease in hearing, which was recovering at the time of the interim analysis.

**Table 2:** Summary of safety: TEAEs and immunogenicity

	Low-dose PR006 *N* = 6 *n* (%) [E]	Mid-dose PR006 *N* = 7 *n* (%) [E]	Total *N* = 13 *n* (%) [E]
Any TEAE	6 (100) [58]	7 (100) [42]	13 (100) [100]
Any treatment-related TEAE	2 (33.3) [6]	5 (71.4) [8]	7 (53.8) [14]
Severity of TEAEs			
Mild	0 [23]	0 [23]	0 [46]
Moderate	0 [22]	4 (57.1) [12]	4 (30.8) [34]
Severe	3 (50.0) [9]	2 (28.6) [4]	5 (38.5) [13]
Life-threatening	2 (33.3) [3]	1 (14.3) [3]	3 (23.1) [6]
Death	1 (16.7) [1]	0	1 (7.7) [1]
Severity of treatment-related TEAEs			
Mild	0 [2]	1 (14.3) [2]	1 (7.7) [4]
Moderate	2 (33.3) [4]	2 (28.6) [3]	4 (30.8) [7]
Severe	0	1 (14.3) [2]	1 (7.7) [2]
Life-threatening	0	1 (14.3) [1]	1 (7.7) [1]
Death	0	0	0
Any SAE	3 (50.0) [5]	3 (42.9) [7]	6 (46.2) [12]
Any treatment-related SAE	0	2 (28.6) [4]	2 (15.4) [4]
Any TEAE leading to early discontinuation	1 (16.7) [1]	0	1 (7.7) [1]
Baseline positive anti-AAV9 antibodies in CSF, *n*/*N* (%)	0/6	5/7 (71.4)	5/13 (38.5)
Patients with any treatment-emergent positive anti-AAV9 antibodies in CSF, *n*/*N* (%)	5/5 (100)	6/6 (100)	11/11 (100)
Baseline positive anti-AAV9 antibodies in blood, *n*/*N* (%)	0/5	5/7 (71.4)	5/13 (38.5)
Patients with any treatment-emergent positive anti-AAV9 antibodies in blood, *n*/*N* (%)	6/6 (100)	5/6 (83.3)	11/12 (91.6)
Baseline positive anti-GRN antibodies in CSF, *n*/*N* (%)	0/6	0/7	0/13
Patients with any treatment-emergent positive anti-GRN antibodies in CSF, *n*/*N* (%)	0/5	0/6	0/11
Baseline positive anti-GRN antibodies in blood, *n*/*N* (%)	0/6	0/7	0/13
Patients with any treatment-emergent positive anti-GRN antibodies in blood, *n*/*N* (%)	0/6	0/6	0/12

A TEAE is defined as any event not present before exposure to investigational product or any event already present that worsens in either intensity or frequency after exposure to investigational product. For summarization by severity, a patient is counted once under the most severe event. *n* represents the number of patients at each level of summarization. Patients can have more than one event in each case. Percentages for AEs are based on the number of patients in the Safety Analysis Set within each treatment group and overall. [E] represents the number of events at each level of summarization. A treatment-emergent positive antibody is defined as a post-treatment positive finding when the baseline was negative or at least a four-fold increase in titer from the baseline level when the baseline titer was positive. Percentages for immunogenicity findings are based upon the number of patients with data in the Safety Analysis Set within each treatment group and overall. TEAEs/SAEs are based on the investigator-assigned causality assessment.

In addition, treatment-emergent transaminitis (grade 1), osteoporosis (grade 2) and hypercholesterolemia or hyperlipidemia (grade 1) occurred in one patient (7.7%), and treatment-emergent diabetes (grade 1) and pre-diabetes (grade 1) occurred in two patients (15.4%). The event of transaminitis was non-serious, mild and assessed by the investigator as unrelated to study drug and related to sirolimus. The outcome was reported as recovered. All of these events occurred in the 2.1 ×10^13^ vg dose level.

Twelve serious adverse events (SAEs) occurred in six patients (Extended Data Table 1). Eight of the 12 SAEs were considered by the investigator and sponsor as unrelated. Of the four SAEs considered by the investigator as related, two were assessed by the sponsor as unrelated.

Three patients developed thrombotic events within 2 months of receiving PR006. One patient was diagnosed with deep vein thrombosis (DVT) (grade 2 adverse event (AE), not related) and pulmonary embolism (grade 2 AE, not related) shortly after being hospitalized with perforated diverticulitis (SAE, not related); the second patient developed DVT (SAE, not related) and pulmonary embolism (SAE, not related), which was attributed by the investigator to immobility related to travel; and the third patient was diagnosed with DVT (SAE, related per investigator, not related per sponsor) while in the hospital for a stroke (SAE, not related) due to a carotid artery dissection (SAE, not related). Each patient had multiple risk factors for developing a DVT, including concurrent use of sirolimus and corticosteroids, increased body mass index and immobility. There was no clear association with DVT and increase in plasma progranulin: in one patient, progranulin transiently increased 18× above baseline 1 month after treatment; in another patient, there was no increase over baseline as tested monthly out to month 9; and in a third patient, follow-up progranulin was not available.

There was one non-study drug-related death due to respiratory acidosis, occurring more than 18 months after dosing. No patient discontinued due to drug-related AEs.

PR006 treatment elicited an anti-AAV9 antibody response in most patients (Table 2). Five of the 13 patients had a positive baseline anti-AAV9 titer. By the end of the observation period, all patients who completed a post-treatment titer assessment had a positive titer. No patient had a positive baseline anti-progranulin titer or developed a positive titer during the observation period, indicating that the transgene-derived human progranulin did not elicit an immune response.

### Primary endpoint: efficacy

**Fig. 3:**
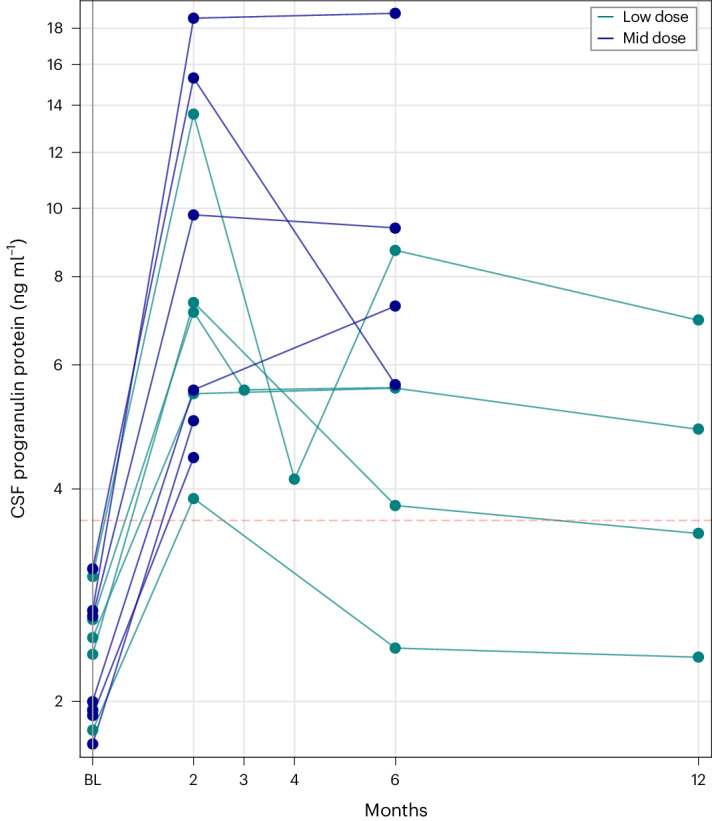
PR006 increases progranulin levels in CSF of FTD-GRN study participants. CSF samples were collected from study participants in the low-dose (2.1 × 10^13^ vg, *n* = 5) and mid-dose (4.2 × 10^13^ vg, *n* = 6) cohorts. Progranulin concentrations (ng ml^−1^) in CSF samples were measured by ELISA and plotted against timepoint of collection (baseline and months 2, 6 and 12; additional timepoints were collected at months 3 and 4 for two study participants). At baseline, progranulin concentrations were all below normal levels, as reported by Goossens et al.^35^ (denoted in the figure by the red line). Upon dosing with PR006, at the month 2 timepoint, levels of progranulin concentration increased by 2.1–6.9× above the respective baseline levels. Among the nine patients with CSF progranulin data at month 6, eight (89%) had progranulin level within or above the normal range, whereas, among the four patients with 12-month data, three (75%) had levels within or above the normal range, as described by Goossens et al.^35^.

Intrathecal treatment with PR006 via a suboccipital injection into the cisterna magna was predicted to increase progranulin in the brain and, if there is sufficient PR006 efflux from the CNS, potentially in certain peripheral organs (for example, liver). We used CSF and blood progranulin levels as a proxy for the amount of cellular transduction in these compartments. All patients had a low baseline CSF progranulin level compared to non-carrier values published by Goossens et al.^35^. After treatment with PR006, CSF progranulin levels were increased in all patients at all timepoints compared to their baseline values (Fig. 3). Two months after treatment with PR006, progranulin increased 2.1–4.5× in the low-dose cohort and 2.7–6.9× in the mid-dose cohort. Among the nine patients with CSF progranulin data at month 6, eight (89%) had progranulin level within or above the normal range, whereas, among the four patients with 12-month data, three (75%) had levels within or above the normal range, as described by Goossens et al.^35^.

**Extended Data Fig. 4:**
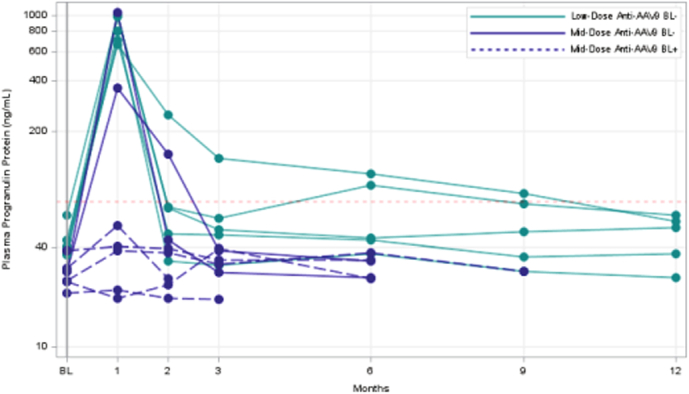
PR006 administration leads to transient elevations in plasma progranulin levels of FTD_GRN study participants without pre-existing anti-AAV9 antibodies. Plasma samples were collected from study participants in the low-dose (2.1 × 10^13^ vg, n=5) and mid-dose 4.2 × 10^13^ vg, n=6) cohorts. Progranulin concentrations (ng/mL) in plasma samples were measured by ELISA and plotted against timepoint of collection (baseline, months 1, 2, 3, 6, 9, and 12). At baseline, progranulin concentrations were all below the cut-off value reported by Swift et al.^37^, denoted in figure by red line). Upon dosing with PR006, at the month 1 timepoint, levels of progranulin concentration increased by 12x to 36x above the respective baseline levels in 8 subjects, which tested negative for anti-AAV9 antibodies at baseline. In the 8 patients the levels generally decreased to baseline within 2–3 months (values not available for 1 subject). Five subjects tested positive for anti-AAV9 antibodies at baseline and showed minimal changes from baseline (0.8x to 1.8x).

Regarding plasma progranulin, all patients had a baseline level below published cutoff levels^36,37^. Plasma levels increased in most patients 1 month after treatment (range from 0.8× to 36× baseline level) and then generally decreased to baseline within 2–3 months. Upon an exploratory post hoc analysis, we found that, among the eight patients with a negative baseline anti-AAV9 antibody titer, plasma progranulin increased 12× to 36× 1 month after treatment and then generally decreased to baseline within 2–3 months (values not available for one patient); among the five patients with a positive baseline anti-AAV9 antibody titer, plasma progranulin changes from baseline were minimal (0.8–1.8×) (Extended Data Fig. 4).

### Secondary endpoints

**Extended Data Fig. 5:**
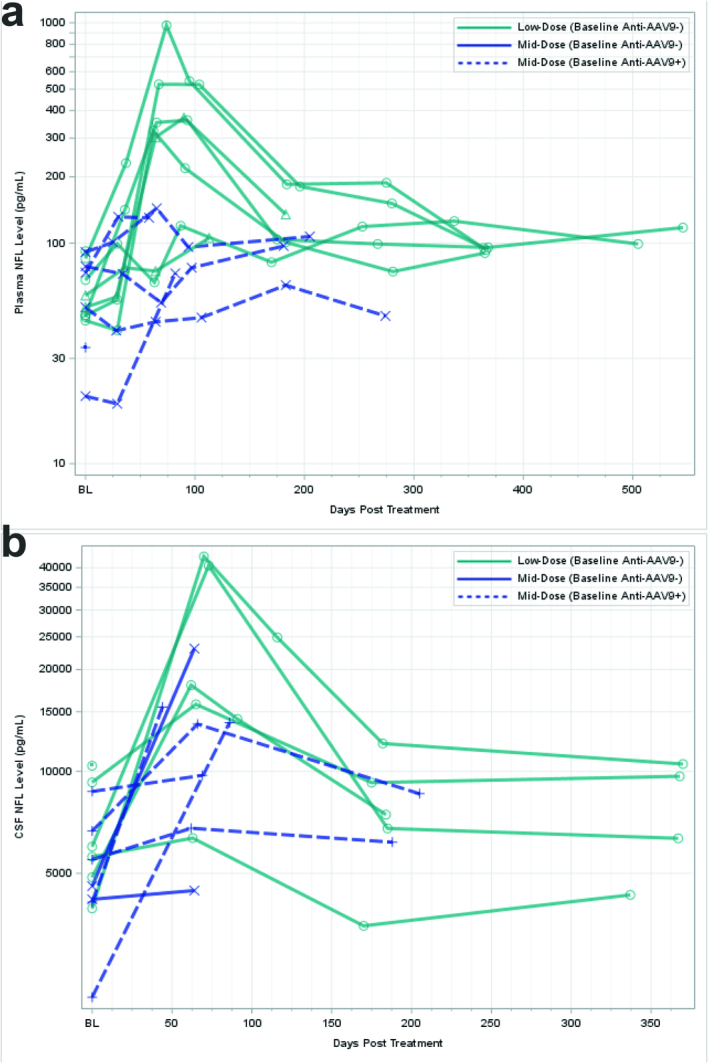
PR006 administration leads to transient elevations in plasma and CSF NfL levels. Plasma and CSF samples were collected from study participants in the low-dose (2.1 × 10^13^ vg, n=5) and mid-dose 4.2 × 10^13^ vg, n=6) cohorts. (**a**) NfL concentrations (pg/mL) in plasma samples were measured using the Quanterix Simoa NfL assay and plotted against timepoint of collection (baseline, months 1, 2, 3, 6, 9, and 12). Upon dosing with PR006, plasma levels increased by up to 11x above the respective baseline levels at 2 months and returned to baseline levels or slight elevations at 9–12 months after dosing. Elevations tended to be lower in subjects who tested positive for anti-AAV9 antibodies at baseline. (**b**) NfL concentrations (pg/mL) in the CSF were measured at baseline, and months 2, 6 and 12. Upon dosing with PR006, CSF levels increased up to 11x at 2 months and returned to baseline levels, reductions from baseline or slight elevations at 6 to 12 months after dosing.

NfL is considered a biomarker for neurodegeneration and acute CNS inflammation^38–40^. In our study, NfL levels transiently increased in both plasma and CSF at both dose levels (Extended Data Fig. 5). NfL increase is thought to be the result of transduction and transgene overexpression in DRG with secondary inflammatory response. None of the patients developed any signs or symptoms of sensory neuron dysfunction. Plasma increases tended to be lower in patients with a baseline blood anti-AAV9 titer, suggesting an attenuating effect of anti-AAV9 antibodies on PR006 transduction of DRG.

Cognitive/behavioral performance was measured using the CDR plus NACC FTLD scale^41,42^ at baseline and then at 6 months and 12 months after PR006 treatment. CDR plus NACC FTLD is a semi-quantitative scoring system assessing eight domains (memory, orientation, judgment and problem solving, community affairs, home and hobbies, personal care, behavior/comportment/personality and language) impaired in FTD. Each domain is rated on a five-point scale, ranging from 0 (normal), 0.5 (questionably or minimally impaired), 1 (mildly impaired), 2 (moderately impaired) to 3 (most severely impaired), with the exception of the ‘personal care’ domain, which is rated on a four-point scale from 0 to 3 without a rating of 0.5. At baseline, the sum of boxes total and global scores were similar in the low-dose and mid-dose cohorts, respectively: 8.08 ± 5.07 and 9.42 ± 3.37 (Table 1). At month 6, the sum of boxes total scores increased compared to baseline in the low-dose and mid-dose cohorts by, respectively, 2.30 ± 2.61 and 3.90 ± 2.68 (mean ± s.d.). At month 12, in the low-dose cohort, the sum of boxes increased by 4.70 ± 3.23. These progression rates are within the broad ranges reported for patients with FTD^41^.

### Exploratory endpoints

**Fig. 4:**
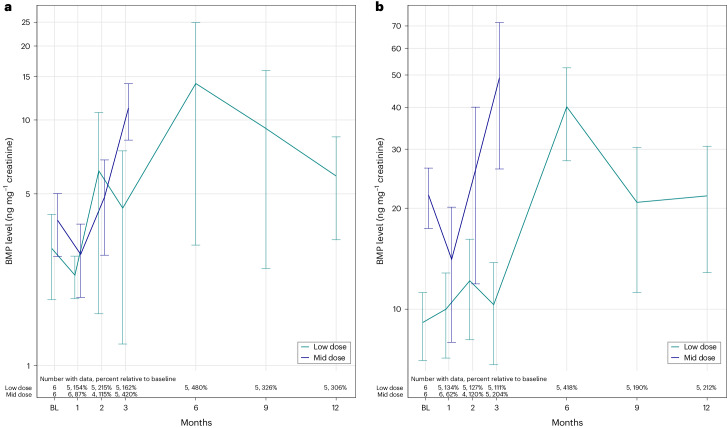
Levels of total di-18:1-BMP and di-22:6-BMP in urine were increased upon PR006 dosing of patients with FTD-GRN. Urine samples were collected from study participants in the low-dose (2.1 × 10^13^ vg, *n* = 6) and mid-dose (4.2 × 10^13^ vg, *n* = 6) cohorts. **a**,**b**, Total di-18:1-BMP (**a**) and di-22:6-BMP (**b**) levels were measured using a mass spectrometry–based assay and were normalized with creatinine concentrations (ng mg^−1^). Mean (±s.e.m.) values were plotted against timepoints of sample collection (baseline and months 1, 2, 3, 6, 9 and 12). Percent of total di-18:1-BMP and di-22:6-BMP levels relative to baseline is shown. For both BMP species, an increase of BMP levels compared to baseline was observed. The increase of BMP was sustained until month 12 for the low-dose cohort and until month 3 for the mid-dose cohort. Data plots of individual patients are provided in Extended Data Fig. 6. The increased BMP levels approached values measured in urine samples obtained from healthy human controls, which were measured as 40.8 ± 8.4 and 50.4 ± 7.7, respectively, for di-18:1-BMP and di-22:6-BMP (mean ± s.e.m., *n* = 24).

BMP was evaluated as a downstream pharmacodynamic readout of PR006 gene therapy efficacy. Within the lysosomes, BMP is required for adequate lipid degradation by increasing catabolic activities of lysosomal enzymes^43^. BMP is released into urine by association with endosome-derived exosomes^20,33,44,45^, and urinary BMP levels represent a potentially useful biomarker of lysosomal dysfunction^32^. Recent findings indicate that urinary BMP levels are altered in LRRK2 mutation carriers with Parkinson’s disease compared to asymptomatic mutation carriers^46–48^, supporting the view that altered urinary BMP levels might be reflective of brain pathology. In both the low-dose and mid-dose cohorts, increases in urine levels of the two prevalent species of BMP, di-18:1-BMP and di-22:6-BMP, were detected after treatment with PR006, and the increases approached values measured in urine samples obtained from healthy human controls (Fig. 4).

## Discussion

In this interim analysis of the open-label phase 1/2 trial in patients with FTD-GRN, treatment with PR006 was generally safe and well tolerated and increased CSF progranulin and urinary levels of BMP, a functional readout potentially indicative of pharmacological efficacy at the lysosomal level. The clinical study was enabled by non-clinical experiments demonstrating that PR006 corrects functional deficits in iPSCs derived from patients with FTD-GRN, is efficacious in a mouse model of FTD-GRN after intraventricular administration and is safe and well tolerated in NHPs after administration into the cisterna magna via suboccipital injection, the clinical route of administration.

The PR006 program was founded on a strong genetic rationale: monoallelic null mutations, or, in rare instances, biallelic hypomorphic mutations in the *GRN* gene, result in haploinsufficiency with an approximately 50% reduction in progranulin levels and a virtually 100% risk of developing FTD typically beginning in the fifth or sixth decade of life^3–6^. In the CNS, progranulin is expressed by neurons and, to a lesser extent, by other cell types and is primarily secreted into the interstitial space whereupon it undergoes sortilin-mediated endocytosis and subsequent trafficking to the lysosomes where it regulates the maturation and processing of lysosomal enzymes^8,49^. In animal models, progranulin deficiency results in reduced lysosomal-mediated protein degradation and recycling and subsequent neuroinflammatory changes^9–14^. In humans, FTD-GRN pathology is characterized by aggregates of ubiquitin and cytoplasmic TDP-43, neuroinflammation and neurodegeneration. The known functions of progranulin and the adverse consequences of progranulin insufficiency support the hypothesis that elevating its levels in the brain would attenuate lysosomal dysfunction, neuropathological sequalae and possibly the clinical decline of patients with FTD-GRN. Given the genetic loss-of-function nature of FTD-GRN, we embarked on a strategy to use an AAV gene transfer approach whereby a viral vector transduces neurons and other cells in the CNS with a copy of the *GRN* gene, wherein the *GRN* transgene remains episomal and constitutively expresses progranulin at sufficient levels to restore normal cellular function.

AAV9 was selected as the capsid for PR006 because of its tropism for neurons, its established safety and tolerability profile reported with other investigational and approved therapies and its proven efficacy in spinal muscular atrophy^50–52^. Because progranulin is primarily a secreted protein, it is thought that only a fraction of cells needs to be transduced to generate sufficient levels of progranulin to cross-correct non-transduced cells. We chose to administer PR006 via suboccipital injection into the cisterna magna because data generated by us and others have demonstrated that this route of administration results in better brain biodistribution than intrathecal lumbar or intravenous approaches^53–56^. Furthermore, AAV-based gene therapies delivered directly into the CSF, which is relatively immune privileged, are less likely to be affected by anti-AAV neutralizing antibodies^54,57–63^. The starting dose chosen for the phase 1/2 study was one predicted to be safe and to double CSF progranulin to achieve normal physiological levels, based on the results from the FTD-GRN mouse model and from NHP toxicology studies. The dose rationale deviates from the conventional rationale used for other drug modalities but is appropriate given that PR006 is a potential one-time and irrevocable therapy.

Treatment with PR006 was generally safe, and the procedure was well tolerated. DVT occurred in three patients, and each had multiple risk factors, including use of sirolimus and corticosteroids, confounding the ability to assign definite cause. A thorough review of the literature did not identify a risk of developing DVT with AAV-based gene therapies. Based on the totality of available data, DVT cases were deemed to be unrelated to the study drug per sponsor causality assessment; however, it cannot be excluded that PR006 caused or contributed to the development of DVTs. Neuroinflammation reflected by pleocytosis remained asymptomatic in most patients and seemed to be attenuated by corticosteroids. The use of sirolimus and rituximab did not have an obvious effect of attenuating the CSF pleocytosis. Therefore, given their potential side effects, including the risk of DVT with sirolimus, both were eventually discontinued in the study. Nevertheless, together with the observed elevations in NfL (discussed below), AAV9-mediated inflammation is an area that warrants further investigation and close monitoring in AAV-based, intrathecally delivered therapies. The treatment generated an immune response against AAV9, but this did not have any apparent deleterious effect on CNS transduction. No immune response against the transgene-derived progranulin was detected.

The clinical study results fulfilled the primary efficacy endpoint expectations: treatment with both low and mid dose levels of PR006 resulted in an increase of CSF progranulin in all patients, and the increase remained within or above the normal range reported in the literature^35^ in all but one patient at the final timepoint in which CSF was collected in this interim analysis. The desired progranulin increase was achieved with the low and mid dose levels, and, therefore, escalation to the high dose was abandoned. It remains unknown whether increasing CSF progranulin in FTD-GRN will confer clinical efficacy and, if so, what level of increase is needed or optimal.

PR006 also elevated progranulin levels in the blood, and there was a strong negative association between the presence of pre-existing blood anti-AAV9 antibodies and blood (but not CSF) progranulin increase. These results suggest that pre-existing anti-AAV9 antibodies neutralized PR006 that migrated from the CNS. Furthermore, among the patients with an increase in blood progranulin, the increase was generally marked but also transient. The transient nature of the increase suggests that the peripheral cells responsible underwent relatively rapid turnover approximately 1–2 months after treatment. We have not identified the cell type/organ responsible for the spike in blood progranulin, and no safety signals that may be associated with cell turnover were observed on routine blood tests (for example, cell counts, chemistries and liver function tests).

Increased blood progranulin has been associated with tumorigenicity^64^. However, direct peripheral intravenous administration of progranulin in vivo has been not reported to be associated with tumorigenesis^65^, and our own in vivo studies using PR006 to overexpress progranulin in mouse models or normal NHPs have not revealed tumorigenic effects. Based on this and the transient nature of the increase observed in certain patients treated with PR006 (Extended Data Fig. 4), there is unlikely to be an increased risk of tumorigenesis or malignancy after PR006 administration. Nevertheless, this is being monitored during the 5-year follow-up period.

CSF and blood NfL were chosen as a secondary endpoint potentially indicative of efficacy. CSF NfL is increased in many neurodegenerative diseases, including FTD, and is thought to reflect ongoing neuronal degeneration^39,40^. NfL is also increased in the setting of acute inflammation (for example, CNS infection) in the absence of CNS dysfunction or neurodegeneration^38,66,67^. We had hypothesized that treatment with PR006 would result in a lowering of CSF NfL; however, all patients developed a transient increase in blood and CSF NfL without concurrent (relative to natural history) cognitive, behavioral or functional worsening. This transient increase likely reflects inflammatory or degenerative changes in DRG, as marginal DRG changes were observed in the NHP study and have been documented by other investigators^68,69^. Patients are closely monitored, including with magnetic resonance imaging (MRI), neurological examination and the Treatment-Induced Neuropathy Scale^70^ and no findings have been observed. Nevertheless, NfL is not a useful marker of efficacy in our gene therapy study at early timepoints.

This study was not designed around clinical outcomes and, furthermore, enrolled patients with relatively advanced dementia who would be less likely to respond to a disease-modifying treatment. Notwithstanding, patients receiving PR006 declined generally similarly to matched patients from natural history studies. A larger study with a longer observation period in less clinically advanced patients with FTD-GRN will be necessary to determine whether PR006 slows clinical progression.

Levels of BMP, a phospholipid critical to lysosomal function, have been reported to be reduced in brain tissue of progranulin-deficient mice and patients with FTD-GRN^20,33^, and urinary levels of BMP may be reflective of lysosomal dysfunction and of brain pathology^47,48^. We observed increases of relevant BMP species in the urine of patients treated with PR006. This finding provides downstream evidence of a pharmacological effect of PR006, but its clinical relevance requires further investigation.

The non-clinical studies and the clinical trial have obvious limitations. The animal model, homozygous *Grn*-KO mice, has a complete loss of progranulin, whereas patients with FTD-GRN have approximately 50% of normal levels of progranulin. It was necessary to use these animals because the heterozygous *Grn*-KO mice do not have a discernable phenotype. The data obtained from the homozygous KO mice may, therefore, have slightly misdirected the dose selection, as evidenced by the elevation of progranulin beyond anticipated levels by our test doses. The clinical study was not blinded and did not include a placebo control group, and the number of treated patients was relatively small. The main study duration was limited to 12 months, whereas the effects of PR006 gene transfer are expected to last for several years. Whether this increase is clinically relevant needs to be tested in a double-blind study, ideally with a less advanced patient population.

In conclusion, treatment with the investigational gene therapy PR006 was efficacious in a human in vitro model and in a transgenic animal model of FTD-GRN and was generally safe and well tolerated in healthy NHPs. These findings appear to translate to patients with FTD-GRN, thus supporting further clinical development of PR006 in this population.

## Methods

All mouse studies were performed by PsychoGenics in accordance with approved procedures by the local Institutional Animal Care and Use Committee (IACUC) and the National Institutes of Health (NIH) Guide for the Care and Use of Laboratory Animals.

The toxicology of PR006 was evaluated in cynomolgus macaques, an NHP species, in a study conducted by Labcorp (formerly Covance) under GLP conditions. Labcorp is fully accredited by the Association for Assessment and Accreditation of Laboratory Animal Care (AAALAC). All procedures in the protocol were in compliance with applicable animal welfare acts and were approved by the local IACUC. The study design was based on the principles of the US Food and Drug Administration Center for Drug Evaluation and Research (CDER)/International Conference on Harmonization (ICH) Harmonized Tripartite Guidelines ICH-M3(R2), Nonclinical Safety Studies for the conduct of Human Clinical Trials and Marketing Authorization for Pharmaceuticals (CDER, January 2010).

The clinical study followed accepted guidelines for inclusion and ethics and was conducted under oversight by regulatory agencies of countries with trial sites and by an independent data monitoring committee. Institutional review board (IRB) approval was obtained from the University of California, San Francisco (UCSF) Medical Central IRB; the Advarra IRB; the University of Pennsylvania IRB; South Central – Oxford A; CEIC de Euskadi; CEIM Hospital Clinic de Barcelona; UZ Leuven – Commissie Medische Ethiek – toetsingscommissie; and the Sydney Local Health District (SLHD) Ethics Review Committee (RPAH Zone). Informed consent was obtained from all participants.

### PR006 design and production

PR006 (LY3884963) is an rAAV9.CBA.GRN, a non-replicating rAAV9 that contains a GRN expression cassette with a CMVe and CBA promoter with a GRN cDNA engineered for optimized human codon usage. PR006 consists of a non-replicating rAAV of 4,184 nucleotides. The AAV capsid comprises three capsid proteins, VP1, VP2 and VP3, with the theoretical molecular weights of approximately 87 kDa, 73 kDa and 62 kDa. PR006 encapsidates the modified viral vector, including the *GRN* expression cassette. The vector contains flanking inverted terminal repeats (ITRs) on each side of the expression cassette, the CMVe and the CBA promoter (CBAp) to constitutively express the codon-optimized coding sequence of human *GRN*. The 3′ region also contains a woodchuck hepatitis virus post-transcriptional regulatory element element followed by a bovine growth hormone polyadenylation (bGH poly(A)) tail. PR006 was produced by triple plasmid transfection of human HEK293 Working Cell Bank with the vector plasmid, an AAV9 helper plasmid containing the AAV rep and cap genes and a helper adenovirus plasmid. Subsequent downstream processing involved disposable, closed bioprocessing circuits (filtration, tangential flow filtration and column chromatography). After vector purification, PR006 drug substance was formulated in 20 mM Tris (pH 8.0), 1 mM MgCl_2_ and 200 mM NaCl containing 0.001% (w/v) poloxamer 188 and sterile filtered through a 0.22-μm filter. The final drug product was filled into 2 ml of pre-sterilized, polypropylene, gasketed micro-centrifuge tubes at Nationwide Children’s Hospital and stored at −60 °C or lower as a frozen liquid. Further details and the structure of the product are provided in Extended Data Fig. 1, and additional information is provided in US patent 10689625, which covers composition claims for PR006 vector and rAAV.

#### Progranulin ELISA

Cell lines were seeded at an equal density and differentiated into neurons for a period of 7 d. On day 7, neurons were transduced with the indicated amounts of PR006 virus or excipient alone (0 vg per cell) and incubated at 37 °C for 72 h. Progranulin levels in cell media or lysates were determined by an ELISA assay (AdipoGen human progranulin ELISA kit, AG-45A-0018YEK-KI01) according to the manufacturer’s instructions. A standard curve of human recombinant progranulin was run in parallel to the samples to determine the progranulin concentration (ng ml^−1^). Total protein concentration of cell lysates was measured by BCA assay (Pierce, 23225). Progranulin expression levels determined by ELISA in both cell lysates and cell media were normalized to volume.

#### Patient-derived iPSCs

Control (ND38555; WT), FTD-GRN 1 (ND50015; M1L) and FTD-GRN 2 (ND50060; R493X) iPSC lines were first induced into proliferating neural stem cell lines using Neural Induction Media consisting of Neurobasal Medium (21103-49) and Neural Induction Supplement (A16478-01). Neural stem cell lines were seeded at an equal density and differentiated into neurons for a period of 7 d. To obtain post-mitotic neurons, neural stem cells were seeded in Neuron Differentiation Media (Neurobasal Medium, B27 Supplement (17504-044), GlutaMAX-I Supplement (35050-061), CultureOne Supplement (33202-01) and ascorbic acid (A8960-5G) and allowed to differentiate into neurons after a 7–10-d incubation period. This differentiation strategy was adapted from commercially available protocols and methods developed by Thermo Fisher Scientific (formerly Life Technologies). On day 7, neurons were transduced with different amounts of PR006 virus or excipient alone at 37 °C for 72 h. All reagents were obtained from Thermo Fisher Scientific.

### Mouse model and analytics

All mouse studies were performed by PsychoGenics in accordance with approved procedures by the IACUC and the NIH Guide for the Care and Use of Laboratory Animals. Original *Grn*-KO breeders were obtained from The Jackson Laboratory (stock number 013175). *Grn*-KO mice were made by deleting exons 1–4 from the targeted granulin (*Grn*) locus. Animals were kept on a 12-h/12-h light/dark cycle with a room temperature of 20–23 °C and a relative humidity of 30–70%. All mice were housed on Optimice racks in an enriched environment containing an igloo (Nylabone) and nesting material. Food (Formulab Diet, 5001) and water were provided ad libitum for the duration of the study. Animals were weighed once per week and checked for survival twice per day. All assessments were performed during the animals’ light cycle phase.

#### Stereotaxic injections

*Grn*-KO animals were enrolled at approximately 19–20 weeks of age. Ten microliters of PR006 or excipient (vehicle; 20 mM Tris pH 8.0, 200 mM NaCl and 1 mM MgCl_2_ + 0.001% poloxamer 188) was administered per animal via a single unilateral ICV injection in the left hemisphere. Animals received 5 mg kg^−1^ carprofen subcutaneously immediately before surgery. Standard aseptic surgical procedures were used during the surgery. All injections were performed under isoflurane anesthesia (3–4% induction, 1–2% maintenance) using a 30-gauge cannula (PlasticOne) attached to the manipulator arm of the stereotaxic frame, connected to a 50-μl Hamilton syringe (gastight 1705) with 50-mm-length polyethylene tubing (PE-50 C313CT). A mid-line incision was made, and the periosteum was removed from the skull. A small hole was drilled in the skull at the stereotaxic coordinates (anterior posterior (AP): +0.3 mm and medial lateral (ML): +1.0 mm with respect to bregma) using a stainless steel burr attached to a micro-drill. The cannula was slowly lowered to dorsal ventral coordinate −3.0 mm to reach the ventricle (DV: −3.0 mm). A 10-μl volume of test article was delivered at a rate of 20 μl min^−1^. After infusion, the needle was left in place for 5 min to allow the test article to diffuse, and the needle was then slowly retracted over 1–2 min. The skin was sutured over the injection site, and the animal was returned to the home cage. Upon recovery from the anesthesia, mice received 0.1 mg kg^−1^ buprenorphine subcutaneously. Twenty-four hours after surgery, mice received a second dose of 5 mg kg^−1^ carprofen. Mice were observed twice daily for health and survival. Experimenters were blinded to study treatment assignments.

#### Tissue and biofluids collection

At study termination, animals were euthanized for tissue collection. Animals were first anesthetized by isoflurane for CSF collection. Whole blood was collected via closed cardiac puncture and placed into K-EDTA-coated microcentrifuge tubes. Whole blood was placed on wet ice for no longer than 30 min before being centrifuged. Plasma was collected and stored in dry ice. The animal was then decapitated. The brain was removed and hemisected. The left hemisphere was drop-fixed in chilled 4% paraformaldehyde for 24 h and stored at 4 °C. The samples were then washed with 1× PBS three times and stored in 1× PBS at 4 °C. The right hemisphere was dissected into individual brain regions: hippocampus, motor cortex, somatosensory cortex, rest of cortex, striatum, cerebellum, brainstem and rest of brain. Brain structures were snap frozen in liquid nitrogen. Several peripheral organs were also collected: gonads, kidney, heart, liver, lung and spleen.

#### Vector genome biodistribution

Genomic DNA extractions were performed across brain and peripheral tissues by Azenta Life Sciences (formerly GENEWIZ) using the PureLink Pro 96 Genomic DNA Kit (catalog number K182104A). To determine the amount of vector copies, the desired sequence was amplified using polymerase chain reaction (PCR) on a 384-well plate (Applied Biosystems, 4309849) and TaqMan Master Mix 2.0 (Applied Biosystems, 4396838). Forward and reverse primers were designed to bind to PR006 (PR006-F: GTCTTCCACGACTGTGGGAT, PR006-R: GTCAGGGCCACCCAGCTC) using the TaqMan probe (6FAM- CCGGTTGAGCCACC-ATGTGGACCC -TAMRA). Primers and probe were produced by Integrated DNA Technologies. A standard curve was established with a template control DNA with concentrations ranging from 0 to 4 × 10^5^ copies per microliter. All samples were run in triplicate in a 10-μl reaction mixture. The plate was briefly centrifuged at more than 2,000*g* for 3–5 min at room temperature and then placed in a QuantStudio qPCR system (Thermo Fisher Scientific). The amplification of the target amplicon based on fluorescence from all samples in the assay plate was recorded, and the Ct value was determined by QuantStudio software. A standard curve was prepared by plotting the Ct of each standard versus the number of copies per reaction on a logarithmic scale (the final graph is a straight line fit by plotting values on a linear *y* axis and a log_10_ *x* axis). Vector copy numbers in each sample preparation were determined by interpolating Ct values with the standard curve determined. A Grubbs test was used to analyze triplicate samples for outliers. Replicates with a z-score of more than 1.15 were removed from the mean calculation for vector copies for a given sample. Each vector copy value was normalized to the amount of genomic DNA in the reaction and reported as vg µg^−1^ of genomic DNA.

#### *GRN* mRNA

Detection and quantitation of mRNA containing the codon-optimized progranulin (GRN) transgene was determined via reverse transcription quantitative PCR (RT–qPCR). Forward primer: TGACCGCGTTACTCCCACA, Reverse primer: CCAAAATGATGAGACAGCACAATAA and Probe: CTCCTGGGCAACGTGCT. All procedures were performed under blinded conditions. mRNA was extracted from the cortex using a QIAsymphony RNA Kit (Qiagen) according to the manufacturer’s instructions. Purity and concentration determinations of the extracted total RNA were performed on a NanoDrop 8000 (with 1× TE pH 8.0 as the blanking buffer). The concentration and purity (A260/A280 ratios) values for the tissue samples that were within the expected concentration and purity ranges of 2.5–3,000 ng μl^−1^ and 1.7–2.3, respectively, ranged from 5.426 ng μl^−1^ to 144.5 ng μl^−1^ and from 2.0 to 2.3, respectively. The total RNA eluates from tissue were set up and analyzed on a Qiagen QIAgility and a QuantStudio 7 Flex Real-Time System. Total RNA from tissue was tested at ≤0.5 μg in 5 μl in triplicate. All reactions were assembled on the 384-well plate in triplicate except for the quantification level of the standard curve (six replicates). Gene expression was normalized to the housekeeping gene Ppib.

#### Progranulin protein

Levels in tissues were measured after homogenization in RIPA buffer and quantification with an ELISA assay (developed in-house) in tissue lysate supernatants using human progranulin capture antibody mix for plate coating (R&D Systems, AF2420, at 1:100), and mouse anti-human progranulin detection antibody (R&D Systems, MAB2420, at 1:1,000). To normalize between samples, total protein levels were measured using the Pierce BCA protein assay. The final progranulin values reported are ng mg^−1^ protein (ng ml^−1^ progranulin in lysate divided by mg ml^−1^ total protein concentration). All samples were measured in triplicate. For technical replicate measurements, if at least one replicate measurement was below the lower limit of quantitation (LLOQ), the sample is reported as below quantitative limit. For graphing and statistical purposes, values below the LLOQ (below quantitative limit) were calculated as 0 ng mg^−1^. Data were acquired by microplate reader, Varioskan LUX, and analyzed using SkanIt RE 5.0.1 software (Thermo Fisher Scientific).

#### Iba1 and GFAP staining

Formalin-fixed paraffin-embedded sections were provided to QPS, which performed the immunofluorescence and quantitation^71^. Five sections per animal (total of 46 animals) were deparaffinized (10 min Roti-histol, 5 min Roti-histol/100% ethanol, 10 min 100% ethanol, 5 min 96% ethanol, 5 min 70% ethanol, 5 min 50% ethanol), washed with 1× PBS, blocked for 1 h at room temperature with 0.1% Triton X-100/PBS and labeled at room temperature for 45 min with the following antibodies: guinea pig polyclonal antibody against Iba1 (number 472, Synaptic Systems, 234004, at 1:500), detected with Cy3-labeled donkey anti-guinea pig polyclonal antibody (number 515, Jackson ImmunoResearch, 706-165-148, at 1:1000), and rabbit polyclonal antibody against GFAP (number 29; Dako, Z0334, at 1:500), detected with DyLight 650-labeled donkey anti-rabbit polyclonal antibody (number 319, Abcam, ab96922, at 1:500). Slides were washed with 1× PBS, incubated in secondary antibodies and counterstained with the nuclear dye DAPI for 1 h at room temperature. After incubation, the slides were washed with 1× PBS before being mounted.

#### Lipofuscin

The formalin-fixed brain (left hemisphere) from all animals and the WT control were shipped to Reveal Biosciences for histology and then to Alizée for microscopic evaluation. Samples were paraffin embedded following standard procedure. Formalin-fixed paraffin-embedded blocks were serially sectioned at 5 μm onto positively charged slides. One slide per level per sample was stained with hematoxylin and eosin (H&E) according to Reveal Biosciences’ standard staining protocol. A single H&E-stained slide from each animal was chosen based upon optimal visualization of all brain regions and scanned under fluorescence on an Olympus VS120 slide scanner for demonstration of autofluorescent lipofuscin granules. Semi-quantitative scoring of lipofuscin accumulation was performed by a blinded board-certified pathologist according to the following grading scheme: 0, no lipofuscin observed; 1, very small granules of lipofuscin (<2 μm) scattered throughout the region; 2, increased density of small granule accumulation and/or development of larger granules (>2–3 μm); 3, multifocal regions with a high density of lipofuscin granules visible from a low objective power; and 4, widespread lipofuscin accumulation.

#### Inflammation-related gene expression

The levels of the inflammation-related genes (Cd68 and Tnf) were measured in mouse cortex using qRT–PCR. The data were normalized to an internal standard (Gapdh and Ppib). The fold change in expression was calculated using the ΔΔCT method, and resulting values were normalized to the excipient-treated *Grn-*KO group. Blinded somatosensory cortex samples (previously stored at −80 °C) were homogenized, and RNA was extracted using a RNeasy Plus Mini Kit (Qiagen, 74136) according to the manufacturer’s instructions. RNA quantity and integrity were assessed by a NanoDrop spectrophotometer. RNA samples were standardized to 17.8 ng µl^−1^ concentrations and then reverse transcribed using a High-Capacity RNA-to-cDNA Kit (Applied Biosystems, 4387406), yielding 355.7 ng of cDNA for each sample (volume, 20 µl). The following primers were designed and purchased from Integrated DNA Technologies.

Gapdh-F: CAA GGT CAT CCA TGA CAA CTT TG, Gapdh-R: GGC CAT CCA CAG TCT TCT GG, Ppib-F: TGG AGA GCA CCA AGA CAG ACA, Ppib-R: TGC CGG AGT CGA CAA TGA T, Cd68-F: GGA CTA CAT GGC GGT GGA ATA, Cd68-R: GAT GAA TTC TGC GCC ATG AA, Tnf-F: CTG AGG TCA ATC TGC CCA AGT AC and Tnf-R: CTT CAC AGA GCA ATG ACT CCA AAG. The reactions were performed using the universal two-step RT–PCR cycling conditions. Primer specificity was verified using the Basic Local Alignment Search Tool (http://www.ncbi.nlm.nih.gov/blast/). RNA extracted from tissues was reverse transcribed into cDNA, and the resulting cDNA was used as a template for qPCR with primers specific to the genes of interest. Samples were run using PowerUp SYBR Green Master Mix (Applied Biosystems, A25780) in triplicate on a 384-well plate. Cycling conditions were as follows: 50 °C (2 min), 95 °C (10 min), 40 cycles of 95 °C (15 s), 60 °C (1 min), 95 °C (15 s) and 60 °C (15 s). For each target gene, the Ct value was normalized to an internal standard (Gapdh or Ppib). The fold change in expression was calculated using the ΔΔCT method, and resulting values were normalized to the mean of the *Grn*-KO + excipient group. In this experiment, Ppib was used as the internal standard for Ct value normalization analysis because the expression level of Ppib is closer to the target genes (Cd68 and Tnf) based on the Ct values.

#### RNA sequencing

RNA extraction, library preparation and sequencing were performed at GENEWIZ. Total RNA was extracted from fresh-frozen mouse cortex tissue samples using a Qiagen RNeasy Plus Universal Mini Kit following the manufacturer’s instructions. RNA quality was confirmed, and then poly(A) enrichment for mRNA and cDNA library preparation was performed. Sequencing libraries were analyzed on an Illumina HiSeq (2 × 150-bp reads). Reads were mapped to the *Mus musculus* genome, and differential expression analysis was performed. Library preparation with poly(A) selection and HiSeq sequencing: extracted RNA samples were quantified using a Qubit 2.0 Fluorometer (Life Technologies), and RNA integrity was checked using a TapeStation 4200 (Agilent Technologies). RNA sequencing libraries were prepared using the NEBNext Ultra RNA Library Prep Kit for Illumina following the manufacturer’s instructions (New England Biolabs). In brief, mRNAs were first enriched with oligo(dT) beads. Enriched mRNAs were fragmented for 15 min at 94 °C. First-strand and second-strand cDNAs were subsequently synthesized. cDNA fragments were end-repaired and adenylated at 3′ ends, and universal adapters were ligated to cDNA fragments, followed by index addition and library enrichment by limited-cycle PCR. The sequencing libraries were validated on the Agilent TapeStation and quantified by using the Qubit 2.0 Fluorometer as well as by quantitative PCR (Kapa Biosystems). The sequencing libraries were clustered on three lanes of a flowcell. After clustering, the flowcell was loaded on the Illumina HiSeq instrument (4,000 or equivalent) according to the manufacturer’s instructions. The samples were sequenced using a 2 × 150-bp paired-end configuration. Image analysis and base calling were conducted using HiSeq Control Software. Raw sequence data (.bcl files) generated from the Illumina HiSeq were converted into FASTQ files and de-multiplexed using Illumina’s bcl2fastq 2.17 software. Raw sequencing data were handed over to Prevail.

#### RNA sequencing analysis

Prevail received raw sequencing data from GENEWIZ and proceeded with further analysis. The mouse reference genome (GRCm38.p6) was modified to include the human *GRN* transgene. Reads were mapped to the modified reference genome using the STAR alignment tool in two-pass mode^72^. Aligned reads were then used to generate unique gene-level hit counts and used for downstream differential expression analysis. Differential expression analyses were carried out using DESeq2 following the authors’ best practices recommendations^73^. In brief, a counts matrix was generated for the experimental cohort. Genes with consistent low expression across the dataset (<10% of samples having reads per million of more than 10) were filtered. Differentially expressed genes were identified following a generalized linear model implemented in DESeq. We used GSVA^31^ to measure and compare activity levels of previously published gene signatures that are dysregulated in *Grn*-KO mice compared to WT. Specifically, we identified gene sets reported in two previously published studies^13,15^ demonstrating deficiencies in lysosomal and immune-related pathways in mice lacking *Grn*. We used the GSVA methodology for scoring activity states for these curated gene sets. Statistical analyses were performed to determine the effect of PR006 dosing on the activity states. Primary analysis was performed using ANOVA to test differences in homogeneity of population means and to confirm a model effect of the excipient-treated group when compared to age-matched WT animals. Dunnett’s test was used to test for differences in the PR006-treated animals against the excipient-treated group while controlling for the family-wise type I error rate, which kept the family-wise type I error rate at 0.05 rather than 0.15 as implied by the number of comparisons conducted.

### Animal safety study

The toxicology of PR006 was evaluated in cynomolgus macaques, an NHP species, in a study conducted by Labcorp under GLP conditions. Labcorp is fully accredited by the AAALAC. All procedures in the protocol were in compliance with applicable animal welfare acts and were approved by the local IACUC. The study design was based on the principles of the US Food and Drug Administration CDER/ICH-M3(R2), Nonclinical Safety Studies for the conduct of Human Clinical Trials and Marketing Authorization for Pharmaceuticals (CDER, January 2010). PR006 was administered once via ICM injection in cynomolgus monkeys with a 6-d, 29-d or 182-d post-administration observation period; animals were euthanized at day 30 or day 183. Animals were dosed with either a high dose (3.9 × 10^10^ vg g^−1^ brain, the maximum feasible dose achievable with 1.2 ml volume) of PR006 or a lower dose (3.9 × 10^9^ vg g^−1^ brain). The study also included a control arm in which animals received 1.2 ml of excipient only (20 mM Tris pH 8.0, 200 mM NaCl and 1 mM MgCl_*2*_ + 0.001% (w/v) poloxamer 188). Cynomolgus NHPs were assessed by multiple in-life observations and measurements, including mortality/morbidity (daily), clinical observations (daily), body weight (baseline and weekly thereafter), visual inspection of food consumption (daily), neurological observations, indirect ophthalmoscopy and electrocardiographic measurement. Analysis of neutralizing antibodies to the AAV9 capsid was performed at baseline and at euthanization on day 30 or day 183. Clinical pathology consisting of hematology, coagulation, clinical chemistry and urinalysis was performed at baseline (blood tests; once for urinalysis) and once during weeks 1 and 13 of the dosing phase. Animals were euthanized and tissues harvested on day 30 or day 183. The tissues were divided into replicates. One replicate was preserved in 10% neutral-buffered formalin for histopathological evaluation. Additional replicates were collected for qPCR and transgene expression analysis.

### Phase 1/2 study design

Study PRV-FTD101 (NCT04408625) is a phase 1/2, multi-center, open-label, ascending-dose, first-in-human study evaluating the safety, tolerability, immunogenicity, effects on progranulin levels, effects on biomarkers and efficacy parameters of PR006 in participants with FTD with progranulin mutations.

Each patient had to meet all the following criteria to be enrolled in this study:

1. Men or women aged 30–80 years (inclusive) at the time of informed consent
2. Body weight range of ≥40 kg (88 lbs) to ≤110 kg (242 lb) and a body mass index of 18–34 kg m^−2^
3. Has symptomatic FTD as per investigator assessment (behavioral-variant FTD (bvFTD), primary progressive aphasia (PPA)-FTD, FTD with corticobasal syndrome or a combination of syndromes were allowed for enrollment)
4. Score ≥1 and ≤15 on CDR plus NACC FTLD sum of boxes
5. Stable use of background medications at least 8 weeks before investigational product dosing
6. Carrier of a pathogenic GRN mutation confirmed by the central laboratory
7. Negative screening test for *Mycobacterium tuberculosis* (MTB) or documented negative MTB test within 1 year before screening
8. Age-appropriate and gender-appropriate cancer screenings are up to date and completed as per the investigator’s judgment and local standard of care before screening.
9. Patient and/or patient’s legally authorized representative (where applicable by local regulation) has the ability to understand the purpose and risks of the study and provide written informed consent and authorization to use protected health information in accordance with national and local privacy regulations. The patient or legally authorized representative may also provide consent for future biomedical research in accordance with their national regulations; however, the patient may still participate in the study without providing consent for future biomedical research.
10. Patient has a reliable study partner/informant (for example, family member or friend) willing and able to participate in the study as a source of information on the patient’s health status and cognitive and functional abilities (including providing input into the rating scales). The study partner should have regular contact with the patient (in person or via phone/video communication). The study partner must sign a separate partner informed consent form indicating that she/he understands the study requirements and is willing to participate and attend study visits requiring study partner input.
11. Women of non-childbearing potential must be either surgically sterile (hysterectomy, bilateral tubal ligation, salpingectomy and/or bilateral oophorectomy at least 26 weeks before screening) or be post-menopausal, defined as spontaneous amenorrhea for at least 2 years, with follicle-stimulating hormone level in the post-menopausal range at screening based on the central laboratory’s range.
12. Men and women of childbearing potential (that is, ovulating, pre-menopausal and not surgically sterile) must use a highly effective method of contraception consistently and correctly for the duration of the study, including the long-term follow-up.
13. Men must agree to abstain from sperm donation for the duration of the study, including long-term follow-up.
14. Women must agree to abstain from egg donation for the duration of the study, including long-term follow-up.
15. Women of childbearing potential cannot be pregnant or lactating/breastfeeding and must have a negative result for the serum pregnancy test (β-human chorionic gonadotropin) at screening.
16. Patient is generally ambulatory and not dependent on a walker or wheelchair.
17. Patient is living in the community (that is, not in a nursing home); some levels of assisted living may be permitted at the discretion of the investigator.
18. Pneumococcal pneumonia and shingles vaccines are required within 10 years of screening (allowed to be performed during screening but must be given at least 4 weeks before initiation of immunosuppressant regimen).

Patients meeting any of the following criteria were excluded from the study:

1. Diagnosis of a significant CNS disease other than FTD that may be a cause for the patient’s FTD symptoms or may confound study objectives
2. Brain MRI/magnetic resonance angiography imaging indicating clinically significant abnormality, including evidence of prior hemorrhage, infarct larger than 1 cm^3^ or more than three lacunar infarcts or a structural or vascular abnormality deemed a contraindication to intracisternal injection
3. Hypersensitivity or contraindications to corticosteroid and/or sirolimus use
4. Clinical evidence of peripheral symmetric sensory polyneuropathy
5. Concomitant disease or condition within 6 months of screening that could interfere with, or treatment of which might interfere with, the conduct of the study or that would, in the opinion of the investigator, pose an unacceptable safety risk to the patient or interfere with the patient’s ability to comply with study procedures, including, but not limited to, the following: (a) evidence of clinically significant liver disease; (b) unstable autoimmune disease requiring chronic immunosuppression; (c) poorly controlled/not adequately managed diabetes (screening hemoglobin A1c (HbA1c) ≥7%); (d) history of unstable angina, myocardial infarction, chronic heart failure (New York Heart Association Class III or IV) or clinically significant conduction abnormalities (for example, unstable atrial fibrillation) within 1 year before screening; (e) clinically significant 12-lead electrocardiographic abnormalities at screening, as determined by the investigator; (f) uncontrolled hypertension defined as average of three systolic blood pressure (SBP)/diastolic blood pressure (DBP) readings >165/100 mm Hg at screening or persistent SBP/DBP readings >180/100 mm Hg within 3 months before screening that, in the opinion of the investigator, are indicative of chronic uncontrolled hypertension; (g) history of cancer within 5 years of screening or current presence of pre-cancerous lesions, with the exception of fully excised non-melanoma skin cancers and fully excised prostate carcinoma in situ that have been stable for at least 6 months; (h) history or current alcohol or drug abuse within 2 years of screening; (i) any current psychiatric diagnosis according to the Diagnostic and Statistical Manual of Mental Disorders, Fifth Edition, the International Statistical Classification of Diseases and Related Health Problems, Tenth Revision, or equivalent that may interfere with the patient’s ability to perform study procedures and all assessments (for example, psychosis, major depression, bipolar disorder, mental retardation and schizophrenia) (note: psychiatric manifestations of FTD are not exclusionary); (j) at imminent risk of self-harm, based on clinical interview and responses on the Columbia Suicide Severity Rating Scale (C-SSRS). Patient must be excluded if they report ideation with intent, with or without a plan or method (that is, positive response to item 4 or 5 on the C-SSRS), in the past 2 months or suicidal behavior in the past 6 months; (k) any medical disorders that, in the opinion of the investigator, could interfere with study-related procedures (including safe performance of lumbar puncture or intracisternal injection), such as prohibitive spinal diseases, bleeding diathesis, clinically significant coagulopathy, thrombocytopenia or increased intracranial pressure; (l) documented stroke or transient ischemic attack within 1 year before screening; (m) history of seizure or unexplained blackouts, with the exception of seizure due to known, transient cause (for example, medication or electrolyte disturbance), within 10 years before screening; (n) currently active infection or severe infection (for example, pneumonia, septicemia or CNS infections (for example, meningitis or encephalitis)) within 12 weeks before screening; (o) history of severe allergic or anaphylactic reactions or history of hypersensitivity to any inactive ingredient of the investigational product (refer to the investigator’s brochure) or protocol-required immunosuppressant medications; (p) clinical evidence of vitamin B12 deficiency or vitamin B12 level less than the lower limit of normal if deemed clinically significant as per the investigator’s assessment at screening; (q) history of neurosyphilis or history of syphilis infection without documentation of adequate treatment; and (r) patient is generally frail or has any medical condition, for which, in the view of the investigator, participation in the study would not be in the best interest of the patient or is likely to prohibit further participation during the study period.
6. Clinically significant abnormalities in laboratory test results at screening as given below (laboratory testing may be repeated with medical monitor approval): (a) total bilirubin, alanine aminotransferase or aspartate aminotransferase more than 1.5× the upper limit of normal (ULN) (note: patients with confirmed Gilbert syndrome are allowed for enrollment with sponsor’s agreement); (b) serum creatinine more than 1.5× ULN; (c) hematocrit less than 35% for men and less than 32% for women; (d) absolute neutrophil count less than 1,500 per microliter; (e) platelet count less than 100,000 per microliter; (f) international normalized ratio more than 1.4 or other coagulopathy; (g) activated partial thromboplastin time more than 50 s; (h) thyrotropin level outside the normal range and deemed clinically significant by the investigator; (i) positive result for hepatitis B surface antigen, hepatitis C antibody or HIV 1 or 2; and (j) any other abnormal screening laboratory test result deemed clinically significant by the investigator.
7. Participation within 3 months before screening in another therapeutic investigational drug or device study with purported disease-modifying effects on FTD, unless it can be documented that the patient received placebo only
8. Any type of prior gene or cell therapy
9. Immunizations (live vaccines) in the 4 weeks before screening. Note: pneumococcal vaccine and shingles vaccine administration are allowed during the screening period (patients not previously vaccinated should receive pneumococcal and/or shingles vaccine administration at least 4 weeks before sirolimus loading dose).
10. Use of blood thinners (for example, warfarin, heparin and novel oral anticoagulants) in the 2 weeks before screening or the anticipated need to initiate blood thinners during the study. Antiplatelet therapies (prophylactic aspirin and clopidogrel) are acceptable if the patient is medically able to temporarily stop from at least 7 d before and at least 48 h after intracisternal injection and lumbar puncture.
11. Contraindications or intolerance to imaging methods (MRI and computed tomography) inducing claustrophobia and intolerance to contrast agents used for MRI or computed tomography (including, but not limited to, gadolinium contrast agents and iohexol)
12. Contraindications to general anesthesia or deep sedation
13. Positive urine test for drugs of abuse (including opiates, amphetamines, cocaine, barbiturates and phencyclidine) without prescription at screening and day −1.

The protocol was updated for several amendments. The amendments were necessary to introduce prophylactic immunosuppression regimen adjustments and safety monitoring clarification (monitoring for DRG toxicity and mitigation of venous thrombotic events).

Patients were reimbursed for expenses incurred for travel, accommodation and meals related to the study visits. Some patients also received a small stipend for certain study visits. Patient reimbursement was reviewed and approved by IRBs and ethics committees.

The original protocol contemplated three dose-escalating cohorts, each enrolling five patients. Being a first-in-human study in a rare disease, the sample size was based on practical, not statistical, considerations. The high-dose cohort was not enrolled, because the mid-dose cohort achieved and exceeded the intended elevations of CSF progranulin levels, and three patients recruited for this cohort were re-allocated to the lower-dose cohorts. Overall, 13 patients participated in the study. Patients were enrolled between 9 December 2020 and 7 March 2023.

For clinical study data reporting, we followed the rules for a pre-specified interim analysis after all patients in the low-dose cohort reach year 1 as mandated by the study protocol and as per established CONSORT guidelines^74^. As typical for a phase 1/2 study, the primary endpoints focused on safety and pharmacodynamic readouts, with secondary and exploratory endpoints directed at additional biomarker readouts. Study objectives (baseline to year 1) are listed below:

Primary:

- Evaluate the safety, tolerability and immunogenicity of three dose levels of PR006A administered via suboccipital injection into the cisterna magna
- Quantify progranulin levels in blood and CSF

Secondary:

To evaluate the effect of PR006A on:

- CDR plus NACC FTLD
- NfL levels in blood and CSF

Exploratory:

To evaluate the effect of PR006A on:

- Measures of cognition, behavior, language and daily living
- Viral shedding
- Imaging patterns based on volumetric MRI and quantification of white matter lesions
- Selected biomarkers of neuroinflammation, astroglial pathology and lysosomal function (for example, GFAP, YKL40 and BMP in CSF, blood and urine)

Given the open-label design, the protocol did not pre-specify quantitative effect sizes. The study followed accepted guidelines for inclusion and ethics and was conducted under oversight by regulatory agencies of countries with trial sites and by an independent data monitoring committee. IRB approval was obtained from the UCSF Medical Central IRB; the Advarra IRB; the University of Pennsylvania IRB; South Central – Oxford A; CEIC de Euskadi; CEIM Hospital Clinic de Barcelona; UZ Leuven – Commissie Medische Ethiek – toetsingscommissie; and the SLHD Ethics Review Committee (RPAH Zone). Informed consent was obtained from all participants.

The PR006 low dose of 2.1 × 10^13^ vg was selected as the starting clinical dose as it corresponds to a dose at which robust therapeutic benefits were seen in an animal model, maintains a reasonable margin of exposure to the non-adverse NHP toxicology findings and takes into consideration the severity and absence of treatment options for FTD-GRN. The dose corresponds to 1.6 × 10^10^ vg g^−1^ brain weight, assuming an adult brain mass of 1.3 kg (ref. ^75^). The mid dose of 4.2 × 10^13^ vg represents a 2× increase over the low dose. Titering methodology was updated during the course of the study, and the presented dose values are based on droplet digital PCR methodology.

The dose rationale was based on three considerations: first, published human clinical, biomarker and genetic data on GRN mutations and their causative effects in FTD; second, non-clinical efficacy, biodistribution and safety data obtained in a mouse model of FTD-GRN; and third, biodistribution and safety data obtained in NHP toxicity studies. (1) Patients with FTD-GRN carry a germline mutation in one of the two chromosomal alleles of the GRN gene, which encodes the progranulin protein, resulting in haploinsufficiency with a corresponding reduction of normal progranulin levels by approximately 50%^4–7^. The goal of the PR006 therapy was, thus, to express transgene-derived progranulin to alleviate the pathological consequences of the diminished levels of endogenous progranulin. (2) As described in detail in the subsections above, the efficacy of PR006 treatment was evaluated in a dose-ranging study in *Grn*-KO mice using various pathological outcome measures. The mouse efficacy data indicate that the mid dose of 1.6 × 10^10^ vg g^−1^ brain of PR006 significantly elevates progranulin in the brain and ameliorates essential pathological features seen in FTD-GRN, such as neuroinflammation and lipofuscin deposition. (3) The safety and toxicity studies with PR006 conducted in GLP NHP studies, with ICM administration, the intended route of administration in the human study, established the dose of 3.9 × 10^10^ vg g^−1^ brain as no-observed-adverse-effect-level (NOAEL), providing a safety margin of 2.4× to the clinical starting dose.

PR006 was administered as a single dose via suboccipital injection into the cisterna magna by an interventional radiologist or a neurosurgeon. Before injection, a volume of ICM fluid equivalent to the PR006 dosing volume was removed. The procedure was performed under general anesthesia or deep sedation and using imaging guidance. Participants remained under observation for a minimum of 24 h after PR006 administration. All patients received immunosuppressant treatment. Three regimens were used: corticosteroids and sirolimus (*n* = 1, first patient dosed) and corticosteroids, rituximab and sirolimus (*n* = 11) or corticosteroids only (*n* = 1, last patient dosed) depending on the study protocol version and driven by the emerging data on AAV-based immunogenicity and DRG toxicity and benefit–risk profile of immunosuppressant use in the study patient population. The doses of the immunosuppressants were as follows: methylprednisolone 1,000 mg intravenous pulse on day −1, followed by prednisone 30 mg per orally for 14 d and then tapered over the ensuing 7 d; rituximab 1,000 mg intravenously within 3 d of PR006 administration; and sirolimus oral loading dose of 6 mg on day −1, followed by 2 mg per day for 90 d with tapering off over the ensuing 15–30 d. At the investigator’s discretion and upon discussion with the study sponsor, higher doses or a longer taper of corticosteroids and sirolimus were used for some patients. Patients who received only corticosteroids were treated with 1,000 mg of methylprednisolone intravenously on day −1, followed by 500 mg intravenously bimonthly four times and then a final dose 1 month later.

### Clinical biomarker analysis

#### Progranulin ELISA

Progranulin expression in human CSF and plasma was determined by an ELISA assay (AdipoGen human progranulin ELISA kit, AG-45A-0018YEK-KI01). The minimum required dilutions for CSF and plasma for sample analysis were four-fold and 64-fold, respectively. The standard curve of the assay ranges from 0.125 ng ml^−1^ to 4.00 ng ml^−1^ (with an anchor point at 0.0625 ng ml^−1^ for the plasma assay). Three levels of quality control (QC) samples were included with each run. The ELISA was run in accordance with the manufacturer’s instructions. In brief, microtiter plates were pre-coated with polyclonal progranulin antibodies. Samples, standards and QCs were added and incubated on the plate. The plates were then washed, and the biotinylated polyclonal antibody specific to progranulin was added. After washing the plate, horseradish peroxidase-labeled streptavidin was added to the plate. In the final step, after plate washing, TMB was added. The reaction was stopped with an acidic solution. The plate was read where the color intensity is proportional to the concentration of progranulin in the samples.

#### Anti-drug antibody assays against AAV9 capsid and progranulin

For anti-AAV9 anti-drug antibody (ADA) assays, an ELISA immunoassay format was used. Samples, positive controls and negative controls were diluted to the minimum required dilution (1:50 for serum and 1:10 for CSF) and immobilized on a NUNC plate that was coated with AAV9 capsids and blocked with an assay diluent. For detection, peroxidase-conjugated Protein A/G and Protein L were added to the plate as a dual detection solution. ADAs that were present in the human samples formed a complex between the peroxidase-conjugated Protein A/G and Protein L reagents, which was detected by TMB substrate. The assay format used for the anti-progranulin ADA assays was the bridging immunogenicity assay, where total binding antibodies were detected using the MesoScale Discovery (MSD) electrochemiluminescent platform. In these assays, samples and positive and negative controls were incubated with labeled progranulin (biotin-PGRN and Sulfo-Tag-PGRN). If present, the ADA in the samples (CSF or serum) formed a bridge between the labeled biotin and Sulfo-Tag reagents. In the next step, the formed complex was bound to a blocked MSD-streptavidin (MSD-SA) plate and thus produced chemiluminescent signal that is generated when voltage is applied across the plate. The signal produced when the plate was read, in relative light units, was directly proportional to the amount of ADA present in the samples.

#### NfL levels

NfL levels were measured in CSF and plasma using a Quanterix Simoa digital immunoassay platform (NF-Light kit, 103186, and NF-Light Advantage V2 kit, 104073). The minimum required dilutions for CSF and blood analysis were 100-fold and four-fold, respectively. All calibrators, controls and buffers were prepared in accordance with the manufacturer’s instructions. In addition to controls provided by the manufacturer, buffer and matrix QCs were included in each run.

#### BMP levels

A liquid chromatography–tandem mass spectrometry (LC–MS/MS) method was validated for the quantitation of BMP in human urine. Internal standards were used for each assay. The working range of the assay was 0.05–100 ng ml^−1^. QC samples were prepared fresh at four levels. The parameters for the method validation included 3-d intra-assay and inter-assay accuracy and precision, sensitivity (specificity), recovery, matrix effect, dilution linearity and stability. Stability was evaluated after three freeze–thaw (F/T) cycles and short-term (bench top, room temperature) and long-term (frozen at −20 °C and −80 °C) storage. A SCIEX 7500 LC–MS/MS system was used for quantitation. The LC–MS/MS method met pre-set criteria and is suitable for the analysis of clinical study samples. The calibration curves were linear (*r* ≥ 0.99) with ≥75% of the standards ±15% (±20% at the LLOQ) of the theoretical spiked concentration. The 3-d intra-accuracy and inter-accuracy and precision at four QC concentrations were ±15% (±20.0% at the lowest QC) and ≤15% (≤20% at the lowest QC), respectively. There were no significant interfering peaks (≤20% of the lowest QC peak area) at the retention time of BMP and the internal standard. Recovery and matrix effect were reproducible. Dilution accuracy and precision were ±15% and ≤15%, respectively. BMP was stable after three F/T cycles, at room temperature for up to 6 h, refrigerated at 4 °C for up to 48 h, at −20 °C for up to 6 months and at 80 °C for up to 24 months. Normal healthy volunteer urine samples were procured in a custom collection project with BioIVT. The collection methods and assays performed were identical to those used for the clinical trial samples.

### Statistical analysis

Statistical methods used to analyze non-clinical data are described in the figure legends reporting these data.

For the clinical biomarker analysis, continuous variables were summarized using descriptive statistics (mean and s.e.m.). Values below the lLLOQ were analyzed using half of the value of the LLOQ, and values above the upper limit of quantification were analyzed using the upper limit of quantification. Change from baseline variables assigned baseline as the last non-missing assessment before the study treatment administration.

For the clinical safety analysis, AEs are summarized by frequency, ‘*n* (%)’, representing the number of patients, and events ‘[E]’, at each level of summarization. Percentages for AEs were calculated using the number of treated patients in the Safety Analysis Set. For immunogenicity assessed through anti-AAV9 and anti-GRN antibodies, the frequency of patients positive at baseline and who reported positive treatment-emergent antibodies after treatment are reported. Percentages for immunogenicity were calculated using the number of patients who reported any data as the applicable denominator.

### Reporting summary

Further information on research design is available in the Nature Portfolio Reporting Summary linked to this article.

## Online content

Any methods, additional references, Nature Portfolio reporting summaries, source data, extended data, supplementary information, acknowledgements, peer review information; details of author contributions and competing interests; and statements of data and code availability are available at 10.1038/s41591-024-02973-0.

### Supplementary information

Reporting Summary

Supplementary Data 1 Clinical trial protocol.

Supplementary Data 2 Summary of changes to the clinical trial protocol.

## Figures and Tables

**Extended Data Fig. 6:**
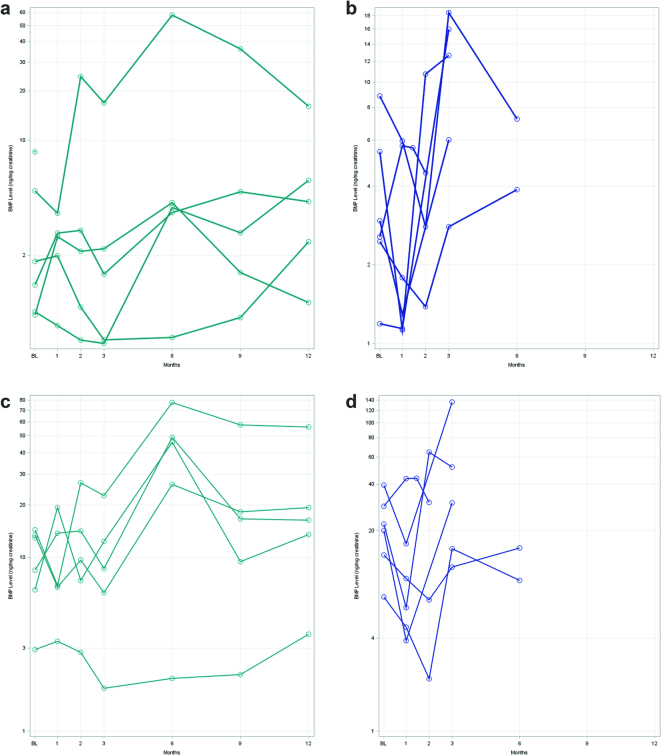
Individual subject adta plots of di-18:1 and di-22:6-BMP in urine following PR006 dosing of FTD-GRN patients. Urine samples were collected from study participants in the low dose (2.1 × 10^13^ vg, n=5) and mid dose 4.2 × 10^13^ vg, n=6) cohorts. Total (**a** = low dose, **b** = mid dose) di-18:1- and (**c** = low dose, **d** = mid dose) di-22:6-BMP levels were measured using a mass spectrometry-based assay and were normalized with creatinine concentrations (ng/mg). Mean (±SEM) values and statistical analyses are shown in Fig. 4 of the main text.

**Extended Data Table 1:**
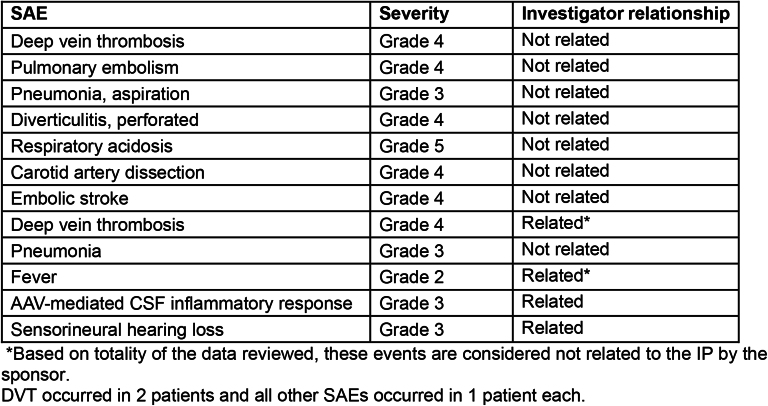
SAEs by severity and investigator-assigned causality

## Data Availability

For the clinical studies, individual participant data are protected by patient privacy laws and are stored on a secured network to which only appropriately trained and delegated staff have access. Any requests for raw and analyzed data should be sent in writing to the corresponding author, J.S. (sevigny_jeffrey@lilly.com), who will respond within 6 weeks of receipt. Clinical and non-clinical data and materials that can be shared will require approval from the sponsor and institutional review boards (where applicable) and a material transfer agreement. De-identified data will be transferred to the inquiring investigator by secure file transfer. The study protocol and statistical analysis plan were included with the submission.
