# Supplementary material for: Progranulin AAV gene therapy for frontotemporal dementia: translational studies and phase 1/2 trial interim results
Source: Nat Med. 2024 May 14;30(5):1406–15. doi: 10.1038/s41591-024-02973-0 (PMC11108785; doi:10.1038/s41591-024-02973-0)
Supplement: Supplementary file 1 — Reporting Summary [file 41591_2024_2973_MOESM1_ESM.pdf]

nature portfolio

Corresponding author(s): Jeffrey Sevigny

Last updated by author(s): Apr 6, 2024

Reporting Summary

Nature Portfolio wishes to improve the reproducibility of the work that we publish. This form provides structure for consistency and transparency in reporting. For further information on Nature Portfolio policies, see our Editorial Policies and the Editorial Policy Checklist.

Statistics

For all statistical analyses, confirm that the following items are present in the figure legend, table legend, main text, or Methods section.

n/a	Confirmed	
☐	☒	The exact sample size (*n*) for each experimental group/condition, given as a discrete number and unit of measurement
☐	☒	A statement on whether measurements were taken from distinct samples or whether the same sample was measured repeatedly
☐	☒	The statistical test(s) used AND whether they are one- or two-sided<br>*Only common tests should be described solely by name; describe more complex techniques in the Methods section.*
☐	☒	A description of all covariates tested
☐	☒	A description of any assumptions or corrections, such as tests of normality and adjustment for multiple comparisons
☐	☒	A full description of the statistical parameters including central tendency (e.g. means) or other basic estimates (e.g. regression coefficient) AND variation (e.g. standard deviation) or associated estimates of uncertainty (e.g. confidence intervals)
☐	☒	For null hypothesis testing, the test statistic (e.g. *F*, *t*, *r*) with confidence intervals, effect sizes, degrees of freedom and *P* value noted<br>*Give P values as exact values whenever suitable.*
☒	☐	For Bayesian analysis, information on the choice of priors and Markov chain Monte Carlo settings
☒	☐	For hierarchical and complex designs, identification of the appropriate level for tests and full reporting of outcomes
☒	☐	Estimates of effect sizes (e.g. Cohen's *d*, Pearson's *r*), indicating how they were calculated

Our web collection on statistics for biologists contains articles on many of the points above.

Software and code

Policy information about availability of computer code

Data collection	Standard commercially available softwares were used in these studies, including Microsoft, Word, Excel.<br>For data collection the QuantStudio and SkanIt software were used.
Data analysis	Statistical analysis was performed using SAS software Version 9.3.

For manuscripts utilizing custom algorithms or software that are central to the research but not yet described in published literature, software must be made available to editors and reviewers. We strongly encourage code deposition in a community repository (e.g. GitHub). See the Nature Portfolio guidelines for submitting code & software for further information.

Data

Policy information about availability of data

All manuscripts must include a data availability statement. This statement should provide the following information, where applicable:

- Accession codes, unique identifiers, or web links for publicly available datasets
- A description of any restrictions on data availability
- For clinical datasets or third party data, please ensure that the statement adheres to our policy

The primary data and additional details of methods underlying the results reported in this article will be made available upon request. For the clinical studies, individual participant data are protected by patient privacy laws and are stored on a secured network to which only appropriately trained and delegated staff have access. Any requests for raw and analyzed data should be sent in writing to the first author, J.S., and will be reviewed by the Institutional Review Boards in an

expeditious fashion. Data and materials (including the clinical trial protocol) that can be shared will require approval from the Institutional Review Boards and a material transfer agreement. De-identified data will be transferred to the inquiring investigator by secure file transfer. The study protocol and statistical analysis plan have been included with the submission.

Research involving human participants, their data, or biological material

Policy information about studies with human participants or human data. See also policy information about sex, gender (identity/presentation), and sexual orientation and race, ethnicity and racism.

Reporting on sex and gender	Sex of clinical study participants has been reported in Table 1 of the manuscript. Gender was not considered. Given the small number of participants, no sex- or gender-specific sub-analyses have been conducted.
Reporting on race, ethnicity, or other socially relevant groupings	Participating patients had a diagnosis of frontotemporal dementia and a confirmed mutation in the GRN gene. Given the small number of participants, no race, ethnicity, or socially relevant groupings were conducted
Population characteristics	Participating patients had a diagnosis of frontotemporal dementia and a confirmed mutation in the GRN gene.
Recruitment	Patients were recruited by the participating clinical trial sites. They were identified as carriers of mutations in the GRN gene. Given the small number of such patients, all potential participants who fulfilled the enrollment criteria were accepted into the study. There was no bias.
Ethics oversight	The study followed accepted guidelines for inclusion and ethics and was conducted under oversight by regulatory agencies of countries with trial sites and by an independent Data Monitoring Committee. Institutional Review Board (IRB) approval was obtained from UCSF Medical Central IRB, San Francisco, CA, USA, Advarra Institutional Review Board, Orlando, FL, USA, University of Pennsylvania Institutional Review Board, Philadelphia, PA, USA, South Central – Oxford A, London, UK, CEIC de Euskadi, Vitoria-gastelz, Spain, CEIM Hospital Clinic de Barcelona, Barcelona, Spain, UZ-Leuven – Commissie Medische Ethiek – toetsingscommissie, Leuven, Belgium, and SLHD Ethics Review Committee (RPAH Zone), Camperdown, Australia. Informed consent was obtained from all participants.

Note that full information on the approval of the study protocol must also be provided in the manuscript.

Field-specific reporting

Please select the one below that is the best fit for your research. If you are not sure, read the appropriate sections before making your selection.

☒ Life sciences ☐ Behavioural & social sciences ☐ Ecological, evolutionary & environmental sciences

For a reference copy of the document with all sections, see nature.com/documents/nr-reporting-summary-flat.pdf

Life sciences study design

All studies must disclose on these points even when the disclosure is negative.

Sample size	Animal studies used 8-10 animals per treatment group. This is a generally accepted sufficient sample size for animals studies with biochemical, or histochemical endpoints. Sample sizes were chosen based on the following publications showing a model effect in similar sample sizes 10.1016/j.neuroscience.2015.12.006, https://doi.org/10.1016/j.nbd.2021.105314 and treatment effect in other publications https://doi.org/10.1186/s12967-023-04251-y.
Data exclusions	No data were excluded in the analysis of studies reported in this manuscript.
Replication	iPSC experiment reported was repeated three times but with individual MOIs in the two additional studies not shown. Animal studies were conducted three times with slightly variable detailed conditions either in terms of the age of animals at dosing or the doses used.
Randomization	Animals used in preclinical studies were randomly assigned to experimental groups.<br>The clinical trial was an open-label study; randomization could thus not be implemented.
Blinding	In the preclinical studies, people conducting the studies were blinded to treatment groups. Unb linding took place only at the time of the final statistical analysis. Since the clinical study was an open-label study, there was no blinding. All mouse studies were performed by Psychogenics (Paramus, NJ), in accordance with approved procedures by IACUC and the NIH Guide for Care and Use of laboratory animals. . The toxicology of PR006 was evaluated in cynomolgus macaques, a non-human primates (NHP) species, in a study conducted by Labcorp (formerly Covance, under good laboratory practice (GLP) conditions. Labcorp Laboratories is fully accredited by the Association for Assessment and Accreditation of Laboratory Animal Care (AAALAC). All procedures in the Protocol were in compliance with applicable animal welfare acts and were approved by the local Institutional Animal Care and Use Committee (IACUC). The study design was based on the principles of the Food and Drug Administration Center for Drug Evaluation and Research (CDER)/International Conference on Harmonisation (ICH) Harmonised Tripartite Guidelines ICH-M3(R2)

Reporting for specific materials, systems and methods

We require information from authors about some types of materials, experimental systems and methods used in many studies. Here, indicate whether each material, system or method listed is relevant to your study. If you are not sure if a list item applies to your research, read the appropriate section before selecting a response.

Materials & experimental systems

n/a	Involved in the study
☐	☒ Antibodies
☐	☒ Eukaryotic cell lines
☒	☐ Palaeontology and archaeology
☐	☒ Animals and other organisms
☐	☒ Clinical data
☒	☐ Dual use research of concern
☒	☐ Plants

Methods

n/a	Involved in the study
☒	☐ ChIP-seq
☒	☐ Flow cytometry
☒	☐ MRI-based neuroimaging

Antibodies

Antibodies used	Measurement of progranulin in human CSF according to the manufacturer's instructions: (Adipogen Human Progranulin ELISA kit, Cat#: AG-45A-0018YEK-KI01). Measuremen t of Nfl in human CSF and plasma: NF-Light kit product #: 103186 and NF-Light Advantage V2 kit product #: 104073.<br>The following antibodies were used in animal studies according to manufacturer's instructions: Human progranulin capture antibody mix for plate coating (R&D Systems Cat #: AF2420 1:100), mouse anti-human progranulin detection antibody (Cat #: MAB2420 at 1:1000), guinea pig polyclonal antibody against Iba1 (#472; Synaptic Systems, 234004 at 1:500), detected with Cy3-labeled donkey anti-guinea pig polyclonal antibody (#515; Jackson ImmunoResearch, 706-165-148 at 1:1000), Rabbit polyclonal antibody against GFAP (#29; Dako, Z0334 at 1:500), detected with DyLight 650-labeled donkey anti-rabbit polyclonal antibody (#319; abcam, ab96922 at 1:500)
Validation	The antibodies used for clinical studies have been validated by the manufacturers: https://www.biomol.com/products/assay-kits/elisa-and -immunoassays/progranulin-human-elisa-kitag-45a-0018yek-ki01, https://www.quanterix.com/wp-content/uploads/2020/12/NF-light-Data-Sheet-HD-1%E2%88%95HD-X-2.pdf, https://www.quanterix.com/wp-content/uploads/2022/11/NF-light-Advantage-V2-HD-1-HD-X-Data-Sheet-Rev02.pdf. For the antibodies used for histochemical staining, the authors relied on validation given by the provider as seen on their website and did not conduct any additional validation experiments.

Eukaryotic cell lines

Policy information about cell lines and Sex and Gender in Research

Cell line source(s)	FTD-GRN #1 (Mll; ND50015), FTD-GRN #2 (R493X; ND50060) and an aged-matched control 425 subject (Control; ND38555) iPSC cell lines were used. All cell lines were obtained from the NINDS human genetics DNA and cell line repository
Authentication	Certificates of analysis were provided for all 3 lines from the NINDS cell line repository.
Mycoplasma contamination	All three cell lines were negative for mycoplasma contamination according to the certificate of analysis
Commonly misidentified lines<br>(See ICLAC register)	not used

Animals and other research organisms

Policy information about studies involving animals; ARRIVE guidelines recommended for reporting animal research, and Sex and Gender in Research

Laboratory animals	Mice studies used wild-type mice or Grn knockout mice at 4 months of age and at 14-16 months of age. NHP toxicity studies used young adult cynomolgus macaque animals (28-48 months old).
Wild animals	The study did not use wild animals
Reporting on sex	All studies enrolled both male and female animals across treatment groups.
Field-collected samples	The studies did not included field-collected samples.
Ethics oversight	IACUC approval was obtained for all animal studies. All mouse studies were performed by Psychogenics (Paramus, NJ), in accordance with approved procedures by IACUC and the NIH Guide for Care and Use of laboratory animals. The toxicology of PR006 was evaluated in cynomolgus macaques, a non-human primates (NHP) species, in a study conducted by Labcorp (formerly Covance, under good laboratory practice (GLP) conditions. Labcorp Laboratories is fully accredited by the Association for Assessment and Accreditation of Laboratory Animal Care (AAALAC). All procedures in the Protocol were in compliance with applicable animal welfare acts and were approved by the local Institutional Animal Care and Use Committee (IACUC). The study design was based on the principles of the Food and Drug Administration Center for Drug Evaluation and Research (CDER)/International Conference on Harmonisation (ICH) Harmonised Tripartite Guidelines ICH-M3(R2)

Note that full information on the approval of the study protocol must also be provided in the manuscript.

Clinical data

Policy information about clinical studies

All manuscripts should comply with the ICMJE guidelines for publication of clinical research and a completed CONSORT checklist must be included with all submissions.

Clinical trial registration	NTCT04408625
Study protocol	The study protocol is provided to all participating and interested clinical investigators. It will be provided to interested parties, following reasonable requests. It has not been publicly disclosed so far, but is included as requested by the editors as supplementary material
Data collection	Data are centrally collected at Prevail Therapeutics.
Outcomes	The clinical study was a FIH Phase 1/2 study and there were no pre-specified outcome measures.

Plants

Seed stocks	not applicable
Novel plant genotypes	not applicable
Authentication	not applicable
