# Supplementary material for: Progranulin AAV gene therapy for frontotemporal dementia: translational studies and phase 1/2 trial interim results
Source: Nat Med. 2024 May 14;30(5):1406–15. doi: 10.1038/s41591-024-02973-0 (PMC11108785; doi:10.1038/s41591-024-02973-0)
Supplement: Supplementary file 2 — Clinical trial protocol. [file 41591_2024_2973_MOESM2_ESM.pdf]

CLINICAL STUDY PROTOCOL
IND 019511
EUDRACT 2019-003159-12
A Phase 1/2 Ascending Dose Study to Evaluate the Safety and Effects on Progranulin Levels of PR006A in Patients with Fronto-Temporal Dementia with Progranulin Mutations (FTD-GRN)

PROTOCOL NO. PRV-FTD101

**Sponsor:**	Prevail Therapeutics, Inc.<br>430 East 29th Street, Suite 1520<br>New York, NY 10016, USA
**Version of Protocol:**	4.0
**Date of Protocol:**	15 July 2020
**Previous Version:**	Version 3.0 (22 April 2020)

For protocol-specific Sponsor and vendor contact information, please refer to the Investigator file.

CONFIDENTIAL

All financial and nonfinancial support for this study will be provided by Prevail Therapeutics, Inc. The concepts and information contained in this document or generated during this study are considered proprietary and may not be disclosed in whole or in part without the expressed, written consent of Prevail Therapeutics, Inc.

This study will be conducted according to the International Council for Harmonisation harmonised tripartite guideline E6(R2): Good Clinical Practice.

Protocol Approval – Sponsor Signatory

**Study Title**	A Phase 1/2 Ascending Dose Study to Evaluate the Safety and Effects on Progranulin Levels of PR006A in Patients with Fronto-Temporal Dementia with Progranulin Mutations (FTD-GRN)
**Protocol Number**	PRV-FTD101
**Version**	4.0
**Protocol Date**	15 July 2020

Protocol accepted and approved by:

Chief Medical Officer

Jeffrey Sevigny, MD

Prevail Therapeutics, Inc.
430 East 29th Street, Suite 1520
New York, NY 10016, USA

Signature

15 July 2020
Date

Declaration of Investigator

I have read and understood all sections of the protocol entitled "A Phase 1/2 Ascending Dose Study to Evaluate the Safety and Effects on Progranulin Levels of PR006A in Patients with Fronto-Temporal Dementia with Progranulin Mutations (FTD-GRN)" and the accompanying investigator's brochure.

I agree to supervise all aspects of the protocol and to conduct the clinical investigation in accordance with the protocol, the International Council for Harmonisation harmonised tripartite guideline E6(R2): Good Clinical Practice and all applicable government regulations. I will not make changes to the protocol before consulting with Prevail Therapeutics, Inc. or implement protocol changes without independent ethics committee approval except to eliminate an immediate risk to patients. I agree to administer study treatment only to patients participating in the clinical investigation under my personal supervision or the supervision of a subinvestigator.

I will not supply the investigational drug to any person not authorized to receive it. Confidentiality will be protected. Patient identity will not be disclosed to third parties or appear in any study reports or publications.

I will not disclose information regarding this clinical investigation or publish results of the investigation without authorization from Prevail Therapeutics, Inc.

__________________________________ ______________________

Signature of Principal Investigator Date

____

Printed Name of Principal Investigator

Table of Contents

Table of Contents 4
List of Tables 8
Protocol Synopsis 10
List of Abbreviations 27
1 Introduction 30
1.1 Background of Disease 30
1.1.1 Background on PR006A 32
1.1.2 Nonclinical Overview 33
1.2 Study Rationale 37
1.2.1 Overview 37
1.2.2 Rationale for Patient Population 37
1.2.3 Rationale for Staggering of Patient Enrollment 38
1.2.4 Rationale for Route of Administration 39
1.2.5 Rationale for Dosing 40
1.3 Risk/Benefit 43
2 Study Objectives and Endpoints 46
3 Investigational Plan 50
3.1 Study Design 50
3.1.1 Year 1 50
3.1.2 Post-Year 1 52
4 Patient Selection and Withdrawal Criteria 53
4.1 Selection of Study Population 53
4.1.1 Inclusion Criteria 53
4.1.2 Exclusion Criteria 55
4.2 Withdrawal of Patients from Study Treatment and/or the Study 59
4.2.1 Reasons for Withdrawal/Discontinuation 60
4.2.2 Handling of Withdrawals 60
4.2.3 Replacements 61
4.2.4 Study Stopping Rules 61
5 Study Treatments 63

5.1 Method of Assigning Patients to Treatment Groups 63
5.2 Treatments Administered 63
5.3 Identity of Investigational Product 63
5.4 Management of Clinical Supplies 63
5.4.1 Investigational Product Packaging and Storage 63
5.4.2 Investigational Product Preparation and Dispensing 63
5.5 Investigational Product Administration 64
5.5.1 Immunosuppressant Administration 64
5.5.1.1 Corticosteroid Administration 64
5.5.1.2 Sirolimus Administration 64
5.5.1.3 Immunosuppression Monitoring Criteria 64
5.5.2 Pre-Cisternal Puncture Procedures 65
5.5.3 Intracisternal Injection 65
5.5.4 Investigational Product Accountability 66
5.5.5 Other Supplies 66
5.5.6 Overdose Management 66
5.5.7 Product Quality Complaint 67
5.6 Treatment Compliance 67
5.7 Prior and Concomitant Therapy 67
5.7.1 Excluded Medications and/or Procedures 67
5.7.2 Restricted Medications and/or Procedures 68
5.7.3 Documentation of Prior and Concomitant Medication Use 69
6 Study Assessments and Procedures 70
6.1 Efficacy Assessments 70
6.1.1 CDR Plus NACC FTLD 70
6.1.2 Montreal Cognitive Assessment 71
6.1.3 Benson Complex Figure Copy and Recall 71
6.1.4 Clinical Global Impressions – Severity/Improvement 72
6.1.5 Trail Making Test, Part A and Part B 72
6.1.6 Digit Span Test (Forwards and Backwards) 72
6.1.7 Category Fluency 73
6.1.8 Multilingual Naming Test 73
6.1.9 Magnetic Resonance Imaging Volumetric Measures 73

6.2 Safety Assessments 74
6.2.1 Adverse Events 74
6.2.1.1 Definitions of Adverse Events 74
6.2.1.2 Serious Adverse Events 76
6.2.1.3 Eliciting and Documenting Adverse Events 76
6.2.1.4 Reporting Adverse Events 77
6.2.1.5 Suspected Adverse Reactions/Adverse Reactions 78
6.2.1.6 Reporting Serious Adverse Events 78
6.2.1.7 Expedited Reporting Requirements 78
6.2.1.8 Suspected Unexpected Serious Adverse Reactions 79
6.2.1.9 Assessment of Severity 79
6.2.1.10 Assessment of Causality 80
6.2.1.11 Follow-Up of Patients Reporting Adverse Events 81
6.2.2 Risk Mitigation Plan 81
6.2.2.1 Mitigation of Risks Associated with Lumbar Puncture 81
6.2.2.2 Risks Associated with the Use of Immunosuppressants 82
6.2.2.3 Risks Associated with Suboccipital Injection into the Cisterna Magna ..84
6.2.2.4 Risks Associated with AAV9-Based Therapy 85
6.2.2.5 Risks Associated with *GRN* Expression 86
6.2.2.6 Risks Associated with the Presence of Antibodies to AAV9 87
6.2.2.7 Risks Associated with Other Study Procedures 87
6.3 Safety Monitoring Committee 87
6.4 Pregnancy 87
6.5 Laboratory Analyses 88
6.6 Antibody/Biomarker Evaluation in Blood 91
6.7 Biomarker Evaluation in Urine 91
6.8 Cerebrospinal Fluid Evaluations 91
6.9 Sampling for Future Biomedical Research 92
6.10 Vital Signs 92
6.11 Electrocardiograms 92
6.12 Physical Examinations 92
6.13 Height, Weight, Body Mass Index, and Waist Circumference 92
6.14 Neurological Examinations 93

6.15 Magnetic Resonance Imaging and Magnetic Resonance Angiography.................93
6.16 Dual-Energy X-Ray Absorptiometry 93
6.17 Columbia Suicide Severity Rating Scale 93
6.18 Treatment-Induced Peripheral Neuropathy Assessment Scale 94
7 Statistical and Analytical Plan 95
7.1 Primary Endpoints 95
7.1.1 Primary Safety Endpoints 95
7.1.2 Primary Efficacy Endpoints 96
7.2 Secondary Endpoints 96
7.2.1 Secondary Efficacy Endpoints 96
[redacted]
7.4 Sample Size Calculations...98
7.5 Analysis Sets 98
7.6 Description of Subgroups to be Analyzed 98
7.7 Statistical Analysis Methodology 98
7.7.1 Analysis of Primary Endpoints 99
7.7.2 Analysis of Secondary Endpoints 99
7.7.2.1 Analysis of Secondary Efficacy Endpoints 99
7.7.2.2 Analysis of Secondary Safety Endpoints...99
7.7.3 Analyses of Exploratory Endpoints 99
7.7.3.1 Exploratory Efficacy Endpoints (Baseline to Year 1)...............................99
7.7.3.2 Exploratory Efficacy and Safety Endpoints (Post-Year 1) 99
7.7.4 Other Analyses...100
7.7.5 Interim Analysis...100
8 Data Quality Assurance 101
8.1 Data Management 101
9 Ethics 102
9.1 Independent Ethics Committee or Institutional Review Board 102
9.2 Ethical Conduct of the Study 102
9.3 Patient Information and Consent 102

9.4 Consent for Sampling for Future Biomedical Research 103
9.5 Patient Card....................................104
10 Investigator's Obligations 105
10.1 Confidentiality 105
10.2 Financial Disclosure and Obligations 105
10.3 Investigator Documentation....................................106
10.4 Study Conduct....................................106
10.5 Adherence to Protocol 106
10.6 Adverse Events and Study Report Requirements 107
10.7 Investigator's Final Report 107
10.8 Records Retention....................................107
10.9 Publications....................................107
11 Study Management....................................108
11.1 Monitoring 108
11.1.1 Independent Data Monitoring Committee 108
11.1.2 Monitoring of the Study....................................109
11.1.3 Inspection of Records 109
11.2 Management of Protocol Amendments and Deviations....................................110
11.2.1 Modification of the Protocol....................................110
11.2.2 Protocol Deviations 110
11.3 Study Termination....................................111
11.4 Final Report 111
12 Reference List....................................112
13 Appendices 119
13.1 Appendix: Schedule of Events....................................119
13.2 Appendix: Recommended Sequence of Study Assessments 131

List of Tables

Table 13-1 Schedule of Events – Main Study Period 120

Table 13-2 Schedule of Events – Follow-Up Period 128

Protocol Synopsis

**Protocol Number:**	PRV-FTD101
**Title:**	A Phase 1/2 Ascending Dose Study to Evaluate the Safety and Effects on Progranulin Levels of PR006A in Patients with Fronto-Temporal Dementia with Progranulin Mutations (FTD-GRN)
**Sponsor:**	Prevail Therapeutics, Inc.
**Study Phase:**	1/2
**Study Sites:**	This is a multi-center study conducted globally.
**Indication:**	Fronto-temporal dementia with progranulin mutations (FTD-GRN)
**Rationale:**	The purpose of this Phase 1/2 open-label study is to assess the safety, tolerability, immunogenicity, and effects of PR006A on progranulin protein (PGRN) levels in plasma and/or cerebrospinal fluid (CSF), as well as its immunogenicity and effects on biomarkers and efficacy parameters, in order to inform further clinical investigation and/or allow marketing registration of PR006A in a patient population suffering from FTD-GRN.
**Objectives:**	**Primary**<br>• Evaluate the safety, tolerability, and immunogenicity of 3 dose levels of PR006A administered via suboccipital injection into the cisterna magna<br>• Quantify PGRN levels in blood and CSF<br>**Secondary**<br>To evaluate the effect of PR006A on:<br>• Clinical Dementia Rating staging instrument plus National Alzheimer's Coordinating Center frontotemporal lobar degeneration domains (CDR® plus NACC FTLD)<br>• Neurofilament light chain (NfL) levels in blood and CSF<br>[redacted]

- [redacted]

[redacted]

Inclusion/Exclusion Criteria:

Each patient must meet all of the following criteria to be enrolled in this study:

1. Men or women aged 30 to 80 years (inclusive), at the time of informed consent.
2. Body weight range of ≥40 kg (88 lbs) to ≤110 kg (242 lb) and a body mass index (BMI) of 18 to 34 kg/m^2.
3. Has symptomatic FTD as per investigator assessment (behavioral-variant FTD [bvFTD], primary progressive aphasia [PPA]-FTD, FTD with corticobasal syndrome, or a combination of syndromes are allowed for enrollment).
4. Score ≥1 and ≤15 on CDR plus NACC FTLD sum of boxes (SB).
5. Stable use of background medications at least 8 weeks prior to investigational product dosing.

6. Carrier of a pathogenic* *GRN* mutation confirmed by the central laboratory.

   *All null mutations including nonsense, frameshift, splice site mutations, and complete or partial (exonic) gene deletions:

   - All previously published pathogenic mutations, with proven functional deleterious effect (selected missense mutations may be included provided they are known to be pathogenic)
   - All pathogenic mutations listed in Molgen FTD database (http://www.molgen.ua.ac.be)
   - All new mutations with low plasma PGRN level (<70 ng/mL) based on central laboratory measurement.
7. Negative screening test for *Mycobacterium tuberculosis* (MTB) or documented negative MTB test within 1 year prior to Screening.
8. Age- and gender-appropriate cancer screenings are up to date and completed as per the Investigator's judgment and local standard of care prior to Screening.
9. Patient and/or patient's legally authorized representative (LAR) (where applicable by local regulation) has the ability to understand the purpose and risks of the study and provide written informed consent and authorization to use protected health information in accordance with national and local privacy regulations. The patient or LAR may also provide consent for future biomedical research in accordance with their national regulations; however, the patient may still participate in the study without providing consent for future biomedical research.

10. Patient has a reliable study partner/informant (e.g., family member, friend) willing and able to participate in the study as a source of information on the patient's health status and cognitive and functional abilities (including providing input into the rating scales). The study partner should have regular contact with the patient (in person or via phone/video communication). The study partner must sign a separate partner informed consent form indicating that she/he understands the study requirements and is willing to participate and attend study visits requiring study partner input.
11. Women of nonchildbearing potential must be either surgically sterile (hysterectomy, bilateral tubal ligation, salpingectomy, and/or bilateral oophorectomy at least 26 weeks before Screening) or post-menopausal, defined as spontaneous amenorrhea for at least 2 years, with follicle-stimulating hormone level in the post-menopausal range at Screening based on the central laboratory's range.
12. Men and women of childbearing potential (i.e., ovulating, premenopausal, and not surgically sterile) must use a highly effective method of contraception consistently and correctly for the duration of the study including the long-term follow-up. Highly effective methods of contraception are those that, alone or in combination, result in a failure rate of less than 1% per year when used consistently and correctly (i.e., perfect use) and include the following for female participants of childbearing potential:
    a. Combined (estrogen and progestogen containing) oral, intravaginal, or transdermal hormonal contraception associated with inhibition of ovulation
    b. Oral, injectable, or implantable progestogen-only hormonal contraception associated with inhibition of ovulation
    c. Intrauterine device
    d. Intrauterine hormone-releasing system
    e. Bilateral tubal ligation or bilateral tubal occlusion (performed at least 3 months prior to Screening)
    f. Vasectomized partner (performed at least 3 months prior to Screening)

g. Sexual abstinence (no sexual intercourse)

Acceptable forms of contraception for male participants include:

a. Sexual abstinence (no sexual intercourse)

b. History of vasectomy (performed at least 3 months prior to Screening)

c. Condom with spermicide used together with highly effective female contraceptive methods if the female partner(s) is of childbearing potential (see above for list of acceptable female contraceptive methods)

13. Men must agree to abstain from sperm donation for the duration of the study, including long-term follow-up.
14. Women must agree to abstain from egg donation for the duration of the study, including long-term follow-up.
15. Women of childbearing potential cannot be pregnant or lactating/breastfeeding and must have a negative result for the serum pregnancy test (β-human chorionic gonadotropin) at Screening.
16. Patient is generally ambulatory and not dependent on a walker or wheelchair.
17. Patient is living in the community (i.e., not in a nursing home); some levels of assisted living may be permitted at the discretion of the Investigator.
18. Pneumococcal pneumonia and shingles vaccines are required within 10 years of screening (allowed to be performed during screening but must be given at least 4 weeks prior to initiation of immunosuppressant regimen).

Patients meeting any of the following criteria will be excluded from the study:

1. Diagnosis of a significant central nervous system (CNS) disease other than FTD that may be a cause for the patient's FTD symptoms or may confound study objectives.
2. Brain MRI/magnetic resonance angiography (MRA) imaging indicating clinically significant abnormality, including evidence of prior hemorrhage, infarct >1 cm^3 or >3 lacunar infarcts, or a structural or vascular abnormality deemed a contraindication to intracisternal injection.

3. Hypersensitivity or contraindications to corticosteroid and/or sirolimus use (including but not limited to osteoporosis with vertebral fractures within 1 year prior to Screening, poorly controlled diabetes [see Exclusion Criterion 5c], uncontrolled hypertension [see Exclusion Criterion 5f]), uncontrolled hyperlipidemia or hypercholesterolemia as per Investigator assessment, uncontrolled interstitial lung disease, or uncontrolled renal insufficiency).
4. Clinical evidence of peripheral symmetric sensory polyneuropathy (stable sensory mononeuropathies and radiculopathies are not exclusionary).
5. Concomitant disease or condition within 6 months of Screening that could interfere with, or treatment of which might interfere with, the conduct of the study or that would, in the opinion of the Investigator, pose an unacceptable safety risk to the patient or interfere with the patient's ability to comply with study procedures; including, but not limited to, the following:
    a. Evidence of clinically significant liver disease
    b. Unstable autoimmune disease requiring chronic immunosuppression
    c. Poorly controlled/not adequately managed diabetes (Screening hemoglobin A1c [HbA1c] ≥7%)
    d. History of unstable angina, myocardial infarction, chronic heart failure (New York Heart Association Class III or IV), or clinically significant conduction abnormalities (e.g., unstable atrial fibrillation) within 1 year prior to Screening
    e. Clinically significant 12-lead electrocardiogram (ECG) abnormalities at Screening, as determined by the Investigator
    f. Uncontrolled hypertension defined as: average of 3 systolic blood pressure [SBP]/diastolic blood pressure [DBP] readings >165/100 mm Hg at Screening, or persistent SBP/DBP readings >180/100 mm Hg within 3 months prior to Screening that, in the opinion of the Investigator, are indicative of chronic uncontrolled hypertension
    g. History of cancer within 5 years of Screening or current presence of pre-cancer lesions, with the

exception of fully excised nonmelanoma skin cancers and fully excised prostate carcinoma in situ that have been stable for at least 6 months

h. History or current alcohol or drug abuse within 2 years of Screening
i. Any current psychiatric diagnosis according to the Diagnostic and Statistical Manual of Mental Disorders Fifth Edition, International Statistical Classification of Diseases and Related Health Problems Tenth Revision, or equivalent, that may interfere with the patient's ability to perform study procedures and all assessments (e.g., psychosis, major depression, bipolar disorder, mental retardation, and schizophrenia). NOTE: Psychiatric manifestations of FTD are not exclusionary
j. At imminent risk of self-harm, based on clinical interview and responses on the Columbia Suicide Severity Rating Scale (C-SSRS). Patient must be excluded if they report ideation with intent, with or without a plan or method (i.e., positive response to item 4 or 5 on the C-SSRS) in the past 2 months or suicidal behavior in the past 6 months
k. Any medical disorders that, in the opinion of the Investigator, could interfere with study-related procedures (including safe performance of lumbar puncture or intracisternal injection), such as prohibitive spinal diseases, bleeding diathesis, clinically significant coagulopathy, thrombocytopenia, or increased intracranial pressure
l. Documented stroke or transient ischemic attack within 1 year prior to Screening
m. History of seizure or unexplained blackouts, with the exception of seizure due to known, transient cause (e.g., medication, electrolyte disturbance), within 10 years prior to Screening
n. Currently active infection or severe infection (e.g., pneumonia, septicemia, CNS infections [e.g., meningitis, encephalitis]) within 12 weeks prior to Screening
o. History of severe allergic or anaphylactic reactions. History of hypersensitivity to any inactive ingredient

of the investigational product (refer to the investigator's brochure) or protocol-required immunosuppressant medications

p. Clinical evidence of vitamin B_{12} deficiency or vitamin B_{12} level less than the lower limit of normal if deemed clinically significant as per Investigator's assessment at Screening
q. History of neurosyphilis or history of syphilis infection without documentation of adequate treatment.
r. Subject is generally frail or has any medical condition, for which in the view of the investigator, participation in the study would not be in the best interest of the subject or is likely to prohibit further participation during the study period

6. Clinically significant abnormalities in laboratory test results at Screening as given below (laboratory testing may be repeated with medical monitor approval):
   a. Total bilirubin, alanine aminotransferase, or aspartate aminotransferase >1.5 × the upper limit of normal (ULN) (note: patients with confirmed Gilbert syndrome are allowed for enrollment with Sponsor's agreement)
   b. Serum creatinine >1.5 × ULN
   c. Hematocrit <35% for men and <32% for women
   d. Absolute neutrophil count <1500/µL
   e. Platelet count <100 000/µL
   f. International normalized ratio >1.4, or other coagulopathy
   g. Activated partial thromboplastin time >50 seconds
   h. Thyrotropin level outside the normal range and deemed clinically significant by the Investigator
   i. Positive result for hepatitis B surface antigen, hepatitis C antibody, or human immunodeficiency virus 1 or 2.
   j. Any other abnormal Screening laboratory test result deemed clinically significant by the Investigator.
7. Participation within 3 months prior to Screening in another therapeutic investigational drug or device study with purported disease-modifying effects on FTD, unless

it can be documented that the patient received placebo only.

8. Any type of prior gene or cell therapy.
9. Immunizations (live vaccines) in the 4 weeks prior to Screening. Note: Pneumococcal vaccine and shingles vaccine administration is allowed during the Screening Period (patients not previously vaccinated should receive pneumococcal and/or shingles vaccine administration at least 4 weeks prior to sirolimus loading dose).
10. Use of blood thinners (e.g., warfarin, heparin, and novel oral anticoagulants) in the 2 weeks prior to Screening or the anticipated need to initiate blood thinners during the study. Antiplatelet therapies (prophylactic aspirin, clopidogrel) are acceptable if the patient is medically able to temporarily stop from at least 7 days prior to and at least 48 hours after intracisternal injection and lumbar puncture.
11. Contraindications or intolerance to imaging methods (MRI, computed tomography [CT]) inducing claustrophobia and intolerance to contrast agents used for MRI or CT (including but not limited to gadolinium contrast agents and iohexol).
12. Contraindications to general anesthesia or deep sedation.
13. Positive urine test for drugs of abuse (including opiates, amphetamines, cocaine, barbiturates, and phencyclidine) without prescription at Screening and Day -1. Note: Use of medical marijuana is permitted provided that the patient is on a stable regimen. It is also permitted if the patient resides in a state in which the recreational use of marijuana is legalized, so long as the patient does not meet drug abuse criteria (as defined in the Diagnostic and Statistical Manual of Mental Disorders, Fifth Edition).

Study Design:

Study PRV-FTD101 is a Phase 1/2, multi-center, open-label ascending dose, first-in-human study that will evaluate the safety and effect on PGRN levels of intracisternal PR006A administration in patients with FTD-GRN. [redacted] escalating-dose cohorts are planned (3.5 × 10^{13} vg, 7.0 × 10^{13} [redacted] of PR006A).

This is a 5-year study. During the first year, patients will be evaluated for the effect of PR006A on safety, tolerability,

immunogenicity, biomarkers, and efficacy. Patients will follow up for an additional 4 years to continue to monitor safety, selected biomarkers, and efficacy parameters.

To minimize patient burden due to travel for evaluations, and in certain circumstances where the study site is unable to perform the PR006A administration procedure, patients will be permitted to be dosed at one study site and complete follow-up visits after dosing at a separate study site close to their home, with Sponsor's approval. The study site where follow-up evaluations will be performed must be an active site in the PRV-FTD101 study.

Fifteen patients will be administered a one-time dose of PR006A, suboccipitally injected into the cisterna magna by an interventional radiologist or neurosurgeon.

In each cohort, enrollment will be staggered by at least 8 weeks between the first 2 patients and then an additional 8 weeks between the second patient and the remaining 3 patients of the cohort to permit review of safety, tolerability, immunogenicity, and, to the extent available, efficacy by the iDMC, who will ultimately provide a recommendation on further dosing. At least 8 weeks after the second staggered patient is dosed in Cohort 1, the iDMC will review all available safety, tolerability, immunogenicity, and, to the extent feasible, efficacy results from the first 2 patients in this cohort (dosed in a staggered manner). At the recommendation of the iDMC, the Sponsor will decide whether to dose the remaining 3 patients in Cohort 1 (low dose) without staggering and in parallel begin staggered enrollment in the mid-dose cohort (Cohort 2).

Cohort 2 will enroll 5 patients to receive the mid-dose (7.0×10^{13} vg) of PR006A, [redacted] of PR006A. The design for Cohorts 2 [redacted] enrollment is the same as for Cohort 1.

If a decision is made to not open or to limit enrollment into a cohort (i.e., Cohort 2 [redacted] 3) due to safety or tolerability reasons, the unenrolled subjects may be re-allocated to a lower dose level cohort.

One loading dose of 6 mg oral sirolimus will be administered at Day -1, as well as a single dose of 1 g IV methylprednisone at Day 0. A concomitant maintenance dose of 2 mg/day sirolimus will be administered orally for 90 days starting at Day 0 which will then be tapered over the ensuing 15 to 30 days. The maintenance dose is administered to achieve a trough sirolimus

concentration of 4 ng/mL (range 2 to 8 ng/mL). One day after treatment with PR006A, patients will start prednisone 30 mg orally for 14 days, followed by a 7-day taper. At the Investigator's discretion, adjustments of dose and duration are permitted.

An iDMC consisting of clinicians and a biostatistician otherwise unaffiliated with the conduct of the study will conduct a review of the safety, tolerability, immunogenicity; and to the extent available, efficacy data in accordance with the charter. Safety will be assessed by vital signs; physical and neurological examinations; safety laboratory tests; immunogenicity; ECGs; C-SSRS; Treatment-Induced Neuropathy Assessment Scale (TNAS); safety MRI data; treatment-emergent changes in body weight, BMI, and waist circumference; treatment-emergent hyperlipidemia/hypercholesterolemia; treatment-emergent interstitial lung disease; treatment-emergent proteinuria; incidence of opportunistic infections; sirolimus trough concentrations; treatment-emergent adverse events (TEAEs); and adverse events (AEs).

The Sponsor will perform 3 formal interim analyses: 1) after all patients complete 12 months of treatment in Cohort 1; 2) after all patients complete 12 months of treatment in Cohort 2; [redacted]

Additionally, the Sponsor will perform a review of biomarker data at regular intervals. Based on the review of data through Month 2 in at least 2 patients and in consultation with the iDMC, a decision will be made to continue or suspend enrollment in any enrolling cohorts and to start enrollment in the subsequent cohort (i.e., dose escalate). A decision to dose escalate will be based on data from at least 2 patients from the concurrent cohort. However, all cohorts will enroll a minimum of 3 patients, unless enrollment is terminated due to safety concerns or based on biomarker review this dose is considered to be non-efficacious. In all other circumstances, the full cohort of 5 patients will be enrolled.

Post-Year 1

Patients will have bi-annual follow-up site visits to assess the effect of long-term exposure to PR006A on safety, selected biomarker, and efficacy outcomes.

**Estimated Study Duration:**	Patients completing the study will be enrolled for a period of approximately 5 years.
**Efficacy Assessments:**	The efficacy of PR006A will be evaluated by changes in the following:<br>• PGRN levels in blood and CSF<br>• CDR plus NACC FTLD<br>• NfL levels in blood and CSF<br>• Montreal Cognitive Assessment (MoCA)<br>• Benson Complex Figure Copy and Recall<br>• Clinical Global Impressions – Severity/Improvement (CGI-S/I) scale<br>• Trail Making Test, Part A (TMT-A) and Part B (TMT-B)<br>• Digit Span Test (forwards and backwards)<br>• Category Fluency<br>• Multilingual Naming Test (MINT)<br>• [redacted]<br>• [redacted]
**Safety Assessments:**	Safety variables will include AEs (including AEs leading to withdrawal and Grade 3 or higher and all serious AEs [SAEs]); adverse reactions [ARs] (including serious and suspected); vital signs; clinical laboratory assessments (including blood chemistry, hematology, coagulation, urinalysis); adeno-associated virus serotype 9 (AAV9) enzyme-linked immunospot in blood; anti-AAV9 antibodies in CSF and blood; cell count, protein, and glucose in CSF; C-SSRS; TNAS; treatment-emergent transaminitis; treatment-emergent diabetes and prediabetes; treatment-emergent peptic ulcer disease and/or erosive gastritis; treatment-emergent osteoporosis; treatment-emergent increase in body weight ≥5% from baseline, treatment-emergent BMI >25 or >30, and treatment-emergent increase in waist circumference ≥15% from baseline; incidence of Grade ≥3 infections; incidence/aggravation of psychosis; treatment-emergent hypercholesterolemia or hyperlipidemia; treatment-emergent interstitial lung disease; treatment-emergent opportunistic infections (including progressive multifocal leukoencephalopathy (PML) and BK virus-associated

	nephropathy); sirolimus trough concentrations; 12-lead ECGs; brain MRIs; physical examinations; and neurological examinations.<br>Viral shedding, i.e., excretion/secretion of viral particles that could be transmitted to other individuals, will be assessed in saliva, urine, and stool samples.<br>Dosing will be suspended if study stopping rules are met.
**Investigational Product, Dosage, and Route of Administration:**	PR006A is an investigational AAV9 gene therapy drug product.<br>PR006A concentrate for the low, mid,[redacted] dose will be supplied as frozen solution for injection in single-use polypropylene vials or Type I, Class A glass serum vials and stored in individually labeled vials at -60°C or lower.<br>[redacted] escalating-dose cohorts are planned (3.5×10^{13} vg, 7.0×10^{13} vg,[redacted] of PR006A).<br>PR006A will be administered as a single dose via suboccipital injection into the cisterna magna (intracisternal magna). The procedure will be performed with the patient under general anesthesia or deep sedation and using imaging guidance.
**Sample Size:**	Fifteen patients ([redacted] cohorts of 5 patients each) will be administered a 1-time dose of PR006A (low, mid,[redacted] dose). The sample size is based on enrollment feasibility and clinical objectives rather than statistical rationale. The sample size of 5 patients for the low-, mid-, [redacted]-dose cohorts is considered to be sufficient for evaluation of the safety, tolerability, and immunogenicity of PR006A in each cohort. The sample size is not based on statistical power considerations.
**Statistical Methods:**	The following analysis sets will be used in the statistical analyses:<br>• Safety Analysis Set, which will include all enrolled patients who receive investigational product.<br>• Treatment Analysis Set, which will include all patients in the Safety Analysis Set and who have at least 1 efficacy evaluation following the Baseline visit.<br>Patient information, safety, efficacy, and biomarker results will be summarized descriptively. No inferential analysis will be performed.

Primary Endpoints:

The primary endpoints will be summarized with descriptive statistics, at all time points where these variables are collected. No inferential statistical tests will be performed using safety data. The Safety Analysis Set will be used for analyses of the safety endpoints. The Treatment Analysis Set will be used for analyses of the efficacy endpoints.

The following primary safety endpoints are measured up to 5 years:

- Incidence and severity of treatment-emergent AEs and SAEs, including clinically significant changes in vital signs, clinical laboratory assessments, immunogenicity, C-SSRS, TNAS; treatment-emergent transaminitis; treatment-emergent diabetes and prediabetes; treatment-emergent peptic ulcer disease and/or erosive gastritis; treatment-emergent osteoporosis; treatment-emergent increase in body weight ≥5% from baseline, treatment-emergent BMI >25 or >30, and treatment-emergent increase in waist circumference ≥15% from baseline; incidence of Grade ≥3 infections; incidence/aggravation of psychosis; treatment-emergent hypercholesterolemia or hyperlipidemia; treatment-emergent interstitial lung disease; treatment-emergent opportunistic infections (including progressive multifocal leukoencephalopathy (PML) and BK virus-associated nephropathy); sirolimus trough levels; 12-lead ECGs; physical examinations; and neurological examinations
- Sum of ARs and suspected ARs
- Sum of serious ARs and serious suspected ARs
- Incidence of procedure or treatment-emergent safety findings as per brain MRI
- Change from baseline in immunogenicity of AAV9 and PGRN in blood at Day 14 and Months 1, 2, 3, 6, 9, and 12 and in CSF at Months 2 and 12

The following are the primary efficacy endpoints:

- Change from baseline in PGRN levels in blood at Months 1, 2, 3, 6, 9, and 12
- Change from baseline in PGRN levels in CSF at Months 2 and 12

Secondary Endpoints:

The following secondary endpoints, consisting of efficacy and safety measures will be measured from baseline to Year 1 and will be summarized by assessment time. Corresponding changes from baseline will also be summarized for these measures. The Treatment Analysis Set will be used for analyses of the secondary efficacy endpoints. The Safety Analysis Set will be used for analyses of the secondary safety endpoints.

Secondary Efficacy Endpoints

- Change from baseline in CDR plus NACC FTLD at Months 6 and 12
- Change from baseline in NfL levels in blood at Months 2, 6, 9, and 12
- Change from baseline in NfL levels in CSF at Months 2 and 12

[redacted]

- [redacted]
- [redacted]
- [redacted]
- [redacted]
- [redacted]
- [redacted]
- [redacted]
- [redacted]

[redacted]

**Interim Analysis:**	To facilitate the ongoing review of efficacy biomarker data (e.g., PGRN in blood and CSF), informal interim analyses may be performed at regular intervals based on Sponsor's discretion.<br>The Sponsor will perform 3 formal interim analyses: 1) after all patients complete 12 months of treatment in Cohort 1; 2) after all patients complete 12 months of treatment in Cohort 2; [redacted].<br>The statistical analysis plan and all patient data for each cohort will be finalized prior to each formal interim analysis. Additional interim analyses may be performed during the follow-up period (post-Year 1) based on Sponsor's discretion.

**Version and Date of Protocol:**	Version 4.0; 15 July 2020

List of Abbreviations

Abbreviation	Definition
AASLD	American Association for the Study of Liver Diseases
AAV	adeno-associated virus
AAV9	adeno-associated virus serotype 9
AE	adverse event
ALT	alanine aminotransferase
AR	adverse reaction
AST	aspartate aminotransferase
BMI	body mass index
BMP	Bis(monoacylglycero)phosphate
bvFTD	behavioral-variant fronto-temporal dementia
CBA	chicken β-actin
CBD	corticobasal degeneration
CDR® plus NACC FTLD	Clinical Dementia Rating staging instrument plus National Alzheimer’s Coordinating Center frontotemporal lobar degeneration domains
CDROM	compact disc, read-only memory
CFR	Code of Federal Regulations
CGI-I	Clinical Global Impressions-Improvement
CGI-S	Clinical Global Impressions-Severity
CLIA	Clinical Laboratory Improvement Amendments
CMVe	cytomegalovirus enhancer
CNS	central nervous system
COVID-19	coronavirus disease 2019
CRO	contract research organization
CSF	cerebrospinal fluid
C-SSRS	Columbia Suicide Severity Rating Scale
CT	computed tomography
CTA	clinical trial agreement
CTCAE	Common Terminology Criteria for Adverse Events
DBP	diastolic blood pressure
DEXA	dual-energy x-ray absorptiometry

**Abbreviation**	**Definition**
DRG	dorsal root ganglia
ECG	electrocardiogram
eCRF	electronic case report form
EDC	electronic data capture
ELISpot	enzyme-linked immunospot
FDA	Food and Drug Administration
FTD	fronto-temporal dementia
FTD-FUS	fronto-temporal dementia characterized by TDP-negative inclusions that contain the fused-in-sarcoma protein
FTD-GRN	fronto-temporal dementia with progranulin mutations
FTD-TDP	fronto-temporal dementia containing TDP-43
GCP	Good Clinical Practice
GFAP	glial fibrillary acidic protein
GLP	Good Laboratory Practice
GRN	progranulin gene
HbA1c	hemoglobin A1c
HIV	human immunodeficiency virus
IB	investigator's brochure
ICF	informed consent form
ICH	International Council for Harmonisation
ICM	intracisternal magna
ICV	intracerebroventricular
iDMC	independent data monitoring committee
IEC	independent ethics committee
iPSC	induced pluripotent stem cell
IRB	institutional review board
IV	Intravenous(ly)
KO	knockout
LAR	legally authorized representative
LP	lumbar puncture
MedDRA	Medical Dictionary for Regulatory Activities
MINT	Multilingual Naming Test

Abbreviation	Definition
MoCA	Montreal Cognitive Assessment
MRA	magnetic resonance angiography
mRNA	messenger ribonucleic acid
MRI	magnetic resonance imaging
MTB	*Mycobacterium tuberculosis*
NCL	neuronal ceroid lipofuscinosis
[redacted]	[redacted]
NHP	nonhuman primate
PGRN	progranulin protein
PK	pharmacokinetic
PPA	primary progressive aphasia
PPA-FTD	primary progressive aphasia fronto-temporal dementia
qPCR	quantitative polymerase chain reaction
SAP	statistical analysis plan
SB	sum of boxes
SBP	systolic blood pressure
SUSAR	suspected unexpected serious adverse reaction
TDP-43	TAR-DNA-binding protein 43 kDa
TEAE	treatment-emergent adverse event
TIPN	treatment-induced peripheral neuropathy
TNAS	Treatment-Induced Neuropathy Assessment Scale
TMT-A	Trail Making Test, Part A
TMT-B	Trail Making Test, Part B
vMRI	volumetric magnetic resonance imaging
WHODrug	World Health Organization Drug Dictionary

1 Introduction

1.1 Background of Disease

Fronto-temporal dementia (FTD) is a devastating dementia syndrome encompassing a heterogenous group of clinical syndromes characterized by progressive deficits in behavior, executive function, and language (Bang et al 2015; Young et al 2018). Its estimated prevalence is 15 per 100 000 patients between 45 and 65 years of age (Johnson et al 2005; Knopman and Roberts 2011; Onyike and Diehl-Schmid 2013), although as many as 10% of FTD patients are younger than 45 years (Knopman and Roberts 2011). Progression is typically more aggressive than Alzheimer's Disease with death occurring within 3 to 10 years after diagnosis (Nunnemann et al 2011; Hodges et al 2003). The main causes of death arise as complications of immobility among bedridden patients (e.g., pneumonia, circulatory system failure, cachexia) (Nunnemann et al 2011). No disease-modifying therapies are available for patients with FTD.

Clinically, FTD is usually categorized into 3 variants distinguished by their presenting symptoms and regional pattern of atrophy (Gorno-Tempini et al 2011; Kertesz et al 1999; Kertesz et al 2005; Neary et al 1998). One variant is behavioral-variant FTD (bvFTD), which is characterized by personality changes, disinhibition, and apathy, accompanied by degeneration of the frontal lobes. A second variant is primary progressive aphasia (PPA, or PPA-FTD), which is characterized by phonological disorders/language difficulties and specific patterns of temporal lobe atrophy. There are 2 main subtypes of PPA-FTD: nonfluent variant PPA, characterized by slow speech production, misuse of grammar, multimodal agnosia, and atrophy of the left inferior frontal and insular lobes, and semantic-variant PPA, characterized by semantic aphasia, impaired word comprehension, and atrophy of anterior and inferior temporal lobes that ultimately spreads to other parts of the brain, including the orbitofrontal cortex. A logopenic-variant PPA has also been described (Gorno-Tempini et al 2008; Teichmann et al 2013). A third variant is an overlap syndrome in which FTD is associated with parkinsonism or motor neuron disease resembling amyotrophic lateral sclerosis (Mackenzie and Rademakers 2007; Tolnay and Probst 2002) and may also include such presentations as corticobasal degeneration (CBD) and progressive supranuclear palsy.

There are 3 major pathological categories of FTD: FTD-tau representing approximately 40% of FTD cases and characterized by the presence of hyperphosphorylated tau aggregates; FTD-TDP representing approximately 50% cases and characterized by tau- and

α-synuclein-negative cytoplasmatic inclusions containing TDP-43 and ubiquitin; and FTD-FUS representing 10% to 20% of FTD cases, characterized by TDP-negative inclusions that contain the fused-in-sarcoma protein (FUS) (Mackenzie and Neumann 2012) and possibly other related proteins (e.g., EWSR1, TAF15) (Nunnemann et al 2011). Less common pathologies include neuronal intermediate filament inclusion disease, or no detectable inclusions (Kertesz et al 2005; Cairns et al 2007).

Up to 40% of FTD cases are familial, and about one-third of those cases are caused by mutations in the progranulin gene (*GRN*), tau (*MAPT*), or chromosome 9 open reading frame 72 (*C9orf72*). Overall, *GRN* mutations account for about 5% to 10% of all patients with FTD and approximately 22% of familial FTD cases (Baker et al 2006; Cruts et al 2006; Gass et al 2006).

FTD-GRN patients carry a single mutation in the *GRN* gene, which encodes the progranulin protein (PGRN), resulting in haploinsufficiency and an approximately 50% reduction in PGRN levels. *GRN* mutation carriers have an approximately 90% risk of developing FTD by age 75 (Baker et al 2006; Cruts et al 2006; Gass et al 2006). Low PGRN levels in blood or cerebrospinal fluid (CSF) predict the presence of a GRN mutation even in the asymptomatic stage of the disease (Galimberti et al 2018, Ghidoni et al 2012) but does not correlate with disease state or progression (Galimberti et al 2018, Guven et al 2019). Clinically, FTD-GRN patients can develop any of the known variants, but most commonly develop bvFTD (Carrasquillo et al 2010). FTD-GRN pathology is characterized by aggregates of ubiquitin and TDP-43 proteins in the brain, which are believed to be toxic (Chang et al 2017). Concurrently, TDP-43, which is typically a nuclear protein, is relatively depleted from the nucleus due to aggregation in the cytoplasm. Inflammatory changes, including abnormal microglial activation and the release of cytotoxic cytokines and inflammatory complement pathway factors, are also seen in the in the brain of FTD-GRN patients and in animal models of PGRN insufficiency (Arrant et al 2018; Kao et al 2017; Valdez et al 2017; Ward et al 2017; Lui et al 2016).

Progranulin is a secreted glycoprotein broadly expressed in the CNS and periphery in a variety of cells and has been implicated in several physiological functions and roles including as an activator of lysosome function, an anti-inflammatory, a neurotrophic factor, and a growth factor. The mechanisms by which PGRN deficiency results in FTD is not fully elucidated, but likely relates to CNS lysosome dysfunction and inflammation. Progranulin

deficiency leads to age-dependent lysosomal dysfunction, as manifested by ineffective protein degradation and recycling in animal and cell models of PGRN deficiency (Arrant et al 2018; Kao et al 2017; Valdez et al 2017; Evers et al 2017). Furthermore, PGRN regulates the maturation and processing of key lysosome enzymes, such as the cathepsin D protease, which are defective in the context of PGRN deficiency. By regulating processing, PGRN regulates the activity of cathepsin D protease both in mice (Zhou et al 2017) and in FTD-GRN patients (Valdez et al 2017; Ward et al 2017). Progranulin deficiency in model systems also leads to neuroinflammation (Arrant et al 2018; Kao et al 2017; Valdez et al 2017; Ward et al 2017; Lui et al 2016), which is strongly implicated in FTD patient pathology.

Current standard of care for FTD involves off-label use of existing drugs, and only modestly addresses disease symptoms. For instance, antipsychotics (risperidone, olanzapine, and aripiprazole) are often used to treat behavioral symptoms, although they may have extrapyramidal side effects and lead to increased mortality. Selective Serotonin Reuptake Inhibitors are sometimes utilized to address behavioral symptoms. Other drugs, including cholinesterase inhibitors and memantine (an inhibitor of the *N*-methyl-*D*-aspartate glutamate inhibitor), have not been shown to improve symptoms, but nevertheless are often prescribed to FTD patients. Recent studies of the calcium channel blocker nimodipine failed to show an effect in FTD-GRN (Sha et al 2017). There are currently no US Food and Drug Administration (FDA)-approved disease-modifying therapies indicated for FTD.

1.1.1 Background on PR006A

PR006A is an investigational gene therapy being developed as a disease-modifying, one-time treatment for FTD-GRN. PR006A utilizes an adeno-associated virus serotype 9 (AAV9) viral vector to deliver a functional copy of the wildtype *GRN* gene, which encodes the wildtype PGRN, to a patient’s cells. PR006A contains elements to constitutively express *GRN* under the control of the cytomegalovirus enhancer (CMVe) and chicken β-actin (CBA) promoter using a codon-optimized coding sequence for human *GRN*. The vector shares most features of other AAV9 vectors that have been used safely in clinical studies, including clinical studies for spinal muscular atrophy type 1 (Mendell et al 2017).

The AAV9 vector is particularly well suited for gene therapy of CNS disorders given its ability to transduce multiple CNS cell types, the persistence of expression of the transgene, and its safety record (Samulski and Muzyczka 2014; Weinberg et al 2013). As multiple brain

regions are affected in FTD-GRN, broad biodistribution of PR006A is needed. Based on NHP research, broad brain distribution is best achieved by injecting investigational product (i.e., PR006A) into the cisterna magna (Hinderer et al 2018; Zerah et al 2015; Gray et al 2013). However, localized intracerebral adeno-associated virus (AAV)-GRN gene therapy has been reported to correct disease-related phenotypes in *Grn* knockout (KO) mice (Arrant et al 2018), demonstrating that it is possible to express active PGRN in the brain to restore function through cross-correction.

Finally, while the precise role of PGRN in healthy and diseased brains is only partially understood, the gene therapy approach does not rely on a detailed mechanistic understanding of PGRN function or the multiple biological pathways it affects. Instead, the rationale for this gene therapy approach relies on the well-established knowledge that patients who carry a single copy of a mutated *GRN* gene have substantially reduced levels of PGRN and a 90% likelihood of developing FTD. Replenishing PGRN levels both intra- and extra-cellularly in the brain by broadly delivering copies of the functional wildtype *GRN* gene is predicted to provide clinical benefit via all PGRN-related mechanisms relevant for the disease process.

1.1.2 Nonclinical Overview

PR006A efficacy was evaluated in 2 models of FTD-GRN: (1) an in vitro induced pluripotent stem cell (iPSC)-derived neuronal culture (iPSC-neuron) model, which uses cells from patients carrying heterozygous *GRN* mutations, and (2) an in vivo *Grn* KO genetic mouse model. These models were chosen as they recapitulate the underlying PGRN deficiency observed in patients with FTD-GRN. The iPSC-neuron model expresses approximately one-half of normal PGRN levels and has impaired lysosomal function. The *Grn* KO mice completely lack PGRN and exhibit lysosomal and neuroinflammatory phenotypes.

In vivo studies were conducted in the *Grn* KO mouse model, which is the most frequently used animal model in preclinical studies of FTD-GRN and which have a complete loss of PGRN expression. While the most accurate mouse genetic model approximation to patients with FTD-GRN vis-à-vis PGRN levels would be mice with a heterozygous loss-of-function *Grn* mutation, such mice fail to demonstrate a phenotype that is consistent with the human disease. In contrast, *Grn* KO mice display age-dependent phenotypes, including lysosomal alterations, neuronal lipofuscin accumulation, ubiquitin accumulation, microgliosis, and neuroinflammation, recapitulating many key pathological features of human FTD-GRN. Three nonclinical in vivo efficacy studies were performed: 2 single-dose studies in aged

Grn KO mice and a dose-ranging study in adult *Grn* KO mice. PR006A was administered via intracerebroventricular (ICV) injection, as ICM injection (the intended clinical route of administration) is technically difficult in mice. In-life safety assessments and post-mortem histopathology by a blinded board-certified pathologist were also performed in these experiments.

In the single-dose studies in 14- to 16-month-old aged *Grn* KO mice (PRV-2018-027, PRV-2019-002), PR006A was delivered by ICV injection at a dose of 2.4×10^{11} vg/g brain, and the animals were sacrificed 2 months post-injection. PR006A resulted in broad transgene biodistribution and PR006A-encoded PGRN expression throughout the CNS (i.e., cerebral cortex and spinal cord) and peripheral tissues. PR006A reduced key FTD-GRN-related phenotypes in the brain of *Grn* KO mice, including accumulation of lipofuscin and ubiquitin, markers indicative of lysosomal abnormalities, and proinflammatory cytokine expression and microgliosis, markers indicative of chronic CNS inflammation. Post-mortem histopathology on all major organs revealed no adverse PR006A-related findings.

In a dose-ranging study (PRV-2019-004), 4-month-old adult *Grn* KO mice were delivered PR006A by ICV injection at a low dose (2.7×10^{9} vg/g brain), mid dose (2.7×10^{10} vg/g brain), or high dose (2.7×10^{11} vg/g brain), and the animals were sacrificed 3 months post-injection. PR006A effectively transduced *Grn* KO mice, resulting in a dose-dependent biodistribution of the transgene. PR006A treatment also led to production of progranulin mRNA and PGRN in the CNS. A dose-response relationship between PR006A and decreased lipofuscinosis was observed throughout multiple regions of the brain. A statistically significant reduction of lipofuscinosis was observed at the mid and high doses of PR006A. PR006A decreased ubiquitin accumulation throughout multiple brain regions at all doses. A reduction in gene expression of proinflammatory cytokine and microgliosis markers to wildtype levels was observed in the cerebral cortex at all doses of PR006A. In the brain, PR006A also decreased protein expression of microgliosis markers at all doses and astrocytosis markers at the high dose. Post-mortem histopathology on all major organs showed no adverse PR006A-related findings.

The in vitro human neuronal model of progranulin haploinsufficiency was used since it is an excellent tool to assess FTD-GRN-related phenotypes such as TDP-43 pathology that do not present in the in vivo *Grn* KO mouse model. iPSC lines from 2 independent heterozygous *GRN* mutation carrier patients and from a healthy-aged subject with no *GRN* mutation were

differentiated into neuronal cultures for efficacy experiments. The level of PGRN in FTD-GRN iPSC-derived cell lines was approximately 25% to 50% of control cells, consistent with replicating the effect of progranulin haploinsufficiency on plasma PGRN levels in FTD-GRN patients. PR006A effectively transduced human cells in vitro, resulting in a dose-dependent production of PGRN. PR006A ameliorated TDP-43 pathology in the FTD-GRN iPSC-neurons. In addition, PR006A reversed deficits in the maturation of the lysosomal enzyme cathepsin D, a necessary step for normal lysosomal function.

In addition to the mouse studies described above, safety and biodistribution of PR006A were evaluated in 2 toxicology studies using cynomolgus macaques. The brain of the NHP is most similar to that of humans, and the anatomical features of the NHP spinal cord and CSF volume and flow permits an ICM injection, thus mimicking the route of administration intended for clinical dosing. Because of the anatomical similarities to humans, it is expected that NHP studies will provide more reliable biodistribution data supporting clinical dosing of PR006A.

A pilot, non-GLP (Good Laboratory Practice) NHP study (PRV-2018-021) using PR006A research-grade material was conducted to confirm that PR006A is well tolerated following ICM administration. Animals were treated with either 4.9×10^{12} vg of PR006A (6.6×10^{10} vg/g brain, assuming a brain weight of 74 g) or excipient alone. The study duration was 4 weeks post-injection, the anticipated peak expression time point. There were no unscheduled deaths, and all monkeys survived until the scheduled necropsy. No differences were noted in the functional observation battery, food consumption, or weight gain throughout the duration of the study between the control monkeys that received excipient and monkeys that were treated with PR006A.

Tissue samples were collected from the brain and peripheral organs for histopathology. Tissues were processed and stained with hematoxylin and eosin for evaluation. In animals injected with PR006A, meningeal and perivascular mononuclear inflammatory cell infiltrates were observed throughout the brain. This finding was consistently minimal in severity. The results were considered inconclusive, since this was a small (n=2/group) pilot study.

In the 6-month, multidose GLP NHP toxicology study (PRV-2018-028; n=6-7/dosing group), male and female cynomolgus monkeys 2 to 4 years of age were treated with PR006A administered once via ICM injection with a 7-day, 30-day, or 183-day post-administration

observation period. The study evaluated 2 dose levels: the highest dose (6.5×10^{10} vg/g brain) was the maximum feasible dose achievable at the time of the experiment with 1.2 mL volume of undiluted test product, and the low dose (6.5×10^{9} vg/g brain) is equivalent to one log lower than the high dose. The study also included a control arm in which animals received 1.2 mL of excipient only (20 mM Tris pH 8.0, 200 mM NaCl, 1 mM MgCl2, and 0.001% poloxamer 188). No adverse PR006A-related clinical observations, body weight changes, ophthalmic observations, or physical or neurological examination findings were noted. No in-life, clinical pathology, or anatomic pathology observations were observed that were considered related to PR006A.

No PR006A-related microscopic findings were observed in Day 7 or Day 30 animals. At the terminal sacrifice at Day 183, PR006A-related microscopic findings were observed in the dorsal funiculus of the cervical, thoracic, and (to a lesser extent) the lumbar spinal cord and the dorsal root ganglia (DRG).

Animals administered 6.5×10^{9} vg/g or 6.5×10^{10} vg/g had minimal axonal degeneration and, sometimes, minimal gliosis limited to the dorsal funiculus (white matter). Axonal degeneration was characterized by a few scattered nerve fibers (≤10 per entire cross section) consisting of dilated axonal myelin sheaths that were either empty or containing cellular debris and/or phagocytic cells or spheroids (swollen eosinophilic axons). Gliosis was co-localized with axonal degeneration and was characterized by a few (≤10) more fusiform nuclei (i.e., microglial cells) near affected axons. The limited extent of the degeneration in this study supported an interpretation that these changes were non-adverse.

Minimally increased cellularity, related to enhanced numbers of satellite glial cells, in small clusters occurred in the DRG of a few PR006A-treated animals at the terminal sacrifice. This change was noted in 1 male administered 6.5×10^{9} vg/g (Group 2) and 1 male and 1 female administered 6.5×10^{10} vg/g (Group 3). This finding was characterized by rare (1 to 2 per section), loosely packed aggregates of oval- to spindle-shaped cells located among DRG neurons. The finding was consistent with a glial reaction to a prior neuronal injury and is a known consequence of gene therapy investigational products (i.e., AAV vectors) designed for CNS targets.

An apparent increase in DRG autophagy was noted in investigational product-treated males, a common background finding in cynomolgus monkeys. This finding was considered not to

be PR006A-related because the number of animals at the terminal sacrifice was small and a few controls also had the finding; thus, ganglion autophagy was considered not PR006A-related.

At Days 7 and 30, minimal or slight mixed cell infiltrates and/or minimal hemorrhage in the brain (characterized by focal areas of meningeal perivascular accumulations of lymphocytes, macrophages, neutrophils, and erythrocytes) were observed in controls and PR006A-treated animals. In addition, at Day 183, PR006A-treated animals had increased injection site muscle degeneration or mononuclear infiltrates at injection sites. The findings were of minimal severity and the number of animals in the terminal sacrifice was small. Therefore, all of these findings were considered procedure related (intracisternal injection) and not related to PR006A.

Overall, there have been no adverse PR006A treatment-related findings observed in the mouse or NHP studies.

1.2 Study Rationale

1.2.1 Overview

The purpose of this Phase 1/2 open-label study is to assess the safety, tolerability, immunogenicity, and effects of PR006A on PGRN levels in plasma and/or CSF, as well as its immunogenicity and effects on biomarkers and efficacy parameters, in order to inform further clinical investigation and/or allow marketing registration of PR006A in a patient population suffering from FTD-GRN.

1.2.2 Rationale for Patient Population

This study will enroll FTD-GRN patients at the symptomatic stage of the disease, based on results of a CDR plus NACC FTLD SB score of at least 1 and less than or equal to 15. Pathogenic *GRN* mutation carrier status will be confirmed during Screening; patients with all null mutations including nonsense, frameshift, splice site mutations, and complete or partial (exonic) *GRN* gene deletions will be considered for enrollment. Carriers of *GRN* missense mutations may be included provided a specific mutation has previously been demonstrated to be pathogenic.

Individuals harboring pathogenic *GRN* mutations have PGRN haploinsufficiency with an approximately 50% decrease of normal PGRN levels and are known to be at 90% risk to develop FTD by 75 years of age. Hence, the PR006A mechanism of action is relevant to patients with pathogenic *GRN* mutations. The Sponsor's clinical hypothesis is that treatment with PR006A will increase brain PGRN levels, modify the disease processes, and slow or halt clinical progression.

1.2.3 Rationale for Staggering of Patient Enrollment

Based on available nonclinical data, no specific safety signals are anticipated in humans, other than potential risk for liver enzyme elevations related to AAV9-capsid-specific T-cell responses observed in previous human studies with AAV9 vectors (Mendell et al 2017). In order to monitor for any unexpected safety events in humans, including but not limited to potential transitory liver enzyme elevations, in each cohort, enrollment will be staggered by at least 8 weeks between the first 2 patients and then at least 8 weeks between the second patient and the remaining 3 patients of the cohort to permit review of safety, tolerability, immunogenicity, and, to the extent available, efficacy by the iDMC, who will ultimately provide a recommendation on further dosing. This 8-week monitoring period was established to capture the reported time-frame for liver enzyme elevations caused by T-cell responses, which were observed at approximately 3 weeks post-dosing (Mendell et al 2017). It also ensures the iDMC will have appropriate biomarker and safety data from the Month 2 visit to make their recommendation. Since presumably, CSF inflammatory changes occur between 1 and 2.5 months post-dosing, at least 8 weeks after the second staggered patient is dosed in Cohort 1, the iDMC will review all available safety, tolerability, immunogenicity, and, to the extent feasible, efficacy results from the first 2 patients in this cohort (dosed in a staggered manner). At the recommendation of the iDMC, the Sponsor will decide whether to dose the remaining 3 patients in Cohort 1 (low dose) without staggering and in parallel begin staggered enrollment in the mid-dose cohort (Cohort 2).

Escalation to the mid-dose (7.0×10^{13} vg) [redacted] level cohorts will be based on iDMC recommendation, assessments of safety and biomarker data from the clinical PR006A low- and mid-dose cohorts, as well as technical feasibility (e.g., the ability to obtain sufficient concentrations of PR006A within the limitations of injectate volume).

Once enrollment of at least 2 patients in the low-dose cohort is complete and at least 8 weeks have elapsed after the second patient is dosed, the iDMC will review cumulative tabulated

data from the low-dose cohort and recommend whether or not to initiate dosing in the mid-dose cohort. The mid-[redacted] dose cohorts will utilize the same approach (staggered patients followed by unstaggered patients) as the low-dose cohort. The same iDMC data review process will be followed once at least 2 patients have enrolled in the mid-dose cohort and at least 8 weeks have elapsed after the second patient is dosed, [redacted]

The Sponsor will perform 3 formal interim analyses: 1) after all patients complete 12 months of treatment in Cohort 1; 2) after all patients complete 12 months of treatment in Cohort 2; [redacted]. Additionally, the Sponsor will perform a review of biomarker data at regular intervals. Based on the review of data through Month 2 in at least 2 patients and in consultation with the iDMC, a decision will be made to continue or suspend enrollment in any enrolling cohorts and to start enrollment in the subsequent cohort (i.e., dose escalate). A decision to dose escalate will be based on data from at least 2 patients from the concurrent cohort. However, all cohorts will enroll a minimum of 3 patients, unless enrollment is terminated due to safety concerns or based on biomarker review this dose is considered to be non-efficacious. In all other circumstances, the full cohort of 5 patients will be enrolled. Additional interim analyses may be performed during the follow-up period (post-Year 1) based on Sponsor's discretion.

1.2.4 Rationale for Route of Administration

PR006A will be delivered via a single suboccipital injection into the cisterna magna. This delivery method was chosen because in NHP studies it has been shown to result in broad biodistribution and cellular transduction throughout the brain (Hinderer et al 2014; Sorrentino et al 2016; Hinderer et al 2018; Hordeaux et al 2018; Ohno et al 2018). Furthermore, CNS transduction is less likely to be affected by the presence of existing or elicited AAV9 neutralizing antibodies in the periphery compared to an intravenous route of administration (Samaranch et al 2012; Gray et al 2013; Hinderer et al 2014; Meyer et al 2015; Hinderer et al 2018). The procedure will be performed by an interventional radiologist or neurosurgeon who has experience with the procedure and using imaging guidance (with computed tomography [CT] fluoroscopy or 3-dimensional intraoperative imaging) while the patient is under general anesthesia or deep sedation.

1.2.5 Rationale for Dosing

The dose rationale is based on published human clinical, biomarker, and genetic data on *GRN* mutations and their causative effects in FTD; nonclinical efficacy, biodistribution, and safety results obtained in a mouse model of FTD-GRN; and biodistribution and safety results obtained in NHP studies.

Patients with FTD-GRN carry a germline mutation in 1 of the 2 chromosomal alleles of the *GRN* gene, resulting in a lifelong reduction of CSF and blood PGRN levels by approximately 50%. The goal of the PR006A therapy is to restore CNS PGRN levels as measured in the CSF. Our hypothesis is that restoring CNS PGRN to a normal level, or higher, will translate into a slowing or halting of clinical progression of the disease and potentially improvement of ongoing symptoms.

The starting dose of 3.5×10^{13} vg translates to a dose of 2.7×10^{10} vg/g brain weight, assuming an adult brain mass of 1.3 kg (Hakim and Mathieson 1979). In the *Grn* KO mouse studies, 2.7×10^{10} vg/g brain weight was the lowest dose tested that resulted in efficacy across the multiple key endpoints. In the multidose study, PR006A treatment resulted in a dose-dependent brain transduction of the *GRN* transgene and dose-dependent expression of progranulin mRNA and PGRN.

Equally important, the dose of 2.7×10^{10} vg/g brain weight improved the expression of several neuroinflammatory markers including the mRNA-level specific markers *Tnfa* and *Cd68* and significantly reduced the accumulation of lipofuscin in the brain, which is a pathologically relevant endpoint. Similar effects were also observed at the higher dose level.

Therefore, based on the results of *Grn* KO mouse efficacy study, a PR006A dose of 2.7×10^{10} vg/g brain would be predicted to increase brain PGRN levels to approximately a normal level and ameliorate essential pathological features seen in FTD-GRN, such as neuroinflammation and lipofuscin deposition, in FTD-GRN patients.

Regarding safety, no adverse findings were observed in the 6-month GLP study performed in NHPs administered PR006A at the dose levels of 6.5×10^{9} vg/g brain and 6.5×10^{10} vg/g brain. Furthermore, no PR006A-related safety findings were observed in the mouse studies up to the highest tested dose level of 2.7×10^{11} vg/g brain. The GLP NHP study establishes a

safety margin of 2.4x to the starting clinical dose; the *Grn* KO mouse studies establishes a safety margin of 10x to the starting clinical dose.

The GLP NHP study also served to evaluate biodistribution and transgene expression following ICM administration. ICM administration of PR006A resulted in widespread and broad transduction of the human *GRN* transgene in the brain and peripheral organs and in a dose-dependent increase in gene expression. *GRN* mRNA levels were elevated in all brain areas and peripheral tissues with positive transduction. Progranulin levels were measured in the CSF with an experimental method detecting both NHP and human PGRN. Treatment with the lower dose of PR006A (6.5×10^9 vg/g) and higher dose (6.5×10^{10} vg/g) resulted in a 34% and 119% increase, respectively, in CSF PGRN compared to levels measured in control NHPs. The NHP biodistribution and *GRN* expression results provide further support for the efficacy of PR006A treatment and for the starting clinical dose, which is between the 2 dose levels tested in this NHP study.

The interspecies dose comparisons above are based on brain weight (vector genomes of PR006A per gram brain weight). To further assure translation of mouse and NHP results to dosing in patients and taking into account both the size of the brain and the route of administration, biodistribution observed in the 2 species was compared. In the mouse model, ICV administration of the mid dose of 2.7×10^{10} vg/g brain of PR006A resulted in brain vector genome exposure levels of $3.2 \times 10^3 \pm 0.9 \times 10^3$ vg/µg DNA (mean ± standard error of the mean) at Day 90. In NHPs, ICM administration of 6.5×10^9 vg/g and 6.5×10^{10} vg/g of PR006A resulted in brain vector genome exposure of, respectively, $7.5 \times 10^2 \pm 3.9 \times 10^2$ vg/µg and $2.9 \times 10^4 \pm 1.3 \times 10^4$ vg/µg DNA (pooled data of all brain areas; mean ± standard error of the mean) in the Day 183 cohort. These results support that interspecies dose comparisons based on vector genomes per gram brain weight are appropriate for selection of the human starting dose.

Sequential escalation to the mid-dose (7.0×10^{13} vg) [redacted] level cohorts will occur in conjunction with iDMC recommendation, who will review pertinent safety, biomarker, and clinical data. These dose levels are a 2x [redacted] increase over the starting dose level.

During the conduct of the PRV-FTD101 clinical study, the Sponsor will also perform review of biomarker data at regular intervals. Based on this review and in consultation with iDMC, a

decision to suspend further enrollment in one cohort and start enrollment in the next higher dose cohort may be taken. A minimum of 3 patients in each cohort must be dosed prior to effectuating dose escalation.

In summary, the clinical starting dose of 2.7×10^{10} vg/g brain is predicted to be well tolerated, increase PGRN levels in the CNS, result in improvements of FTD-GRN pathology, and therefore potentially be clinically beneficial in FTD-GRN patients.

Refer to the investigator's brochure (IB) for a detailed description of the PR006A nonclinical data.

Rationale for immunosuppressant regimen

Intracisternal administration has been chosen to maximize the distribution of PR006A throughout the CNS and minimize systemic exposure to the viral capsid of AAV9 and the T-cell response, but inevitably there will be some escape of the virus out of the CSF and into the blood. In clinical studies, gene therapy candidates using AAV9 have been generally well-tolerated. Transient elevations in alanine aminotransferase (ALT) and/or aspartate aminotransferase (AST) levels, which respond to corticosteroid treatment, have been observed to occur approximately 3 weeks after treatment (Mendell et al 2017). Additionally, the second immunosuppressive agent, sirolimus, has been shown to suppress AAV-related and transgene protein expression-related immune responses in NHPs (Mingozzi et al 2007; Ramsingh et al 2018), is reported to be beneficial in animal models of FTD (Wang et al 2012), and has a manageable safety profile which is considered steroid-sparing (Pidala et al 2020).

Therefore, in order to mitigate ALT and/or AST elevations, inflammatory changes in the CSF or other suspected immune system reactions, 1 loading dose of 6 mg oral sirolimus will be administered at Day -1, as well as a single dose of 1 g intravenous (IV) methylprednisone at Day 0. A concomitant maintenance dose of 2 mg/day sirolimus will be administered orally for 90 days starting at Day 0, which will then be tapered over the ensuing 15 to 30 days. The maintenance dose is administered to achieve trough levels of 4 ng/mL (range 2 to 8 ng/mL). One day after treatment with PR006A, patients will start prednisone 30 mg orally for 14 days followed by a 7-day taper.

At the Investigator's discretion, higher doses, a longer taper, or re-initiation of immunosuppressive treatment may be used (e.g., in cases of elevated AST or ALT, inflammatory changes in the CSF, or other suspected immune system reactions).

1.3 Risk/Benefit

This is a first-in-human study with PR006A, a first-in-class AAV9 vector-based gene therapy that transduces a functional copy of the human *GRN* gene. The population PR006A is intended for has FTD caused by the *GRN* mutation. FTD is a devastating neurological disorder in which there are no approved therapies. Available clinical and nonclinical data strongly support the role of PGRN reduction in pathogenic *GRN* mutation carriers as a causal factor in developing FTD, and that restoring PGRN levels will slow, halt, or possibly reverse the disease process. Such changes in disease pathology may translate into clinical benefit. However, there is no previous clinical data to indicate that increasing PGRN levels in the CNS will be beneficial, and it is not known whether increasing PGRN levels at the symptomatic stage of the disease will help prevent or slow downstream neurodegeneration.

In nonclinical efficacy and toxicology models, no PR006A-related adverse events (AEs) have been observed at the proposed starting dose. While minimal axonal degeneration and gliosis limited to the dorsal funiculus as well as minimally increased cellularity in small clusters occurred in the DRG of few PR006A-treated NHPs, these findings were non-inflammatory, limited in their extent, and without clinical correlate, and therefore were deemed non-adverse (PRV-2018-028). It is not predicted that the non-adverse findings in the NHPs will clinically translate to human patients. Notwithstanding, the protocol will exclude individuals with clinical evidence of peripheral symmetric sensory polyneuropathy and incidence of sensory symptoms will be monitored during the study using the Treatment-Induced Neuropathy Assessment Scale (TNAS).

PR006A immunogenicity in humans is currently unknown and will be studied as part of planned clinical studies. Overall, products (investigational and marketed) using the AAV9 vector have been safe and generally well tolerated in over 500 patients. Out of an estimated 21 clinical trials investigating AAV9-based gene therapies, 7 use an IT route of administration with approximately 60 patients having received an investigational therapy. In addition, all study participants will receive prophylactic corticosteroids and sirolimus to mitigate the risk of an immune response and will undergo longitudinal measurements for humoral and immune cellular responses. In the unlikely event of more pronounced immune

reactions in spite of prophylactic corticosteroid treatment, a consultation with clinical immunologist should be considered. Immune response to the transgene protein (PGRN) is not expected in heterozygous haploinsufficient patients, as they naturally express PGRN at approximately 50% of normal levels.

Patients will receive a prophylactic steroid-sparing immunosuppressive regimen consisting of corticosteroids and sirolimus, intended to alleviate AAV9-related T-cell driven immune responses. Similar steroid-sparing approaches have demonstrated safety benefit and have been implemented in other gene therapy programs (e.g., Bharucha-Goebel et al 2020; Nevoret et al 2020). Moreover, sirolimus has been shown to suppress AAV-related immune responses and increase transgene protein expression in NHPs (Mingozzi et al 2007; Ramsingh et al 2018), is reported to be beneficial in animal models of FTD (Wang et al 2012), and has a manageable safety profile allowing the reduction or elimination of steroid usage (Pidala et al 2020).

Despite the beneficial effects of corticosteroids and sirolimus in the prevention and management of potential capsid and transgene immune reactions, both immunosuppressive agents are associated with a variety of adverse events (AEs). Systemic steroids at high doses can produce a variety of AEs ranging from mild irritability to life-threatening or even fatal biochemical effects including hyperglycemia, hypertension, and psychiatric disorders A number of precautions should be considered with sirolimus usage, such as but not limited to increased risk of infections, malignancies/ lymphomas, angioedema, impaired wound healing, interstitial lung disease, hyperlipidemia, decline in renal function, proteinuria, embryo-fetal toxicity and male infertility. The use of live vaccines should be avoided during treatment with sirolimus. Sirolimus should not be used with strong CYP 3A4/P-gp inducers or inhibitors and requires monitoring of trough levels in the blood. If steroid or sirolimus side effects emerge, these drugs will be stopped or tapered as medically indicated.

Progranulin is a secreted glycoprotein associated with the regulation of lysosome function and inflammation (Chitramuthu et al 2017). Progranulin is broadly expressed in the CNS and periphery in a variety of cell types (Chitramuthu et al 2017). Progranulin expression has been reported to be upregulated in certain tumors and tumor cell lines (Serrero 2003; Chitramuthu et al 2017). Furthermore, overexpression of PGRN in vitro, in tumor cell lines or primary tumor cells, has been reported to increase certain tumorigenic properties in some contexts (He et al 2002; Chitramuthu et al 2017) but not in other contexts

(Matsumura et al 2006). In contrast to these studies, overexpression of PGRN in vivo has been reported by several groups using AAV or lentiviral transduction and transgenic mouse approaches, without findings of tumor formation (Tao et al 2012; Altmann et al 2016; Arrant et al 2018). Furthermore, direct intravenous administration of PGRN in vivo has not been reported to be associated with tumorigenesis (Tang et al 2011). Consistent with these findings, the Sponsor's in vivo studies using PR006A to overexpress PGRN in mouse models or normal NHPs have not revealed tumorigenic potential.

Based on the above analysis, an increased risk of malignancy is considered by the Sponsor to be unlikely in the context of PR006A administration. Patients with a history of cancer within 5 years of Screening or current presence of pre-cancerous lesions will be excluded from study participation, with the exception of fully excised prostate carcinoma in situ and fully excised nonmelanoma skin cancers that have been stable for at least 6 months. Patients will also be required to have up to date age- and gender-appropriate cancer screenings, completed as per the Investigator's judgment and local standard of care, prior to inclusion/exclusion Screening. Additionally, the occurrence of malignancy at any point after gene transfer that is judged as at least probably related to PR006A per the Sponsor's final causality assessment is one of the study stopping rules (Section 4.2.4).

Additionally, several measures will be taken to ensure the safety of patients who participate in this study, and the collected data will also be reviewed by an iDMC (Section 11.1.1), which will provide recommendations regarding dosing additional individuals. Vital signs, blood chemistry, coagulation, hematology, and urinalysis will be monitored. In addition, specific measures will be implemented to address safety considerations for PR006A and mitigate for potential corticosteroids and sirolimus side effects.

In order to minimize risk, all sites participating in this study will be trained on investigational product administration via intracisternal injection using modern imaging techniques to maximize patient safety and comfort. This study will be conducted in compliance with the protocol, Good Clinical Practice (GCP), and applicable regulatory requirements.

2 Study Objectives and Endpoints

Objectives	Endpoints
**Primary**	
• Evaluate the safety, tolerability, and immunogenicity of [redacted] dose levels of PR006A administered via suboccipital injection into the cisterna magna	Safety endpoints measured up to 5 years:<br>• Incidence and severity of TEAEs and serious AEs (SAEs), including clinically significant changes in vital signs, clinical laboratory assessments, Columbia Suicide Severity Rating Scale (C-SSRS), and TNAS; treatment-emergent transaminitis; treatment-emergent diabetes and prediabetes; treatment-emergent peptic ulcer disease and/or erosive gastritis; treatment-emergent osteoporosis; treatment-emergent increase in body weight ≥5% from baseline, treatment-emergent body mass index (BMI) >25 or >30, treatment-emergent increase in waist circumference ≥15% from baseline; incidence of Grade ≥3 infections; incidence/aggravation of psychosis; treatment-emergent hypercholesterolemia or hyperlipidemia; treatment-emergent opportunistic infections (including progressive multifocal leukoencephalopathy (PML) and BK virus-associated nephropathy); sirolimus trough levels; 12-lead ECGs; physical examinations; and neurological examinations<br>• Sum of adverse reactions (ARs) and suspected ARs<br>• Sum of serious ARs and serious suspected ARs<br>• Incidence of procedure or treatment-emergent safety findings as per brain MRI<br>• Change from baseline in immunogenicity of AAV9 and PGRN in blood at Day 14 and Months 1, 2, 3, 6, 9, and 12, and in CSF at Months 2 and 12

• Quantify PGRN levels in blood and CSF	• Change from baseline in PGRN levels in blood at Months 1, 2, 3, 6, 9, and 12<br>• Change from baseline in PGRN levels in CSF at Months 2 and 12
**Secondary**	
To evaluate the effect of PR006A on:<br>• CDR plus NACC FTLD	• Change from baseline in CDR plus NACC FTLD at Months 6 and 12
• NfL levels in blood and CSF	• Change from baseline in NfL levels in blood at Months 2, 6, 9, and 12<br>• Change from baseline in NfL levels in CSF at Months 2 and 12
[redacted]	

[redacted]

[redacted]	[redacted]
[redacted]	[redacted]

3 Investigational Plan

3.1 Study Design

Study PRV-FTD101 is a Phase 1/2, multi-center, open-label, ascending dose, first-in-human study that will evaluate the safety and effect on PGRN levels of intracisternal PR006A administration in patients with FTD-GRN. [redacted] escalating-dose cohorts are planned (3.5×10^{13} vg, 7.0×10^{13} vg, [redacted] of PR006A).

This is a 5-year study. During the first year, patients will be evaluated for the effect of PR006A on safety, tolerability, immunogenicity, biomarkers, and efficacy. Patients will follow up for an additional 4 years to continue to monitor safety, selected biomarkers, and efficacy parameters.

To minimize patient burden due to travel for evaluations, and in certain circumstances where the study site is unable to perform the PR006A administration procedure, patients will be permitted to be dosed at one study site and complete follow-up visits after dosing at a separate study site close to their home, with Sponsor's approval. The study site where follow-up evaluations will be performed must be an active site in the PRV-FTD101 study.

3.1.1 Year 1

Fifteen patients will be administered a one-time dose of PR006A, suboccipitally injected into the cisterna magna by an interventional radiologist or neurosurgeon.

In each cohort, enrollment will be staggered by at least 8 weeks between the first 2 patients and then an additional 8 weeks between the second patient and the remaining 3 patients of the cohort to permit review of safety, tolerability, immunogenicity, and, to the extent available, efficacy by the iDMC, who will ultimately provide a recommendation on further dosing. At least 8 weeks after the second staggered patient is dosed in Cohort 1, the iDMC will review all available safety, tolerability, immunogenicity, and, to the extent feasible, efficacy results from the first 2 patients in this cohort (dosed in a staggered manner). At the recommendation of the iDMC, the Sponsor will decide whether to dose the remaining 3 patients in Cohort 1 (low dose) without staggering and in parallel begin staggered enrollment in the mid-dose cohort (Cohort 2).

Cohort 2 will enroll 5 patients to receive the mid-dose (7.0×10^{13} vg) of PR006A, and [redacted]. The design for Cohorts 2 [redacted] enrollment is the same as for Cohort 1.

If a decision is made to not open or to limit enrollment into a cohort (i.e., Cohort 2 and/or [redacted] due to safety or tolerability reasons, the unenrolled subjects may be re-allocated to a lower dose level cohort.

One loading dose of 6 mg oral sirolimus will be administered at Day -1, as well as a single dose of 1 g IV methylprednisone at Day 0. A concomitant maintenance dose of 2 mg/day sirolimus will be administered orally for 90 days starting at Day 0 which will then be tapered over the ensuing 15 to 30 days. The maintenance dose is administered to achieve a trough sirolimus concentration of 4 ng/mL (range 2 to 8 ng/mL). One day after treatment with PR006A, patients will start prednisone 30 mg orally for 14 days, followed by a 7-day taper. At the Investigator's discretion, adjustments of dose and duration are permitted.

During the dose-escalation period of the study, the Sponsor will perform a review of biomarker data at regular intervals. Based on the review(s) and in consultation with the iDMC, a decision may be taken to suspend enrollment in the low-dose cohort and start enrollment in mid-dose cohort or suspend enrollment in the mid-dose cohort [redacted], depending on the stage of dose escalation. A decision to dose escalate will be based on data from at least 2 patients from the concurrent cohort. However, all cohorts will enroll a minimum of 3 patients, unless enrollment is terminated due to safety concerns or based on biomarker review this dose is considered to be non-efficacious. In all other circumstances, the full cohort of 5 patients will be enrolled.

An iDMC consisting of clinicians and a biostatistician otherwise unaffiliated with the conduct of the study will conduct a review of the safety, tolerability, immunogenicity, and available efficacy data in accordance with the charter. The iDMC will assess safety variables as listed for the primary safety endpoints outlined in Section 2.

The Sponsor will perform 3 formal interim analyses: 1) after all patients complete 12 months of treatment in Cohort 1; 2) after all patients complete 12 months of treatment in Cohort 2; [redacted]. Additionally, the Sponsor will perform a review of biomarker data at regular intervals. Based on the review of data through Month 2 in at least 2 patients and in consultation with the iDMC, a decision will

be made to continue or suspend enrollment in any enrolling cohorts and to start enrollment in the subsequent cohort (i.e., dose escalate). A decision to dose escalate will be based on data from at least 2 patients from the concurrent cohort. However, all cohorts will enroll a minimum of 3 patients, unless enrollment is terminated due to safety concerns or based on biomarker review this dose is considered to be non-efficacious. In all other circumstances, the full cohort of 5 patients will be enrolled.

3.1.2 Post-Year 1

Patients will have bi-annual follow-up site visits to assess the effect of long-term exposure to PR006A on safety, selected biomarker, and efficacy outcomes.

4 Patient Selection and Withdrawal Criteria

4.1 Selection of Study Population

Patients will be allowed to be re-screened with prior documented Sponsor approval; the Investigator, in consultation with the Sponsor, will determine which Screening assessments are to be repeated.

Deviations from the inclusion and exclusion criteria are not allowed because they can potentially jeopardize the scientific integrity of the study, regulatory acceptability, or patient safety. Therefore, adherence to the criteria as specified in the protocol is essential. No waivers to eligibility criteria will be granted by the Sponsor.

4.1.1 Inclusion Criteria

Each patient must meet all of the following criteria to be enrolled in this study:

1. Men or women aged 30 to 80 years (inclusive), at the time of informed consent.
2. Body weight range of ≥40 kg (88 lbs) to ≤110 kg (242 lb) and a BMI of 18 to 34 kg/m^2.
3. Has symptomatic FTD as per investigator assessment (bvFTD, PPA-FTD, FTD with corticobasal syndrome, or a combination of syndromes are allowed for enrollment).
4. Score ≥1 and ≤15 on CDR plus NACC FTLD SB.
5. Stable use of background medications at least 8 weeks prior to investigational product dosing.
6. Carrier of a pathogenic* *GRN* mutation confirmed by the central laboratory.

   *All null mutations including nonsense, frameshift, splice site mutations, and complete or partial (exonic) gene deletions:
   - All previously published pathogenic mutations, with proven functional deleterious effect (selected missense mutations may be included provided they are known to be pathogenic)
   - All pathogenic mutations listed in Molgen FTD database (http://www.molgen.ua.ac.be)
   - All new mutations with low plasma PGRN level (<70 ng/mL) based on central laboratory measurement.
7. Negative screening test for *Mycobacterium tuberculosis* (MTB) or documented negative MTB test within 1 year prior to Screening.

8. Age- and gender-appropriate cancer screenings are up to date and completed as per the Investigator's judgment and local standard of care prior to Screening.
9. Patient and/or patient's legally authorized representative (LAR) (where applicable by local regulation) has the ability to understand the purpose and risks of the study and provide written informed consent and authorization to use protected health information in accordance with national and local privacy regulations. The patient or LAR may also provide consent for future biomedical research in accordance with their national regulations; however, the patient may still participate in the study without providing consent for future biomedical research.
10. Patient has a reliable study partner/informant (e.g., family member, friend) willing and able to participate in the study as a source of information on the patient's health status and cognitive and functional abilities (including providing input into the rating scales). The study partner should have regular contact with the patient (in person or via phone/video communication). The study partner must sign a separate partner informed consent form (ICF) indicating that she/he understands the study requirements and is willing to participate and attend study visits requiring study partner input.
11. Women of nonchildbearing potential must be either surgically sterile (hysterectomy, bilateral tubal ligation, salpingectomy, and/or bilateral oophorectomy at least 26 weeks before Screening) or post-menopausal, defined as spontaneous amenorrhea for at least 2 years, with follicle-stimulating hormone level in the post-menopausal range at Screening based on the central laboratory's range.
12. Men and women of childbearing potential (i.e., ovulating, premenopausal, and not surgically sterile) must use a highly effective method of contraception consistently and correctly for the duration of the study including the long-term follow-up. Highly effective methods of contraception are those that, alone or in combination, result in a failure rate of less than 1% per year when used consistently and correctly (i.e., perfect use) and include the following for participants of childbearing potential:
    a. Combined (estrogen and progestogen containing) oral, intravaginal, or transdermal hormonal contraception associated with inhibition of ovulation
    b. Oral, injectable, or implantable progestogen-only hormonal contraception associated with inhibition of ovulation
    c. Intrauterine device

d. Intrauterine hormone-releasing system

e. Bilateral tubal ligation or bilateral tubal occlusion (performed at least 3 months prior to Screening)

f. Vasectomized partner (performed at least 3 months prior to Screening)

g. Sexual abstinence (no sexual intercourse)

Acceptable forms of contraception for male participants include:

a. Sexual abstinence (no sexual intercourse)

b. History of vasectomy (performed at least 3 months prior to Screening)

c. Condom with spermicide used together with highly effective female contraceptive methods if the female partner(s) is of childbearing potential (see above for list of acceptable female contraceptive methods)

13. Men must agree to abstain from sperm donation for the duration of the study, including long-term follow-up.
14. Women must agree to abstain from egg donation for the duration of the study, including long-term follow-up.
15. Women of childbearing potential cannot be pregnant or lactating/breastfeeding and must have a negative result for the serum pregnancy test (β-human chorionic gonadotropin) at Screening.
16. Patient is generally ambulatory and not dependent on a walker or wheelchair.
17. Patient is living in the community (i.e., not in nursing home); some levels of assisted living may be permitted at the discretion of the Investigator.
18. Pneumococcal pneumonia and shingles vaccines are required within 10 years of screening (allowed to be performed during screening but must be given at least 4 weeks prior to initiation of the immunosuppressant regimen).

4.1.2 Exclusion Criteria

Patients meeting any of the following criteria will be excluded from the study:

1. Diagnosis of a significant CNS disease other than FTD that may be a cause for the patient’s FTD symptoms or may confound study objectives.

2. Brain MRI/magnetic resonance angiography (MRA) imaging indicating clinically significant abnormality, including evidence of prior hemorrhage, infarct >1 cm^3 or >3 lacunar infarcts, or a structural or vascular abnormality deemed a contraindication to intracisternal injection.

3. Hypersensitivity or contraindications to corticosteroid and/or sirolimus use (including but not limited to osteoporosis with vertebral fractures within 1 year prior to Screening, poorly controlled diabetes [see Exclusion Criterion 5c], uncontrolled hypertension [see Exclusion Criterion 5f]), uncontrolled hyperlipidemia or hypercholesterolemia as per Investigator assessment, uncontrolled interstitial lung disease, or uncontrolled renal insufficiency).

4. Clinical evidence of peripheral symmetric sensory polyneuropathy (stable sensory mononeuropathies and radiculopathies are not exclusionary).

5. Concomitant disease or condition within 6 months of Screening that could interfere with, or treatment of which might interfere with, the conduct of the study or that would, in the opinion of the Investigator, pose an unacceptable safety risk to the patient or interfere with the patient's ability to comply with study procedures; including, but not limited to, the following:

    a. Evidence of clinically significant liver disease

    b. Unstable autoimmune disease requiring chronic immunosuppression

    c. Poorly controlled/not adequately managed diabetes (Screening hemoglobin A1c [HbA1c] ≥7%)

    d. History of unstable angina, myocardial infarction, chronic heart failure (New York Heart Association Class III or IV), or clinically significant conduction abnormalities (e.g., unstable atrial fibrillation) within 1 year prior to Screening

    e. Clinically significant 12-lead ECG abnormalities at Screening, as determined by the Investigator

f. Uncontrolled hypertension defined as: average of 3 systolic blood pressure [SBP]/diastolic blood pressure [DBP] readings >165/100 mm Hg at Screening, or persistent SBP/DBP readings >180/100 mm Hg within 3 months prior to Screening that, in the opinion of the Investigator, are indicative of chronic uncontrolled hypertension

g. History of cancer within 5 years of Screening or current presence of pre-cancer lesions, with the exception of fully excised nonmelanoma skin cancers and fully excised prostate carcinoma in situ that have been stable for at least 6 months

h. History or current alcohol or drug abuse within 2 years of Screening

i. Any current psychiatric diagnosis according to the Diagnostic and Statistical Manual of Mental Disorders Fifth Edition, International Statistical Classification of Diseases and Related Health Problems Tenth Revision, or equivalent, that may interfere with the patient's ability to perform study procedures and all assessments (e.g., psychosis, major depression, bipolar disorder, mental retardation, and schizophrenia). NOTE: Psychiatric manifestations of FTD are not exclusionary

j. At imminent risk of self-harm, based on clinical interview and responses on the C-SSRS. Patient must be excluded if they report ideation with intent, with or without a plan or method (i.e., positive response to item 4 or 5 on the C-SSRS) in the past 2 months or suicidal behavior in the past 6 months

k. Any medical disorders that, in the opinion of the Investigator, could interfere with study-related procedures (including safe performance of lumbar puncture (LP) or intracisternal injection), such as prohibitive spinal diseases, bleeding diathesis, clinically significant coagulopathy, thrombocytopenia, or increased intracranial pressure

l. Documented stroke or transient ischemic attack within 1 year prior to Screening

m. History of seizure or unexplained blackouts, with the exception of seizure due to known, transient cause (e.g., medication, electrolyte disturbance), within 10 years prior to Screening

n. Currently active infection or severe infection (e.g., pneumonia, septicemia, CNS infections [e.g., meningitis, encephalitis]) within 12 weeks prior to Screening

o. History of severe allergic or anaphylactic reactions. History of hypersensitivity to any inactive ingredient of the investigational product (refer to the IB) or protocol-required immunosuppressant medications

p. Clinical evidence of vitamin B_{12} deficiency or vitamin B_{12} level less than the lower limit of normal if deemed clinically significant as per Investigator's assessment at Screening

q. History of neurosyphilis or history of syphilis infection without documentation of adequate treatment.

r. Subject is generally frail or has any medical condition, for which in the view of the investigator, participation in the study would not be in the best interest of the subject or is likely to prohibit further participation during the study period

6. Clinically significant abnormalities in laboratory test results at Screening as given below (laboratory testing may be repeated with medical monitor approval):

    a. Total bilirubin, alanine aminotransferase, or aspartate aminotransferase >1.5 × the upper limit of normal (ULN) (note: patients with confirmed Gilbert syndrome are allowed for enrollment with Sponsor's agreement)

    b. Serum creatinine >1.5 × ULN

    c. Hematocrit <35% for men and <32% for women

    d. Absolute neutrophil count <1500/µL

    e. Platelet count <100 000/µL

    f. International normalized ratio >1.4 or other coagulopathy.

    g. Activated partial thromboplastin time >50 seconds

    h. Thyrotropin (TSH) level outside the normal range and deemed clinically significant by the Investigator

i. Positive result for hepatitis B surface antigen, hepatitis C antibody, or human immunodeficiency virus (HIV) 1 or 2.

j. Any other abnormal Screening laboratory test result deemed clinically significant by the Investigator.

7. Participation within 3 months prior to Screening in another therapeutic investigational drug or device study with purported disease-modifying effects on FTD, unless it can be documented that the patient received placebo only.
8. Any type of prior gene or cell therapy.
9. Immunizations (live vaccines) in the 4 weeks prior to Screening. Note: Pneumococcal vaccine and shingles vaccine administration is allowed during the Screening Period (patients not previously vaccinated should receive pneumococcal and/or shingles vaccine administration at least 4 weeks prior to sirolimus loading dose).
10. Use of blood thinners (e.g., warfarin, heparin, and novel oral anticoagulants) in the 2 weeks prior to Screening or the anticipated need to initiate blood thinners during the study. Antiplatelet therapies (prophylactic aspirin, clopidogrel) are acceptable if the patient is medically able to temporarily stop from at least 7 days prior to and at least 48 hours after intracisternal injection and LP.
11. Contraindications or intolerance to imaging methods (MRI, CT) inducing claustrophobia and intolerance to contrast agents used for MRI or CT (including but not limited to gadolinium contrast agents and iohexol).
12. Contraindications to general anesthesia or deep sedation.
13. Positive urine test for drugs of abuse (including opiates, amphetamines, cocaine, barbiturates, and phencyclidine) without prescription at Screening and Day -1. Note: Use of medical marijuana is permitted provided that the patient is on a stable regimen. It is also permitted if the patient resides in a state in which the recreational use of marijuana is legalized, so long as the patient does not meet drug abuse criteria (as defined in the Diagnostic and Statistical Manual of Mental Disorders, Fifth Edition).

4.2 Withdrawal of Patients from Study Treatment and/or the Study

The duration of the study is defined for each patient as the date signed written informed consent is provided through the last follow-up visit.

4.2.1 Reasons for Withdrawal/Discontinuation

Patients may withdraw from the study at any time and for any reason without prejudice to their future medical care by the Investigator or at the study site that is provided outside of this study. An excessive rate of withdrawals can affect the integrity of the study, and unnecessary withdrawal of patients should be avoided. The Investigator also has the right to withdraw a patient from the study in case of events as provided below or for other reasons. The reasons for patients not completing the study will be recorded in the electronic case report form (eCRF). Prior to discontinuing any patients, the Investigator should discuss the reasons with the Sponsor or delegate.

A patient may be withdrawn from the study for any of the following reasons:

1. The patient withdraws consent or requests discontinuation from the study for any reason.
2. Occurrence of any medical condition or circumstance that exposes the patient to substantial risk and/or does not allow the patient to adhere to the requirements of the protocol.
3. Any SAE, clinically significant AE, severe laboratory abnormality, intercurrent illness, or other medical condition that indicates to the Investigator that continued participation is not in the best interest of the patient.
4. Patient failure to comply with protocol requirements or study-related procedures.
5. Termination of the study by the Sponsor or the regulatory authority.

Upon occurrence of a serious or intolerable AE, the Investigator will confer with the Sponsor. If a patient is discontinued because of an AE, the event will be followed, to the greatest extent possible, until it is resolved or stabilized.

4.2.2 Handling of Withdrawals

Patients are free to withdraw from the study at any time. Patient participation in the study may be stopped at any time at the discretion of the Investigator or at the request of the Sponsor.

Since this study includes the provision of gene therapy as a single administration, all patients who receive investigational product should be strongly encouraged to complete visits for a minimum of AEs assessments, even if these happen via phone visit.

If a patient who received investigational product withdraws prematurely from the study for any reason, study site personnel should make every effort to complete the full panel of assessments scheduled for the end of treatment visit. Patients who withdraw during the treatment period will complete the Month 12 (Year 1) assessments, while patients who withdraw during the follow-up period will complete the Month 60 assessments. If the termination visit is more than 3 months prior to the scheduled Month 12 assessment, or more than 6 months prior to the Month 60 assessment for subjects who discontinue participation after Month 12, in addition to performing termination visit assessments, the investigator and study site personnel should make every effort to complete the Month 12 or Month 60 assessments, respectively, at the originally scheduled time points. The reason for patient withdrawal must be documented in the eCRF.

If a patient undergoes the administration procedure but for whatever reason does not receive investigational product, the patient may be withdrawn from the study with safety follow-up for 30 days.

In the case of patients being lost to follow-up, at least 3 documented attempts to contact the patients must be made and documented in the patients' medical records.

4.2.3 Replacements

Withdrawn patients will not be replaced.

4.2.4 Study Stopping Rules

Each patient will receive only 1 dose of PR006A once during the course of the study; hence, stopping rules apply only during the Screening Period. Patients who have been dosed with PR006A will be followed to the end of the study as per protocol.

If any one of the following criteria are met, PR006A dosing will be paused and further enrollment across all study sites will be halted until the iDMC evaluates all available study data and makes a recommendation:

- More than 5 × ULN increase in ALT, AST, or both in any patient after PR006A administration that is not manageable by corticosteroid treatment implemented according to AASLD guidelines (Manns et al 2010).
- The occurrence of 2 or more of any 1 of the following treatment-emergent AEs (TEAEs)/SAEs (not 2 single occurrences of different AE/SAE):
    - Subarachnoid hemorrhage, cerebral macrohemorrhage
    - Grade 3 and/or 4 allergic reactions, hypersensitivity reactions, cytokine release reactions
    - Persistent neurological symptoms at least possibly attributable to the ICM investigational product administration procedure
    - CNS infections (e.g., meningitis, encephalitis)
    - Seizures, stroke, or acute paralysis
- More than 1 patient experiences any Grade 3 or higher AE that is judged as definitely, probably, or possibly attributed to PR006A.
- More than 1 Grade 3 or higher SAE that may or may not be potentially related to PR006A and that poses either an immediate risk to the patient's health or is likely to adversely affect the patient's long-term health.
- Death of a patient, after having received PR006A, that is judged as definitely, probably, or possibly attributed to PR006A. The study will be temporarily stopped in order to undergo review by the institutional review board (IRB)/independent ethics committee (IEC), iDMC, and other health authorities.
- The occurrence of a malignancy at any point after gene transfer that is judged per the Sponsor's final causality assessment as related to PR006A.

The study may be terminated if one or more of the following criteria are met:

1. The Sponsor decides to terminate the study based upon an assessment of safety.
2. The Sponsor decides to terminate the study for administrative reasons.

5 Study Treatments

5.1 Method of Assigning Patients to Treatment Groups

This is an open-label study. No randomization or blinding will be performed.

5.2 Treatments Administered

PR006A is an investigational AAV9 gene therapy drug product transducing wildtype *GRN* gene. Please refer to the IB and Pharmacy Manual for further details regarding the study treatment.

5.3 Identity of Investigational Product

PR006A is an investigational gene therapy that utilizes an AAV9 viral vector to deliver DNA encoding wildtype *GRN*, the gene encoding PGRN, to a patient's cells.

[redacted] escalating-dose cohorts are planned (3.5×10^{13} vg, 7.0×10^{13} vg, [redacted] of PR006A).

Refer to the IB for further details related to PR006A.

5.4 Management of Clinical Supplies

5.4.1 Investigational Product Packaging and Storage

PR006A concentrate for the low, mid, [redacted] doses will be supplied as a frozen solution for injection in single-use polypropylene vials or Type I, Class A glass serum vials and stored in individually labeled vials at -60°C or lower.

Details regarding packaging and storage are provided in the Pharmacy Manual and the IB.

5.4.2 Investigational Product Preparation and Dispensing

PR006A doses are prepared by dilution, if necessary, using the formulation buffer and will be mixed thoroughly prior to the administration, while maintaining sterile conditions. Dose preparation will be conducted by a trained pharmacist/medical technologist in accordance with the Pharmacy Manual.

5.5 Investigational Product Administration

5.5.1 Immunosuppressant Administration

5.5.1.1 Corticosteroid Administration

Subjects will receive a loading dose of methylprednisolone 1 g IV pulse on Day 0. Prednisone at a dose of 30 mg/day will be given orally as concomitant medication from Day 1 for 14 days, which will be then tapered over the ensuing 7 days. At the Investigator's discretion, higher doses or a longer taper of corticosteroids may be used.

Mitigation of risks associated with use of corticosteroids, including monitoring for AEs that may be related to corticosteroid use are described in Section 6.2.2.2.

5.5.1.2 Sirolimus Administration

Patients will receive a sirolimus oral loading dose of 6 mg at Day -1. Subsequent sirolimus maintenance dose of 2 mg will be adjusted to maintain serum trough levels of 4 ng/mL (range 2 to 8 ng/mL) until the Month 3. Sirolimus will be subsequently tapered during the subsequent 15 to 30 days. Trough levels will be collected prior to administration of the sirolimus dose for each visit as presented in Schedule of Events (main study period). All sirolimus doses will be captured in the eCRF.

All patients and caregivers should be provided with a current version of the local manufacturer's medication guide, including instructions for use.

5.5.1.3 Immunosuppression Monitoring Criteria

In addition to monitoring sirolimus trough levels, the Investigator should evaluate patient's clinical status, lab findings and potential AEs.

Consideration should also be given to the need to increase doses of the immunosuppressant agent, prolong the tapering regimen, add a third agent or re-initiate treatment based on clinical signs or symptoms consistent with an immune response, including:

- Asymptomatic pleocytosis with WBC > 30 mm^3 and/or high CSF protein (> 70 mg/dL)

- CSF pleocytosis and/or increased protein accompanied by clinical symptoms (including decompensation of underlying FTD symptoms)
- Emergence of sensory symptoms based on neurological examination and/or TNAS.
- ALT and/or AST elevation >5 × ULN in conjunction with hepatitis symptoms (e.g., jaundice, fatigue)
- ALT and/or AST elevation >10 × ULN irrespective of the presence or absence of clinical symptomatology

The Investigator should consider implementing a longer prednisone taper over an additional 4 weeks in patients presenting with ALT and/or AST >3 × ULN at the end of the initial 14-day taper. In case of AST/ALT elevations refractory to prednisone treatment, the Investigator should seek expert advice from a hepatologist.

5.5.2 Pre-Cisternal Puncture Procedures

Patients will undergo standard of care medical evaluations in preparation for cisternal puncture, including anesthesiologist consultation. The neurosurgeon/interventional radiologist and anesthesiologist will review Screening clinical laboratory analyses (including documented negative pregnancy test), brain MRI and MRA, and local ECG results. Medical history and currently prescribed and over-the-counter medications will be reviewed with regards to any recent changes. At the anesthesiologist's discretion, additional clinical assessments may be performed (specific to concomitant medical conditions).

5.5.3 Intracisternal Injection

On Day 0, PR006A will be administered as a single dose via suboccipital injection into the cisterna magna by an interventional radiologist or neurosurgeon. Prior to injection, a volume of intracisternal fluid equivalent to the PR006A dosing volume will be removed. The procedure will be performed under general anesthesia or deep sedation and using imaging guidance in accordance with the Procedure Manual. Patients will remain under observation for 24 hours (overnight inpatient stay) after PR006A administration.

5.5.4 Investigational Product Accountability

The Investigator or designee is responsible for:

1. Logging receipt of each shipment of investigational product,
2. Confirming the actual shipment contents,
3. Verifying receipt of the investigational product by signing the appropriate documentation provided by the Sponsor or its designee, and
4. Indicating the status of each vial.

Accountability will be recorded on the appropriate eCRF. Where permitted, all investigational product will be kept on site until permission is granted by the Sponsor to destroy or return the investigational product. In the event hospital pharmacy requirements will not allow used investigational product vials to be retained for accountability, the site must maintain proper documentation to support accountability for all used investigational product vials no longer present at the site.

The Investigator will maintain accurate records of receipt of all investigational product, including dates of receipt. In addition, accurate records will be kept regarding when and how much investigational product is administered for each patient in the study. Reasons for departure from the expected administration regimen must also be recorded. At the completion of the study, to satisfy regulatory requirements regarding drug accountability and requirements of the Sponsor, all investigational product will be reconciled and returned or destroyed in accordance with written instructions of the Sponsor or according to local hospital pharmacy requirements.

5.5.5 Other Supplies

Ancillary supplies required for dosing will be provided to the investigational sites. Supplies will include syringes, needles, a tubing set, flush buffer formulation, and Omnipaque 240.

5.5.6 Overdose Management

An overdose is any dose of study treatment given to a subject or taken by a subject that exceeds the dose described in the protocol. Overdoses with corresponding clinical signs, symptoms or clinical sequelae should be captured on relevant AE/SAE sections in the eCRF within 24 hours of the site becoming aware of the overdose. Overdoses without signs or

symptoms do not need to be recorded as AEs but should be reported on the relevant sections in the eCRF within 24 hours of the site becoming aware of the overdose.

5.5.7 Product Quality Complaint

A product complaint is any written, electronic, or verbal expression of dissatisfaction regarding the identity, quality, reliability, safety, purity, potency, effectiveness or performance (applicable for approved marketed products) of a drug product after it is released for distribution.

In the course of conduct of the study, study personnel may become aware of a product complaint associated with the use of the investigational product. Study site personnel shall notify Prevail within 24 hours by forwarding the product complaint information via the contact information listed in the Pharmacy Manual. Where possible, personnel should segregate and retain any product, materials, or packaging associated with the product complaint until further instruction is provided by Prevail.

5.6 Treatment Compliance

Treatment compliance will be ensured by an interventional radiologist or neurosurgeon who administers the dose. The success and/or completeness of investigational product administration will be documented by the proceduralist and recorded by the study site personnel in the eCRF.

5.7 Prior and Concomitant Therapy

5.7.1 Excluded Medications and/or Procedures

The following medications and procedures are not allowed during this study:

- Other gene therapy or cell therapy.
- Use of any other investigational drug, devices, stem cells, or experimental therapy.
- Immunization 4 weeks prior to and during Screening, e.g., influenza, tetanus.
  Note: Pneumococcal vaccine and shingles vaccine administration is allowed during the Screening Period for patients without documented vaccination coverage within 10 years prior. Patients not previously vaccinated should receive pneumococcal and/or shingles vaccine administration at least 4 weeks prior to sirolimus loading dose.

- Use of antiplatelet therapies and blood thinners at least 7 days prior to and at least 48 hours after intracisternal injection and LPs.
- Patients are encouraged to follow all routinely scheduled immunizations as recommended by their local center for disease control and prevention, including seasonal vaccinations. Consideration should be given to adjusting of the vaccination schedule to avoid vaccination during the period of immunosuppressant administration (Section 5.5.1). Additionally, the administration of live vaccines is prohibited from 4 weeks prior to sirolimus loading dose and until sirolimus is discontinued.
- The following medications and procedures are not allowed during the period of sirolimus administration:
    - Use of strong inhibitors of CYP 3A4 or P-glycoprotein, including but not limited to bromocriptine, cimetidine, cisapride, clotrimazole, danazol, diltiazem, fluconazole, protease inhibitors (e.g., HIV and hepatitis C that include drugs such as ritonavir, indinavir, boceprevir, and telaprevir), metoclopramide, nicardipine, troleandomycin, and verapamil.
    - Use of strong inducers of CYP 3A4 or P-glycoprotein, including but not limited to carbamazepine, phenobarbital, phenytoin, rifapentine, and St. John's Wort

5.7.2 Restricted Medications and/or Procedures

Prophylactic use of antiplatelet therapies and blood thinners should be temporarily stopped for at least 7 days prior to and at least 48 hours after intracisternal injection and LPs. Use of strong inducers and inhibitors of CYP3A4/P-gp, including grapefruit juice and over the counter medications (e.g., St. John's wort) is prohibited at least 7 days prior to and at least 48 hours after sirolimus administration.

It is the intent of this protocol to maintain baseline FTD medications unchanged from baseline through the 12-month visit. However, medically necessary changes are permitted, but the reason for dose change must be documented on the appropriate eCRF. Study-specific assessments (safety and efficacy scales) should be performed prior to initiation or change in dose of symptomatic FTD medications.

Any FTD therapy will be collected via eCRF.

5.7.3 Documentation of Prior and Concomitant Medication Use

Use of all concomitant medications will be recorded in the patient's eCRF. The minimum requirement is that drug name and the dates of administration are to be recorded. This will include all prescription drugs, herbal products, vitamins, minerals, and over-the-counter medications. Any changes in concomitant medications also will be recorded in the patient's eCRF.

Any concomitant medication deemed necessary for the welfare of the patient during the study may be given at the discretion of the Investigator. However, it is the responsibility of the Investigator to ensure that details regarding the medication are recorded in full in the eCRF.

6 Study Assessments and Procedures

Before performing any study procedures, all potential patients and their study partners will sign ICFs for overall study participation. Consent for the patient and the study partner are not required to be obtained on the same day; however, consent must be obtained from each patient and their study partner before entering the study.

Patients willing to provide blood and CSF samples for future biomedical research will be required to sign an optional additional section of the main ICF.

Patients and their study partners will have the opportunity to have any questions answered before signing the ICFs. The Investigator must address all questions raised by the patient and/or study partner. The Investigator or designee will also sign the ICFs.

The schedules of activities by visit for this study are presented in Table 13-1 (main study period) and Table 13-2 (follow-up period). The recommended (though not required) sequence of study assessments is provided in Section 13.2. Although the staging and sequence of study assessments is recommended, it will not be a protocol deviation if these assessments are performed in a different order.

Detailed instructions for the conduct of study assessments and procedures will be provided in the Investigator's study manuals.

6.1 Efficacy Assessments

Secondary and exploratory efficacy assessments will also include use of the tools described in the ensuing sections. These include scales evaluating language, behavior, executive functions, and memory.

All patients will undergo longitudinal vMRI assessments to monitor downstream effect of PGRN-associated neurodegeneration on cortical thickness, and white matter lesions will be quantified.

6.1.1 CDR Plus NACC FTLD

The CDR has been widely used in Alzheimer therapeutic studies. It serves as both a functional assessment instrument and a global rating. The CDR plus NACC FTLD was developed in 2008 as a supplement to the CDR (Knopman et al 2008). It was designed to

assess the clinical status of patients across the FTLD spectrum of disorders. To capture key FTLD patient characteristics, the CDR plus NACC FTLD adds 2 domains to the CDR: 1) the Behavior, Comportment, and Personality domain; and 2) the Language domain. To complete the CDR plus NACC FTLD, both the study partner and the patient are interviewed using the same CDR semi-structured interview. The following method is used for calculating the CDR plus NACC FTLD in this study: CDR plus NACC FTLD SB is reached by adding each of the 8 domain scores together to yield a single score that ranges from 0 (no impairment) to 24 (severe impairment) (Miyagawa et al 2019). A CDR plus NACC FTLD global score will also be calculated as per the established method (Miyagawa et al 2020).

6.1.2 Montreal Cognitive Assessment

The MoCA is a rapid screening instrument (similar to the Mini-Mental State Examination). It was developed to be more sensitive to patients presenting with mild cognitive dysfunction. The MoCA assesses 8 domains: attention and concentration, executive functions, short-term and working memory, language, visuospatial abilities, conceptual thinking, calculations, and orientation (Nasreddine et al 2005).

6.1.3 Benson Complex Figure Copy and Recall

Benson Complex Figure copy and recall is intended to assess a patient’s visuoconstructional and visual memory functions. It involves the patient copying and recalling the Benson figure:

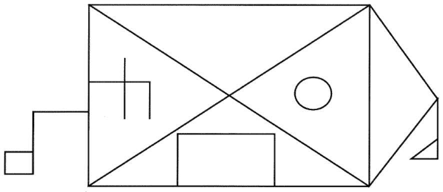

Accuracy and placement are each scored 0, 1, or 2 for the outer rectangle and for the peripheral elements, by the rater, usually a neuropsychologist. The scores are summed, and the lower the total score, the more impaired the patient is considered.

6.1.4 Clinical Global Impressions – Severity/Improvement

The CGI scale is a psychiatric rating instrument used to assess an Investigator's opinion of a patient's psychiatric functioning both before (CGI-S) and after (CGI-I) treatment with an investigational drug. Both the CGI-S and the CGI-I are single-item assessments (Busner and Targum 2007).

6.1.5 Trail Making Test, Part A and Part B

Both TMT-A and TMT-B consist of 25 circles distributed over a sheet of paper. In TMT-A, the circles are numbered 1 to 25, and the patient should draw lines to connect the numbers in ascending order. In TMT-B, the circles include both numbers (1 to 13) and letters (A to L); as in TMT-A, the patient draws lines to connect the circles in an ascending pattern, but with the added task of alternating between the numbers and letters (i.e., 1-A-2-B-3-C, etc.). The patient will be instructed to connect the circles as quickly as possible, without lifting the pen or pencil from the paper. The patient is timed as he or she connects the "trail." If the patient makes an error, this will be pointed out immediately and the patient allowed to correct it. Errors affect the patient's score only in that the correction of errors is included in the completion time for the task. The text should not be continued if the patient has not completed both parts after 5 minutes have elapsed. Results for both TMT-A and TMT-B are reported as the number of seconds required to complete the task; therefore, higher scores reveal greater impairment.

- For Trail A: Average is 29 seconds, deficient is >78 seconds, "rule of thumb" is most in 90 seconds.
- For Trail B: Average is 75 seconds, deficient is >273 seconds, "rule of thumb" is most in 3 minutes.

6.1.6 Digit Span Test (Forwards and Backwards)

The Digit Span Test (forwards and backwards) is a very short screening tool that evaluates the patient's cognitive status. It is administered by the Investigator or designee to quickly evaluate whether a patient's cognitive abilities are normal or impaired. Administering the test forwards assesses both attention and short-term memory; the backwards version of the test measures working memory.

The patient listens to the Investigator or designee saying a series of numbers and is asked to repeat them in the same order forwards and backwards. The first series is 3 numbers, such as "3, 9, 2." Each number is said in a monotone voice, 1 second apart. The patient should repeat those numbers forwards and backwards. The second series is 4 numbers, such as, "4, 7, 3, 1." Again, the patient should repeat those numbers forwards and backwards. The Investigator or designee should continue in the same manner by increasing the series of numbers to 5 and asking the patient to repeat the numbers forwards and backwards.

6.1.7 Category Fluency

The Category Fluency test involves the assessor asking the patient to name all the different examples that the patient can think of from that category in 1 minute. The categories are: animals and vegetables. The scores are summed; the higher the score, the better the category fluency.

6.1.8 Multilingual Naming Test

The MINT is a 32-item test designed to assess naming skills in speakers of multiple languages (Gollan et al 2012). The 32 items are selected from a set of 68 black and white line drawings presented in order of estimated increasing difficulty.

6.1.9 Magnetic Resonance Imaging Volumetric Measures

All patients will undergo MRI scans to evaluate safety and potential treatment effects. Imaging sequences will be performed as per the MRI Imaging Manual.

Global and regional brain volumes and regional cortical thickness will be derived from the vMRI sequence and will include cross-sectional absolute measurements and longitudinal volume change. Most of the regional cortical thicknesses will include anatomical areas that are most affected in FTD. Additionally, white matter lesions will be quantified in a longitudinal manner.

All MRI data collected (Screening and during treatment period) will be sent to the central imaging laboratory for quality control, image processing and analysis, and archiving. The MRI scan evaluation at Screening and post-dosing will be performed by the central imaging laboratory.

6.2 Safety Assessments

6.2.1 Adverse Events

6.2.1.1 Definitions of Adverse Events

An AE is defined as any unfavorable and unintended sign (e.g., an abnormal laboratory finding), symptom, or disease temporally associated with the use of a drug, without any judgment about causality or relationship to the drug. Patients will be instructed to contact the Investigator at any time after enrollment into the study (i.e., after signing the consent form) if any symptoms develop.

After signed written informed consent is provided but prior to ICM administration of investigational product, only AEs and SAEs related to a protocol-mandated intervention will be reported. All other AEs and SAEs during this time period will be recorded as medical history. After the ICM administration of investigational product, all AEs and SAEs will be reported.

A TEAE is defined as any event not present before exposure to investigational product or any event already present that worsens in either intensity or frequency after exposure to investigational product.

The Investigator is responsible for reporting all TEAEs (and predose AEs and SAEs related to a protocol-mandated intervention) that are observed or reported during the study, regardless of their relationship to investigational product or their clinical significance. Where known, a diagnosis should be recorded rather than individual signs, symptoms, or laboratory results.

Treatment-emergent AEs that may be associated with corticosteroid use should also be monitored (Section 6.2.2.2).

Adverse events may include:

- Any abnormal laboratory test results (hematology, blood chemistry, or urinalysis) or other safety assessments (e.g., ECG, radiological scans, vital sign measurements, C-SSRS), including those that are accompanied by clinical symptoms, considered clinically significant in the medical and scientific judgment of the Investigator (i.e., not related to progression of underlying disease), or require a medical intervention or change in concomitant medications.
- Exacerbation of a chronic or intermittent pre-existing condition including either an increase in frequency and/or intensity of the condition.
- New conditions detected or diagnosed after study intervention administration even though it may have been present before the start of the study.
- Signs, symptoms, or the clinical sequelae of a suspected overdose of a concomitant medication. Such overdoses should be reported regardless of sequelae.

Adverse events DO NOT Include:

- Any clinically significant abnormal laboratory findings or other abnormal safety assessments which are associated with the underlying disease, unless judged by the Investigator to be more severe than expected for the patient’s condition.
- The disease/disorder being studied or expected progression, signs, or symptoms of the disease/disorder being studied, unless judged by the Investigator to be more severe than expected for the patient’s condition.
- Medical or surgical procedure (e.g., endoscopy, appendectomy); the condition that leads to the procedure is the AE.
- Situations in which an untoward medical occurrence did not occur (social and/or convenience admission to a hospital).
- Anticipated day-to-day fluctuations of pre-existing disease(s) or condition(s) present or detected at the start of the study that do not worsen.
- Prescheduled or elective procedure or routinely scheduled treatment was scheduled (or on a waiting list to be scheduled) prior to obtaining the participant’s consent to participate in the study.

6.2.1.2 Serious Adverse Events

The ICH definition of an SAE is any untoward medical occurrence at any dose that:

- results in death
- is immediately life-threatening (Note: Life-threatening refers to an event that places the patient at immediate risk of death. This definition does not include a reaction that, had it occurred in a more severe form, might have caused death)
- requires inpatient hospitalization or prolongation of existing hospitalization. (Note: The following hospitalizations will not be considered SAEs: hospitalization for respite care, hospitalization for a pre-existing condition where either the hospitalization was planned prior to the study, or an elective surgery AND the patient did not experience an AE)
- results in persistent or significant disability/incapacity
- is a congenital anomaly/birth defect

Important medical events that may not result in death, be life-threatening, or require hospitalization may be considered SAEs when, based upon appropriate medical judgment, they may jeopardize the patient or may require medical or surgical intervention to prevent one of the outcomes listed in this definition. Examples of such medical events include allergic bronchospasm requiring intensive treatment in an emergency room or at home, blood dyscrasias or convulsions that do not result in inpatient hospitalization, or the development of drug dependency or drug abuse.

6.2.1.3 Eliciting and Documenting Adverse Events

Adverse events will be assessed from the time the patient signs the ICF until exit from the study. Only AEs and SAEs determined to be related to a protocol-mandated intervention will be reported from the time the patient signs the ICF until ICM administration of investigational product. After ICM administration of investigational product, all AEs and SAEs will be reported.

At every study visit, patients will be asked a standard nonleading question to elicit any medically related changes in their well being. They will also be asked if they have been

hospitalized, had any accidents, used any new medications, or changed concomitant medication regimens (prescription and over-the-counter medications).

In addition to patients' observations, AEs identified from any study data (e.g., laboratory values, physical and neurological examination findings, ECG and MRI changes) or identified from review of other documents (e.g., patient diaries) that are relevant to patient safety will be documented on the AE page in the eCRF.

6.2.1.4 Reporting Adverse Events

All AEs reported or observed during the study, as detailed in Section 6.2.1.3, will be recorded on the AE page in the eCRF. Information to be collected may include but is not limited to the following:

- drug treatment
- dose
- event term
- time of onset
- Investigator-specified assessment of severity and relationship to investigational product
- time of resolution of the event
- seriousness
- any required treatment or evaluations
- outcome

Adverse events resulting from concurrent illnesses, reactions to concurrent illnesses, reactions to concurrent medications, or progression of disease states must also be reported. All AEs will be followed to adequate resolution. The Medical Dictionary for Regulatory Activities (MedDRA) will be used to code all AEs.

Any medical condition that is present at the time that the patient is screened but does not deteriorate should not be reported as an AE. However, if it deteriorates at any time during the study, it should be recorded as an AE.

6.2.1.5 Suspected Adverse Reactions/Adverse Reactions

The following AEs will be considered as ARs:

- All AEs occurring within 1-month post-PR006A administration procedure
- All AEs assessed as at least possibly related to the injection procedure or to investigational product treatment by the Investigator or Sponsor
- All AEs for which the Investigator's causality assessment is missing or indeterminate

6.2.1.6 Reporting Serious Adverse Events

Any AE that meets SAE criteria (Section 6.2.1.2) from the time of signing the consent form until completion of the 4-year extended follow-up period (as detailed in Section 6.2.1.3) must be reported in the eCRF within 24 hours after the time that study site personnel first learn about the event. After the 5-year reporting window, any SAE that the Investigator considers related to investigational product must be reported to the contract research organization (CRO) or the Sponsor/designee.

Study site personnel should complete the AE eCRF using electronic data capture (EDC), designating the event as serious. If EDC is unavailable, study site personnel should complete an SAE form and report the SAE by calling the SAE Hotline or faxing the report using the information provided in the Contact List.

6.2.1.7 Expedited Reporting Requirements

All SAEs that occur during the SAE reporting period must be reported by entering the information into the study specific SAE report form and submitting to the CRO within 24 hours of the site becoming aware of the event. Investigators should not wait to collect information that fully documents the event before notifying Prevail of an SAE. Prevail may be required to report certain SAEs to regulatory authorities within 7 calendar days of being notified about the event; therefore, it is important that Investigators submit any information requested by Prevail as soon as it becomes available. Additional follow-up information, if required or available, should all be submitted to Prevail and the CRO within 24 hours of receipt in the study-specific SAE report form.

6.2.1.8 Suspected Unexpected Serious Adverse Reactions

A suspected unexpected serious AR (SUSAR) is defined as any suspected AR to study treatment that is both serious and unexpected.

The event(s) must meet all of the following criteria:

- Suspected AR
- Serious
- Unexpected
- Assessed as at least possibly related to study treatment or if Investigator's causality assessment is missing or indeterminate

The Sponsor will promptly evaluate all SUSARs against cumulative product experience to identify and expeditiously communicate possible new safety findings to Investigators, IRBs/IECs, and applicable health authorities based on applicable legislation.

To determine reporting requirements for single AE cases, the Sponsor will assess the expectedness of these events using the IB.

Reporting requirements will also be based on the Investigator's assessment of causality and seriousness, with allowance for upgrading by the Sponsor as needed.

6.2.1.9 Assessment of Severity

The severity of all AEs should be graded according to the Common Terminology Criteria for Adverse Events (CTCAE) Version 5.0. These criteria can be found at https://ctep.cancer.gov/protocolDevelopment/electronic_applications/docs/CTCAE_v5_Quick_Reference_8.5x11.pdf#search=%22CTCAE%22. For those AE terms not listed in the CTCAE, the following grading system should be used:

- CTCAE Grade 1: Mild; asymptomatic or mild symptoms; clinical or diagnostic observations only; intervention not indicated

- CTCAE Grade 2: Moderate; minimal local or noninvasive intervention indicated; limiting age appropriate instrumental activities of daily living

- CTCAE Grade 3: Severe or medically significant but not immediately life-threatening; hospitalization or prolongation of hospitalization indicated; disabling; limiting self-care activities of daily living
- CTCAE Grade 4: Life-threatening consequences; urgent intervention indicated
- CTCAE Grade 5: Death related to the AE.

Changes in the severity of an AE should be documented to allow an assessment of the duration of the event at each level of intensity to be performed. Adverse events characterized as intermittent do not require documentation of onset and duration of each episode.

6.2.1.10 Assessment of Causality

The Investigator's assessment of an AE's relationship to investigational product is part of the documentation process, but it is not a factor in determining what is or is not reported in the study. If there is any doubt as to whether a clinical observation is an AE, the event should be reported.

The relationship or association of the investigational product or the ICM administration of the investigational product in causing or contributing to the AE will be characterized using the following classification and criteria:

- **No** (unrelated, not related, unlikely to be related) – The time course between the administration of investigational product and the occurrence or worsening of the AE rules out a causal relationship and another cause (concomitant drugs, therapies, complications, etc.) is suspected.
- **Yes** (possibly, probably, or definitely related) – The time course between the administration of the investigational product and the occurrence or worsening of the AE is consistent with a causal relationship should be considered at least possibly related to investigational product or investigational product administration procedure. This is defined as all AEs that begin during or within 1 month following investigational product administration, plus all AEs regarded by the Investigator or Sponsor as at least possibly related to investigational product or investigational product administration procedure, plus all AEs for which the Investigator's causality assessment is missing or indeterminate.

6.2.1.11 Follow-Up of Patients Reporting Adverse Events

All AEs must be reported in detail on the appropriate page in the eCRF and followed to satisfactory resolution, until the Investigator deems the event to be chronic or not clinically significant, or until the patient is considered to be stable.

6.2.2 Risk Mitigation Plan

PR006A is not approved and clinical development is ongoing. The anticipated important safety risks for PR006A are provided in this section. Several measures will be taken to ensure the safety of patients participating in this study; patients with anticipated risks from study procedures are excluded while determining eligibility. Patients will undergo frequent safety monitoring during the study, including assessment of the nature, frequency, and severity of AEs. Patient safety will also be reviewed by an iDMC who will provide recommendations regarding further dosing. In addition, guidelines to manage AEs are provided in ensuing sections.

6.2.2.1 Mitigation of Risks Associated with Lumbar Puncture

Lumbar punctures performed for obtaining CSF for biomarker and safety assessments will be performed with care to minimize AEs. Cerebrospinal fluid leakage is more likely with large bore needles. The use of narrow-gauge needles and adherence to the guidelines in the study Procedure Manual will be implemented. Lumbar punctures may be performed under fluoroscopy guidance if this is requested by the local standard of care and/or if clinically indicated as per the Investigator's judgment.

Post-LP headaches sometimes occur within days after the procedure. If a post-LP headache develops, the patient should be encouraged to sit or preferably lie down in a comfortable position. Supportive treatment may include rehydration and consumption of caffeinated drinks, over-the-counter analgesics, and antiemetics. If these conservative measures fail, more specific measures may be indicated including use of a blood patch.

Post-LP spinal hematoma is a very rare but potentially serious complication that can present as persistent back pain, radicular pain, new sensory or motor symptoms, sphincter disturbance, or meningism. Prompt MRI of the lumbar spine should be performed if suspicion of spinal hematoma arises. Patients with susceptibility to bleeding are at an increased risk of spinal hematoma; as such, patients with coagulation abnormalities are

excluded from the study. Patients on blood thinners within the 2 weeks prior to Screening or who are anticipated to require initiation of blood thinners during the study are also excluded, as are patients who are on antiplatelet medications (prophylactic aspirin, clopidogrel) and are medically unable to temporarily halt the use of these drugs from at least 7 days prior to and at least 48 hours after intracisternal injection and LP. Management of spinal hematomas should be in consultation with neurosurgical professionals.

Meningitis is a rare potential risk of LP. Patients may present with headache, meningism, photophobia, neck stiffness, and pyrexia. If meningitis is suspected, a thorough meningitis workup, consultation with appropriate specialists, and prompt use of antibiotics should be considered urgently.

6.2.2.2 Risks Associated with the Use of Immunosuppressants

One loading dose of 6 mg oral sirolimus will be administered at Day -1, as well as a single dose of 1 g IV methylprednisone at Day 0. A concomitant maintenance dose of 2 mg/day sirolimus will be administered orally for 90 days starting at Day 0 which will then be tapered over the ensuing 15 to 30 days. The maintenance dose is administered to achieve trough sirolimus concentration of 4 ng/mL (range 2 to 8 ng/mL). One day after treatment with PR006A, patients will start prednisone 30 mg orally for 14 days, followed by a 7-day taper. At the Investigator's discretion, adjustments of dose and duration are permitted. Corticosteroids are administered for prophylactic suppression of inflammatory responses and to improve viral transduction. Despite the beneficial effects of prednisone and sirolimus in the prevention and management of potential liver enzyme abnormalities, systemic immunosuppressants in this dose range can produce a variety of AEs ranging from mild irritability to life-threatening or even fatal biochemical effects including but not limited to impaired wound healing, interstitial lung disease, hyperglycemia, hypertension, lymphomas/malignancies, hyperlipidemia, decline in renal function, proteinuria, embryo-fetal toxicity, and male fertility. While patients will be excluded if they are at increased risk of AEs, if corticosteroid side effects emerge, corticosteroids will be stopped or tapered as medically indicated. Glucose level checks, HbA1c, fructosamine, blood pressure, body weight, cholesterol, urine protein, lipids, red blood cell counts, neutrophil counts, protein glucose, CSF cell counts, CSF protein levels, CSF glucose levels, sirolimus trough concentrations, BMI, waist circumference, and psychosis incidence/aggravation will be monitored at study visits occurring during corticosteroid usage and after prednisone

discontinuation. The iDMC will assess safety variables as listed for the primary safety endpoints (Section 2) and as detailed in Section 11.1.1. Patients must have evidence of a negative MTB test and pneumococcal pneumonia and shingles vaccination coverage (within 10 years prior to Screening; allowed during Screening Period but at least 4 weeks prior to sirolimus loading dose) in order to initiate prednisone or sirolimus. Additionally, if medically indicated and as per local standard of care, Principal Investigators may consider performing EBV, CMV, and JC virus testing as well anti-viral (e.g., valganciclovir) and/or antibacterial (e.g., low-dose Bactrim) prophylaxis during sirolimus course.

If steroid or sirolimus side effects emerge, these drugs will be stopped or tapered as medically indicated. Details regarding additional monitoring for corticosteroid and sirolimus-related events are provided in Section 5.5.1.3

If clinically indicated and/or as per local practice, concomitant gastroprotective prophylactic medication, as well as potassium and/or calcium/vitamin D_3 and bisphosphonate supplementation, may be initiated. Specifically, for patients with pre-existing osteoporosis (without history of vertebral fracture in the past year) or history of fragility fracture, the Investigator should consider prescribing appropriate therapy intended to prevent/attenuate glucocorticoid-induced osteoporosis starting at the time of initiation of prednisone administration. The same consideration to provide therapy to prevent/attenuate glucocorticoid induced osteoporosis should be given to patients whose prednisone treatment may be extended beyond 6 weeks. A dual-energy x-ray absorptiometry (DEXA) scan will be performed at Screening and will be repeated at Month 12 for patients with baseline osteopenia or osteoporosis (Section 6.16). For patients receiving prednisone for more than 3 months, HbA1c will be measured at the end of prednisone therapy.

Continued participation in the study will not be affected if the patient or Investigator finds corticosteroid administration to be intolerable or the patient develops clinically significant AEs requiring early prednisone or sirolimus discontinuation.

The following TEAEs, which may be associated with immunosuppressant regimen (see above text), should be explicitly monitored:

- Peptic ulcer disease
- Erosive gastritis

- Osteoporosis
- Increase in body weight ≥5% from baseline
- BMI >25 or >30
- Increase in waist circumference ≥15% from baseline
- Grade ≥3 infections
- Incidence or aggravation of psychosis
- Hyperlipidemia/hypercholesterolemia
- Renal insufficiency
- Opportunistic infections (including progressive multifocal leukoencephalopathy (PML) and BK virus-associated nephropathy)
- Proteinuria
- Anemia
- Severe neutropenia

6.2.2.3 Risks Associated with Suboccipital Injection into the Cisterna Magna

Risks associated with injection into the cisterna magna include local discomfort, CSF leakage resulting in headache, hematoma, meningitis, and puncture of neuronal tissue.

Intracisternal administration will be performed by an interventional radiologist or neurosurgeon who has experience with the procedure. Investigational product administration will be performed only after MRA and brain MRI confirm that there are no structural contraindications or blood vessels in the area of injection and that there is sufficient CSF space in the ICM to safety administer investigational product. The procedure will be performed with the patient under general anesthesia or deep sedation and using imaging guidance in a fully equipped interventional or procedure room. Patients will be medically cleared by the anesthesiologist prior to the procedure. Following the procedure, patients will be monitored for at least 24 hours in an inpatient setting until fully recovered, prior to being discharged from the clinic after completion of the visit assessments. A follow-up safety MRI

will be performed approximately 1 month after the procedure to monitor for any subclinical neurological signs.

6.2.2.4 Risks Associated with AAV9-Based Therapy

The viral capsid of AAV9 may trigger the innate and adaptive immune system. Overall, products (investigational and marketed) using the AAV9 vector have been safe and generally well tolerated in over 500 patients. Out of an estimated 21 clinical trials investigating AAV9-based gene therapies, 7 use an IT route of administration with approximately 60 patients having received an investigational therapy. The mode of administration via CSF is intended to minimize systemic exposure, but inevitably there will be some escape of virus out of the CSF into the blood. In clinical studies, gene therapy candidates using AAV9 have been generally well tolerated. Transient elevations in AST and/or ALT, which respond to corticosteroid treatment, have been reported (Mendell et al 2017).

Pleocytosis and increased protein in the CSF was observed in one patient dosed with PR001A (Prevail's AAV9-based gene therapy candidate delivering the GBA1 gene to subjects suffering from PD-GBA). Steroid-responsive CSF pleocytosis has been reported in another gene therapy trial utilizing AAV9 viral vector to deliver GAN gene to the patients suffering from giant axonal neuropathy (NCT02362438) (Bharucha-Goebel et al, 2020).

To mitigate the risk of potential capsid and other related immune reactions, the prophylactic course of immunosuppressive treatment (consisting of corticosteroids and sirolimus) should be followed as specified in the protocol. In cases of elevations of liver enzymes, inflammatory changes in the CSF or other suspected immune reactions to PR006A, higher doses, prolongation or re-initiation of immunosuppressive treatment may be considered at the discretion of the principal investigator. Risk mitigation for the immunosuppressive regimen is discussed in Section 6.2.2.2.

While minimal axonal degeneration and gliosis limited to the dorsal funiculus as well as minimally increased cellularity in small clusters occurred in the DRG of few PR006A-treated NHPs, these findings were non-inflammatory, limited in their extent, and without clinical correlate, and therefore deemed non-adverse (PRV-2018-028). It is not predicted that the non-adverse findings in the NHPs will clinically translate to human patients. Notwithstanding, the protocol will exclude individuals with clinical evidence of peripheral symmetric sensory polyneuropathy and incidence of sensory symptoms will be monitored

during the study using the TNAS. If sensory symptoms appear after PR006A dosing, consultation with a neurologist with expertise in peripheral nerve disease should be considered along with additional corticosteroid use and or prolongation of prednisone or sirolimus taper.

6.2.2.5 Risks Associated with *GRN* Expression

There has been no previous experience with administration of a vector in humans aimed at expression of *GRN*. Since FTD-GRN patients are heterozygous mutation carriers and express a certain amount of normal PGRN, the potential risk to develop an immune response to the "wild type" PGRN produced by the transduced cells may be considered as null. In addition, prophylactic corticosteroids will be administered to mitigate the risk of an immune response.

Progranulin is a secreted glycoprotein associated with the regulation of lysosome function and inflammation (Chitramuthu et al 2017). Progranulin is broadly expressed in the CNS and periphery in a variety of cell types (Chitramuthu et al 2017). Progranulin expression has been reported to be upregulated in certain tumors and tumor cell lines (Serrero 2003; Chitramuthu et al 2017). Furthermore, overexpression of PGRN in vitro, in tumor cell lines or primary tumor cells, has been reported to increase certain tumorigenic properties in some contexts (He et al 2002; Chitramuthu et al 2017) but not in other contexts (Matsumura et al 2006). In contrast to these studies, overexpression of PGRN in vivo has been reported by several groups using AAV or lentiviral transduction and transgenic mouse approaches, without findings of tumor formation (Tao et al 2012; Altmann et al 2016; Arrant et al 2018). Furthermore, direct intravenous administration of PGRN in vivo has not been reported to be associated with tumorigenesis (Tang et al 2011). Consistent with these findings, the Sponsor's in vivo studies using PR006A to overexpress PGRN in mouse models or normal NHPs have not revealed tumorigenic potential.

Based on the above analysis, an increased risk of malignancy is considered by the Sponsor to be unlikely in the context of PR006A administration. Patients with a history of cancer within 5 years of Screening or current presence of pre-cancerous lesions will be excluded from study participation, with the exception of fully excised prostate carcinoma in situ and fully excised nonmelanoma skin cancers that have been stable for at least 6 months. Patients will also be required to have up to date age- and gender-appropriate cancer screenings, completed as per the Investigator's judgment and local standard of care, prior to inclusion/exclusion screening. Additionally, the occurrence of malignancy at any point after gene transfer that is

judged as at least probably related to PR006A per the Sponsor's final causality assessment is one of the study stopping rules (Section 4.2.4).

6.2.2.6 Risks Associated with the Presence of Antibodies to AAV9

No known safety risks have been described in patients seropositive to AAV9; however, in case of the presence of antibodies to AAV9, there is a risk of reduced efficacy of AAV9-based gene therapy. This risk is mitigated by the ICM route of administration of PR006A. Baseline titers of AAV9 antibodies will be assessed in study patients; however, seropositive patients will not be screen failed.

6.2.2.7 Risks Associated with Other Study Procedures

The risks associated with other study procedures, including MRA, MRI, general anesthesia or deep sedation, and blood sample collection are described in the ICF.

6.3 Safety Monitoring Committee

The safety of the study will be monitored by an iDMC, as described in Section 11.1.1.

6.4 Pregnancy

Pregnancy is not regarded as an AE unless there is a suspicion that the investigational product may have interfered with the effectiveness of a contraceptive medication. Any pregnancy that occurs during study participation must be reported using the same procedures as an SAE (Section 6.2.1.6). To ensure patient safety, each pregnancy must be reported within 24 hours of learning of its occurrence. The Investigator will continue to follow patients (or partners of male patients) who become pregnant under the current protocol and will collect information on the pregnancy complications as well as on potential birth abnormalities and/or defects (including spontaneous miscarriage, elective termination, normal birth, or congenital abnormality) and status of mother and child, even if the patient discontinued from the study. Pregnancy complications and elective terminations for medical reasons should be reported as an AE or SAE. Spontaneous miscarriages must be reported as an SAE.

Any SAE occurring in association with a pregnancy, brought to the Investigator's attention after the patient has completed the study, and considered by the Investigator as possibly related to the study treatment must be promptly reported.

6.5 Laboratory Analyses

The laboratory parameters will be tested per the Schedules of Events (Table 13-1 and Table 13-2):

GRN Sequencing

At Screening, patients that meet all criteria will be tested for full *GRN* sequencing by the central laboratory. Documentation of prior testing demonstrating the presence of at least 1 pathogenic *GRN* mutation from a Clinical Laboratory Improvement Amendments (CLIA)-approved laboratory or an ex-US CLIA-equivalent certified laboratory that is verified by the Investigator is allowed so the patient can continue Screening without waiting for central laboratory results. However, central laboratory confirmation of *GRN* is required prior to investigational product administration. Results from the external laboratory should be filed in the patient's medical record.

Standard Safety Chemistry Panel[1]

Alanine aminotransferase	Albumin
Alkaline phosphatase	Amylase
Aspartate aminotransferase	Bicarbonate
Blood urea nitrogen	Calcium
Chloride	Creatine kinase
Creatinine	Estimated glomerular filtration rate
Gamma-glutamyl transferase	Glucose
Inorganic phosphorus	Lactate dehydrogenase
Lipase	Potassium
Sodium	Total bilirubin
Total protein	Uric acid

1. During Screening and until the end of the corticosteroid taper, blood chemistry will be performed fasting.

Additional Chemistry Parameters[1]

HbA1c	Fructosamine
Lipid panel (total cholesterol, high-density lipoprotein cholesterol, low-density lipoprotein cholesterol, total triglycerides)	

1. During Screening and until the end of the corticosteroid taper, blood chemistry will be performed fasting.

Immunogenicity Assays

Anti-AAV9 antibody<br>Anti-PGRN antibody	AAV9 Enzyme-linked immunospot (ELISpot)<br>PGRN ELISpot

Endocrinology

Follicle-stimulating hormone[1]	Free thyroxine (T4)
TSH	

1. A follicle-stimulating hormone test will be performed at Screening for women who are post-menopausal with spontaneous amenorrhea for at least 2 years, to confirm their post-menopausal status at Screening.

Sirolimus Monitoring

Sirolimus trough concentrations

Hematology

Hematocrit	Hemoglobin
Platelets	Red blood cell count
White blood cell count and differential[1]	

1. Manual microscopic review is performed only if white blood cell count and/or differential values are out of reference range.

Coagulation

International normalized ratio	Activated partial thromboplastin time

Urinalysis

Bilirubin	Blood
Glucose	Ketones
Leukocyte esterase	Microscopy[1]
Nitrite	pH
Protein	Specific gravity
Urobilinogen	

1. Microscopy is performed only as needed based on positive dipstick test results.

Serology

hepatitis B surface antigen	hepatitis C antibody
HIV 1/2	MTB test

Drug Screening

Opiates	Benzodiazepines
Amphetamines	Cocaine
Barbiturates	Phencyclidine

Pregnancy Testing

Serum β-human chorionic gonadotropin[1]	Urine pregnancy[1]

1. A serum pregnancy test will be performed at Screening and for any positive urine pregnancy tests. Urine pregnancy tests will be performed at Day -1 or Day 0 (before investigational product administration), and every 3 months for the first 12 months, for women of childbearing potential.

Additional blood or urine samples may be taken at the discretion of the Investigator if the results of any test fall outside the reference ranges, or clinical symptoms necessitate additional testing to monitor patient safety. Where the clinical significance of abnormal laboratory results is considered uncertain, Screening laboratory tests may be repeated per discretion of the Investigator before dosing to confirm eligibility. If there is an alternative explanation for a positive urine or blood test for drugs of abuse (e.g., previous occasional intake of a medication or food containing for example codeine, or opiates), the test may be repeated to confirm washout.

After dosing, in the event of unexplained abnormal clinically significant laboratory test values, the tests should be repeated and followed up until they have returned to the normal range and/or an adequate explanation of the abnormality is found.

Any abnormal laboratory test results (hematology, blood chemistry, or urinalysis) or other safety assessments (e.g., ECGs, radiological scans, vital sign measurements), including those that worsen from baseline, thought to be clinically significant in the medical and scientific judgment of the Investigator are to be recorded as AEs or SAEs. All events (occurring after Screening) of ALT or AST >3 × ULN and bilirubin >2 × ULN (>35% direct bilirubin) or ALT or AST >3 × ULN and international normalized ratio >1.5, which may indicate severe liver injury (possible Hy's Law), must be reported as an SAE.

However, any clinically significant safety assessments that are associated with the underlying disease, unless judged by the Investigator to be more severe than expected for the patient's condition, are **not** to be reported as AEs or SAEs.

6.6 Antibody/Biomarker Evaluation in Blood

Anti-AAV9 antibodies, anti-PGRN antibodies, AAV9 ELISpot, PGRN ELISpot, PGRN, NfL, and biomarkers of neuroinflammation, astroglial pathology, and lysosomal function will be measured in blood.

6.7 Biomarker Evaluation in Urine

Biomarkers of lysosomal function (e.g., BMP) will be measured in urine.

6.8 Cerebrospinal Fluid Evaluations

Anti-AAV9 antibodies, anti-PGRN antibodies, biomarkers of neurodegeneration ([redacted]), PGRN, biomarkers of neuroinflammation, astroglial pathology, and lysosomal function [redacted] as well as local assessment of cell count, protein, and glucose will be measured in CSF.

The LP and CSF collection will occur during the treatment period (Day 0; Table 13-1), with optional additional CSF collection during follow-up (Table 13-2). Patients are not required to participate in any LP procedures and CSF collections during follow-up in order to participate in the study. Patients who agree to participate in additional CSF sampling will be required to provide an additional consent (contained in the main ICF).

6.9 Sampling for Future Biomedical Research

Blood, urine, and CSF samples, including residual samples already scheduled, will be collected for future research purposes to examine and/or research potential disease modifiers and/or biomarkers, including genetic parameters.

Patients are not required to participate in future biomedical research in order to participate in the study. Patients who agree to participate in this sampling will be required to provide an additional consent (contained in the main ICF).

6.10 Vital Signs

Vital signs will be taken after a 5-minute seated rest and will include orthostatic blood pressure, pulse rate, respiratory rate, and oral or axillary body temperature. Vital sign measurements should be performed prior to any blood draws, whenever possible.

6.11 Electrocardiograms

The 12-lead ECGs will be performed after a 10-minute supine rest and will include heart rate, QRS, and QT interval and will be read locally. The 12-lead ECGs should be performed prior to any blood draws, whenever possible. The Screening 12-lead ECG should be performed in triplicate.

6.12 Physical Examinations

A full physical examination will be performed at Screening and Month 2. Brief symptom-based physical examinations will be performed at all other time points, unless a full physical examination is deemed necessary by the Investigator. New or worsened clinically significant abnormal findings will be recorded as AEs/SAEs

6.13 Height, Weight, Body Mass Index, and Waist Circumference

Height will be measured at Screening only. Weight and waist circumference will be measured and BMI calculated during Screening, Month 2, and every 3 months post-administration of PR006A through Month 12. New or worsened clinically significant abnormal findings will be recorded as AEs/SAEs.

Waist circumference measurement should be made at the approximate midpoint between the lower margin of the last palpable rib and the top of the iliac crest. The tape should be snug around the body, but not pulled so tight that it is constricting. The mean of 2 measurements will be recorded.

6.14 Neurological Examinations

A full neurological examination will be performed at Screening and Month 2. Brief symptom-based neurological examinations will be performed at all other time points, unless a full neurological examination is deemed necessary by the Investigator. Symptom-driven evaluations may be performed at any time. New or worsened clinically significant abnormal findings will be recorded as AEs/SAEs.

6.15 Magnetic Resonance Imaging and Magnetic Resonance Angiography

Brain MRA and brain MRI will be performed at Screening to evaluate eligibility and to determine whether the ICM procedure is safe for the patient. Screening MRIs will be read locally and centrally; MRAs will only be read locally. Post-baseline brain MRIs for safety evaluation will be performed without contrast unless specifically requested by the Investigator.

An MRI may be performed at any visit for safety monitoring if clinically indicated, as determined by the Investigator. After the 1-month safety MRI, follow-up MRIs may be done within a window of ±7 days in order to give flexibility to patients and sites with scheduling. New or worsened clinically significant abnormal findings will be recorded as AEs/SAEs.

6.16 Dual-Energy X-Ray Absorptiometry

DEXA scans of the hip and spine will be performed at Screening and at Month 12 only in patients with known medical history of osteoporosis or osteopenia or with multiple risk factors (in addition to the planned exposure to the corticosteroid course) for osteoporosis. Abnormal Month 12 findings will be recorded as AEs/SAEs.

6.17 Columbia Suicide Severity Rating Scale

Consistent with regulatory guidance, the potential occurrence of suicide-related ideation and behaviors will be assessed in this Phase 1/2 study using the C-SSRS during Screening (C-SSRS baseline) and at the time points specified after Screening (C-SSRS since last visit).

The C-SSRS (Posner et al 2010) is a measure of the spectrum of suicidal ideation and behavior and was developed in the National Institute of Mental Health Treatment of Adolescent Suicide Attempters Study to assess severity and track suicidal events through any treatment. The C-SSRS consists of a clinical interview that can be administered during any evaluation or risk assessment to identify the occurrence and intensity of suicidal thoughts and suicidal behaviors. It can also be used during treatment to monitor for clinical worsening. If a suicide-related thought or behavior is identified at any time during the study, a thorough evaluation will be performed by a study physician and appropriate medical care will be provided (DHSS, 2012); the event will be reported as an AE or SAE.

Data from the C-SSRS will be included as part of the ongoing monitoring by the iDMC.

6.18 Treatment-Induced Peripheral Neuropathy Assessment Scale

The TNAS is a 9-item patient-reported outcome measure that rates symptom burden associated with treatment-induced peripheral neuropathy (TIPN). Patients are first asked to assess the severity of five common symptoms of TIPN as experienced in their extremities in the past 24 hours on an 11-point Likert scale ranging from “Not present” to “As bad as you can imagine”; symptoms assessed include numbness, tingling, pain, hot or burning sensations, and coldness. Patients are also asked to rate 4 items assessing the worst level of interference experienced in the last 24 hours caused by symptoms of TIPN: Difficulty using hands or fingers, trouble walking, trouble with balance or falling, and disturbed sleep; these 4 items are rated on an 11-point Likert scale ranging from “Have not interfered” to “Interfered completely.” (Mendoza et al 2020).

7 Statistical and Analytical Plan

A statistical analysis plan (SAP) will be prepared to provide additional details on the approach to analyze and display the data. The SAP will be finalized before the database is locked and prior to the formal interim and final analysis.

7.1 Primary Endpoints

7.1.1 Primary Safety Endpoints

The following primary safety endpoints are measured up to 5 years:

- Incidence and severity of TEAEs and SAEs, including clinically significant changes in vital signs, clinical laboratory assessments, C-SSRS, and TNAS; treatment-emergent transaminitis; treatment-emergent diabetes and prediabetes; treatment-emergent peptic ulcer disease and/or erosive gastritis; treatment-emergent osteoporosis; treatment-emergent increase in body weight ≥5% from baseline, treatment-emergent BMI >25 or >30, and treatment-emergent increase in waist circumference ≥15% from baseline; incidence of Grade ≥3 infections; incidence/aggravation of psychosis; treatment-emergent hypercholesterolemia or hyperlipidemia; treatment-emergent interstitial lung disease; treatment-emergent opportunistic infections (including progressive multifocal leukoencephalopathy (PML) and BK virus-associated nephropathy); sirolimus trough levels; 12-lead ECGs; brain MRIs; physical examinations; and neurological examinations
- Sum of ARs and suspected ARs
- Sum of serious ARs and serious suspected ARs
- Incidence of procedure or treatment-emergent safety findings as per brain MRI.
- Change from baseline in immunogenicity of AAV9 and PGRN in blood at Day 14 and Months 1, 2, 3, 6, 9, and 12 and in CSF at Months 2 and 12.

7.1.2 Primary Efficacy Endpoints

The following are the primary efficacy endpoints:

- Change from baseline in PGRN levels in blood at Months 1, 2, 3, 6, 9, and 12
- Change from baseline in PGRN levels in CSF at Months 2 and 12.

7.2 Secondary Endpoints

The following secondary endpoints, consisting of efficacy and safety measures will be measured from baseline to Year 1 and will be summarized by assessment time. Corresponding changes from baseline will also be summarized for these measures.

7.2.1 Secondary Efficacy Endpoints

- Change from baseline in CDR plus NACC FTLD at Months 6 and 12
- Change from baseline in NfL levels in blood at Months 2, 6, 9, and 12
- Change from baseline in NfL levels in CSF at Months 2 and 12

[redacted] [redacted]

[redacted]

[redacted]

- [redacted]
- [redacted]
- [redacted]
- [redacted]
- [redacted]
- [redacted]
- [redacted]
- [redacted]

- [redacted]
- [redacted]

[redacted]

[redacted]

- [redacted]
- [redacted]
- [redacted]
- [redacted]
- [redacted]
- [redacted]
- [redacted]
- [redacted]
- [redacted]
- [redacted]
- [redacted]
- [redacted]
- [redacted]
- [redacted]

7.4 Sample Size Calculations

Fifteen patients ([redacted] cohorts of 5 patients each) will be administered a one-time dose of PR006A (low, mid, [redacted] dose). The sample size is based on enrollment feasibility and clinical objectives rather than statistical rationale. The sample size chosen of 5 patients for the low-, mid-, [redacted] dose cohorts is considered to be sufficient for evaluation of the safety, tolerability, and immunogenicity of PR006A in each cohort. The sample size is not based on statistical power considerations.

7.5 Analysis Sets

The following analysis sets will be used in the statistical analyses:

- Safety Analysis Set, which will include all enrolled patients who receive investigational product.
- Treatment Analysis Set, which will include all patients in the Safety Analysis Set and who have at least 1 efficacy evaluation following the Baseline visit.

Patient information, safety, efficacy, and biomarker results will be summarized descriptively. No inferential analysis will be performed.

7.6 Description of Subgroups to be Analyzed

Subgroup analyses by age, sex, race, and phenotypic presentation of symptoms (bvFTD, PPA-FTD, FTD with corticobasal syndrome, or a combination of syndromes) will be reported.

7.7 Statistical Analysis Methodology

Statistical analysis will be performed using SAS software Version 9.4 or later. Continuous variables will be summarized using the mean, the standard deviation, median, minimum value, and maximum value. Categorical variables will be summarized using frequency counts and percentages. Data will be listed in data listings.

Details of the statistical analyses, methods, and data conventions are described in the SAP.

No formal significance testing will be performed.

7.7.1 Analysis of Primary Endpoints

The primary endpoints (presented in Section 7.1) will be summarized with descriptive statistics, at all time points where these variables are collected. No inferential statistical tests will be performed using safety data. The Safety Analysis Set will be used for analyses of the safety endpoints. The Treatment Analysis Set will be used for analyses of the primary efficacy endpoints.

7.7.2 Analysis of Secondary Endpoints

7.7.2.1 Analysis of Secondary Efficacy Endpoints

The secondary endpoints, efficacy measures (presented in Section 7.2.1) from baseline to Year 1, will be summarized by assessment time. Corresponding changes from baseline will also be summarized for these measures. The Treatment Analysis Set will be used for analyses of the secondary efficacy endpoints.

7.7.2.2 Analysis of Secondary Safety Endpoints

The secondary safety endpoints, safety measures (presented in Section 7.2) from baseline to Year 1, will be summarized by assessment time. Corresponding changes from baseline will also be summarized for these measures. The Safety Analysis Set will be used for analyses of the secondary safety endpoints.

7.7.3 Analyses of Exploratory Endpoints

7.7.3.1 Exploratory Efficacy Endpoints (Baseline to Year 1)

The exploratory efficacy measures for the period between baseline and Year 1 (presented in Section 7.3.1) will be summarized by assessment time. Corresponding changes from baseline will also be summarized for these efficacy endpoints.

7.7.3.2 Exploratory Efficacy and Safety Endpoints (Post-Year 1)

The exploratory efficacy endpoints for the post-Year 1 period (presented in Section 7.3.2) will be summarized by assessment time. Corresponding changes from baseline will also be summarized for these efficacy and safety measures.

7.7.4 Other Analyses

Demographics, medical history, physical examination, social history, and risk factor variables at baseline will be summarized descriptively.

7.7.5 Interim Analysis

To facilitate the ongoing review of efficacy biomarker data (e.g., PGRN in blood and CSF), informal interim analyses may be performed at regular intervals based on Sponsor's discretion.

The Sponsor will perform 3 formal interim analyses: 1) after all patients complete 12 months of treatment in Cohort 1; 2) after all patients complete 12 months of treatment in Cohort 2; [redacted]. The SAP and all patient data for each cohort will be finalized prior to each formal interim analysis. During the dose-escalation period of the study, the Sponsor will perform a review of biomarker data at regular intervals.

Additional interim analyses may be performed during the follow-up period (post-Year 1) based on Sponsor's discretion.

8 Data Quality Assurance

This study will be conducted according to the International Council for Harmonisation (ICH) E6(R2) risk and quality processes described in the applicable procedural documents. The quality management approach to be implemented in this study will be documented and will comply with the current ICH guidance on quality and risk management.

8.1 Data Management

As part of the responsibilities assumed by participating in the study, the Investigator agrees to maintain adequate case histories for the patients treated as part of the research under this protocol. The Investigator agrees to maintain accurate eCRFs and source documentation as part of the case histories. These source documents may include diary cards/laboratory reports/ECG strips/etc.

Study site personnel will enter patient data into Medidata RAVE® (the eCRF program). The analysis data sets will be a combination of these data and data from other sources (e.g., laboratory data).

Clinical data management will be performed in accordance with applicable Prevail Therapeutics, Inc. (Prevail) standards and CRO data cleaning procedures to ensure the integrity of the data, e.g., removing errors and inconsistencies in the data. Adverse event terms will be coded using the MedDRA, an internal validated medical dictionary, and concomitant medications will be coded using the World Health Organization Drug Dictionary (WHODrug).

After database lock, each study site will receive a compact disc, read-only memory (CDROM) containing all of their site-specific eCRF data as entered into the Medidata RAVE system for the study, including full discrepancy and audit history. Additionally, a CDROM copy of all of the study site's data from the study will be created and sent to the Sponsor for storage. The CRO will maintain a duplicate CDROM copy for their records. In all cases, patient initials will not be collected or transmitted to the Sponsor.

9 Ethics

9.1 Independent Ethics Committee or Institutional Review Board

Federal regulations and the ICH guidelines require that approval be obtained from an IRB/IEC before participation of human patients in research studies. Before study onset, the protocol, informed consent, advertisements to be used for the recruitment of study patients, and any other written information regarding this study to be provided to the patient or the patient's study partner must be approved by the Sponsor and by the IRB/IEC. Documentation of all IRB/IEC approvals and of the IRB/IEC compliance with ICH harmonised tripartite guideline E6(R2): GCP will be maintained by the site and will be available for review by the Sponsor or its designee.

All IRB/IEC approvals should be signed by the IRB/IEC chairman or designee and must identify the IRB/IEC name and address, the clinical protocol by title or protocol number or both, and the date approval or a favorable opinion was granted.

The Investigator is responsible for providing written summaries of the progress and status of the study at intervals not exceeding 1 year or otherwise specified by the IRB/IEC. The Investigator must promptly supply the Sponsor or its designee, the IRB/IEC, and, where applicable, the institution, with written reports on any changes significantly affecting the conduct of the study or increasing the risk to patients.

9.2 Ethical Conduct of the Study

The study will be performed in accordance with the ethical principles that have their origin in the Declaration of Helsinki, ICH GCP, the protocol, and all applicable regulations.

9.3 Patient Information and Consent

Written informed consents approved by the Sponsor and by the IRB/IEC in compliance with regulatory authority regulations including US Title 21 Code of Federal Regulations (CFR) Part 50 shall be obtained from each patient and their study partner before entering the study. Informed consent templates may be provided by the Sponsor to investigative sites. If any institution-specific modifications to study-related procedures are proposed or made by the site, the consents should be reviewed by the Sponsor or its designee or both before IRB/IEC submission. Once reviewed, the consent will be submitted by the Investigator to his or her

IRB/IEC for review and approval before the start of the study. If the ICFs are revised during the course of the study, all active participating patients and/or their study partners must sign the revised form.

Before recruitment and enrollment, each prospective patient or his/her study partner will be given a full explanation of the study and be allowed to read the approved ICFs. Once the Investigator is assured that the patient and study partner understand the implications of participating in the study, the patient and study partner will be asked to give consent to participate in the study by signing and dating the ICFs.

The Investigator shall retain the signed, dated original ICFs and give a copy of the signed, dated original form to the patient or study partner.

9.4 Consent for Sampling for Future Biomedical Research

Patients willing to provide blood, urine, and CSF samples for future biomedical research will be required to provide an additional consent (contained in the main ICF) signed by themselves or LAR that addresses the use of these samples as set forth in this protocol. The Investigator or authorized designee will explain to each patient or their LAR the objectives of the future research. Patients will be informed that they are free to refuse to participate and may withdraw their consent at any time and for any reason during the storage period.

The optional consent language will describe that the samples retained may be utilized to identify or better understand putative, prognostic, and predictive markers associated with FTD-GRN and markers of therapeutic response to PR006A. Moreover, samples may be used to develop diagnostic and analytical tests. Background and longitudinal clinical disease characteristics (e.g., FTD clinical phenotype) and associated biomarker data may be utilized to predict subsequent disease progression patterns and identify predictors of response to treatment. Magnetic resonance imaging scans may also be stored for future analysis.

Samples and scans collected for future biomedical research may be analyzed by Sponsor staff and/or designated vendors/partners.

9.5 Patient Card

Upon enrollment in the study, the patient will receive a patient card to be carried at all times. The patient card will state that the patient is participating in a clinical research study, type of treatment, and contact details in case of an SAE.

10 Investigator's Obligations

The following administrative items are meant to guide the Investigator in the conduct of the study but may be subject to change based on industry and government standard operating procedures, working practice documents, or guidelines. Changes will be reported to the IRB/IEC but will not result in protocol amendments.

10.1 Confidentiality

All laboratory specimens, evaluation forms, reports, and other records will be identified in a manner designed to maintain patient confidentiality. All records will be kept in a secure storage area with limited access. Clinical information will not be released without the written permission of the patient (or the patient's legal guardian), except as necessary for monitoring and auditing by the Sponsor, its designee, the US FDA, or the IRB/IEC.

The Investigator and all employees and coworkers involved with this study may not disclose or use for any purpose other than performance of the study any data, record, or other unpublished, confidential information disclosed to those individuals for the purpose of the study. Prior written agreement from the Sponsor or its designee must be obtained for the disclosure of any said confidential information to other parties. Additional confidentiality requirements and obligations will be set forth in the clinical trial agreement (CTA) to be entered into by the Sponsor, the Investigator, and the institution.

10.2 Financial Disclosure and Obligations

Investigators are required to provide financial disclosure information to allow the Sponsor to submit the complete and accurate certification or disclosure statements required under 21 CFR 54. In addition, the Investigator must provide to the Sponsor a commitment to promptly update this information if any relevant changes occur during the course of the investigation and for 1 year following the completion of the study.

Neither the Sponsor nor the CRO is financially responsible for further testing or treatment of any medical condition that may be detected during the Screening process. In addition, in the absence of specific arrangements, neither the Sponsor nor the CRO is financially responsible for further treatment of the patient's disease.

10.3 Investigator Documentation

Prior to beginning the study, the Investigator will be asked to comply with ICH E6(R2) 8.2 and, for US sites, Title 21 of the CFR by providing the following essential documents, including but not limited to:

- IRB/IEC approval
- Original Investigator-signed Investigator agreement page of the protocol
- For US sites, Form FDA 1572 (or equivalent), fully executed, and all updates on a new fully executed Form FDA 1572 (or equivalent)
- For non-US sites, the Investigator's current curriculum vitae and/or other documentation evidencing qualifications, and any other documents that the IRB/IEC may request. For US sites, a curriculum vitae for the Investigator and each subinvestigator listed on Form FDA 1572 (or equivalent)
- Financial disclosure information to allow the Sponsor to submit complete and accurate certification or disclosure statements required under 21 CFR 54. In addition, the Investigators must provide to the Sponsor a commitment to promptly update this information if any relevant changes occur during the course of the investigation and for 1 year after the completion of the study.
- IRB/IEC-approved informed consent, samples of site advertisements for recruitment for this study, and any other written information regarding this study that is to be provided to the patient or legal guardian, and
- Laboratory certifications and normal ranges for any local laboratories used by the site.

10.4 Study Conduct

The Investigator agrees that the study will be conducted according to the principles of ICH E6(R2). The Investigator will conduct all aspects of this study in accordance with all national, state, and local laws or regulations. Study information from this protocol will be posted on publicly available clinical trial registers before enrollment of patients begins.

10.5 Adherence to Protocol

The Investigator agrees to conduct the study as outlined in this protocol in accordance with ICH E6(R2) and all applicable guidelines and regulations.

10.6 Adverse Events and Study Report Requirements

By participating in this study, the Investigator agrees to submit reports of SAEs to the Sponsor and/or IRB/IEC according to the timeline and method outlined in the protocol. In addition, the Investigator agrees to submit annual reports to the study site IRB/IEC as appropriate.

10.7 Investigator's Final Report

Upon completion of the study, the Investigator, where applicable, should inform the institution; the Investigator/institution should provide the IRB/IEC with a summary of the study's outcome and the Sponsor and regulatory authority(ies) with any reports required.

10.8 Records Retention

Essential documents should be retained until at least 2 years after the last approval of a marketing application in an ICH region and until there are no pending or contemplated marketing applications in an ICH region or at least 2 years have elapsed since the formal discontinuation of clinical development of the investigational product. These documents should be retained for a longer period, however, if required by the applicable regulatory requirements or by an agreement with the Sponsor. It is the responsibility of the Sponsor to inform the Investigator/institution as to when these documents no longer need to be retained.

10.9 Publications

After completion of the study, the data may be considered for reporting at a scientific meeting or for publication in a scientific journal. In these cases, the Sponsor will be responsible for these activities and will work with the Investigators to determine how the manuscript is written and edited, the number and order of authors, the publication to which it will be submitted, and other related issues. The Sponsor has final approval authority over all such issues.

Data are the property of the Sponsor and cannot be published without prior authorization from the Sponsor, but data and publication thereof will not be unduly withheld. Further terms concerning publication will be set forth in the CTA entered into by the Sponsor, the Investigator, and the institution.

11 Study Management

The administrative structure will include an iDMC.

11.1 Monitoring

11.1.1 Independent Data Monitoring Committee

An iDMC consisting of clinicians and a biostatistician otherwise unaffiliated with the conduct of the study will conduct a review of the safety, tolerability, and immunogenicity data; and, to the extent available, efficacy data in accordance with the charter. Members of the iDMC will not be allowed to participate as Investigators in this study and must not have competing interests that could affect their roles with respect to the study. The iDMC will assess safety variables as listed for the primary safety endpoints outlined in Section 2. The iDMC may recommend protocol modification(s), including dose recommendations and study termination, based on their review of the nature, frequency, and/or severity of an AE(s) to ensure patient safety and the ethical continuation of the study. The study stopping rules are described in Section 4.2.4.

Enrollment will be staggered for the initial 2 patients receiving the low dose (3.5×10^{13} vg) of PR006A in Cohort 1, such that at least 8 weeks will separate dosing of each patient. This staggering will permit review of safety, tolerability, immunogenicity, and, to the extent available, efficacy results by the iDMC, who will ultimately provide a recommendation on further dosing.

At least 8 weeks after the second patient is dosed in Cohort 1, the iDMC will review all available safety, tolerability, immunogenicity, and to the extent feasible, efficacy results from the first 2 patients in this cohort (dosed in a staggered manner). At the recommendation of the iDMC, the Sponsor will decide whether to continue dosing the remaining 3 patients in Cohort 1 (low dose) without staggering and in parallel begin staggered enrollment into the mid-dose cohort (Cohort 2). A decision to dose escalate will be based on data from at least 2 patients from the concurrent cohort. However, all cohorts will enroll a minimum of 3 patients, unless enrollment is terminated due to safety concerns or based on biomarker review this dose is considered to be non-efficacious. In all other circumstances, the full cohort of 5 patients will be enrolled.

Cohort 2 will enroll 5 patients to receive the mid-dose (7.0×10^{13} vg) of PR006A[redacted]. The design for Cohorts 2 [redacted] enrollment is the same as for Cohort 1.

If a decision is made to not open or to limit enrollment into a cohort (i.e., Cohort 2 [redacted]) due to safety or tolerability reasons, the unenrolled subjects may be re-allocated to a lower dose level cohort.

Specific details regarding responsibilities and governance, including roles and responsibilities of each member and the Sponsor's study team, will be documented in the iDMC Charter.

11.1.2 Monitoring of the Study

The clinical monitor, as a representative of the Sponsor, has the obligation to follow the study according to the monitoring plan. In doing so, the monitor will visit the Investigator and study site at periodic intervals, in addition to maintaining necessary telephone and letter contact. The monitor will maintain current personal knowledge of the study through observation, review of study records and source documentation, and discussion of the conduct of the study with the Investigator and study site personnel.

All aspects of the study will be carefully monitored, by the Sponsor or its designee, for compliance with applicable government regulation with respect to current GCP and current standard operating procedures.

11.1.3 Inspection of Records

Investigators and institutions involved in the study will permit study-related monitoring, audits, IRB/IEC review, and regulatory inspections by providing direct access to all study records.

The Investigator should promptly notify the Sponsor and its representatives (as appropriate) of any inspections scheduled by any regulatory authorities and promptly inform them of the outcome, and forward copies of any inspection reports received and associated Investigator responses to the Sponsor.

11.2 Management of Protocol Amendments and Deviations

11.2.1 Modification of the Protocol

Any changes in this research activity, except those necessary to remove an apparent, immediate hazard to the patient, must be reviewed and approved by the Sponsor or its designee. Amendments to the protocol must be submitted in writing to the Investigator's IRB/IEC for approval before patients can be enrolled into an amended protocol. During emergent situations, such as the coronavirus disease 2019 (COVID-19) pandemic or other natural disaster(s), modification(s) may be necessary to protocol-specified procedures. Central to any decision should be ensuring that the safety of clinical trial participants can be maintained.

11.2.2 Protocol Deviations

A deviation from the protocol is an unintended or unanticipated departure from the procedures or processes approved by the Sponsor and the IRB/IEC and agreed to by the Investigator. A significant deviation occurs when there is nonadherence to the protocol by the patient or Investigator that results in a significant, additional risk to the patient. Significant deviations can include nonadherence to inclusion or exclusion criteria, or nonadherence to FDA regulations or ICH GCP guidelines, and may lead to the patient being withdrawn from the study (Section 4.2). Further terms concerning protocol deviations including reporting of protocol deviations will be set forth in the CTA entered into by the Sponsor, the Investigator, and the institution.

In the unlikely event that there is a deviation from the protocol, the Investigator or designee must document and explain in the patient's source documentation such deviation from the approved protocol. The Sponsor and IRB/IEC must be notified. The Investigator may implement a deviation from, or a change of, the protocol only to eliminate an immediate hazard to study patients without prior Sponsor or IRB/IEC approval. As soon as possible after such an occurrence, the implemented deviation or change, the reasons for it, and any proposed protocol amendments should be submitted to the IRB/IEC for review and approval, to the Sponsor for agreement, and to the regulatory authorities, if required.

Protocol deviations will be documented by the clinical monitor in the monitoring visit report throughout the course of monitoring visits. Investigators will be notified in writing by the

monitor of deviations. The IRB/IEC should be notified of all protocol deviations in a timely manner.

11.3 Study Termination

The Sponsor reserves the right to discontinue the study at any time for any reason.

The end of the study is defined as the date on which the last patient completes the last visit (includes follow-up visit).

11.4 Final Report

Whether the study is completed or prematurely terminated, the Sponsor will ensure that the clinical study reports are prepared and provided to the regulatory agencies as required by the applicable regulatory requirements. The Sponsor will also ensure that the clinical study reports in marketing applications meet the standards of the ICH harmonised tripartite guideline E3: Structure and content of clinical study reports.

Where required by applicable regulatory requirements, an Investigator signatory will be identified for the approval of the clinical study report. The Investigator will be provided reasonable access to statistical tables, figures, and relevant reports and will have the opportunity to review the complete study results.

Upon completion of the clinical study report, the Sponsor will provide the Investigator with the full summary of the study results. The Investigator is encouraged to share the summary results with the study patients, as appropriate. If required by applicable health authorities, the study results will be posted on publicly available clinical trial registers.

12 Reference List

Altmann C, Hardt S, Fischer C, et al. Progranulin overexpression in sensory neurons attenuates neuropathic pain in mice: role of autophagy. Neurobiol Dis. 2016;96:294-311.

Arrant AE, Onyilo VC, Unger DE, et al. Progranulin gene therapy improves lysosomal dysfunction and microglial pathology associated with frontotemporal dementia and neuronal ceroid lipofuscinosis. J Neurosci. 2018;38(9):2341-58.

Baker M, Mackenzie IR, Pickering-Brown SM, et al. Mutations in progranulin cause tau-negative frontotemporal dementia linked to chromosome 17. Nature. 2006;442(7105):916-9.

Bang J, Spina S, Miller BL. Frontotemporal dementia. Lancet. 2015;386(10004):1672-82.

Bharucha-Goebel, D, Saade D, Kang E, et al. A systematic analysis of the immunologic effects of intrathecal AAV9 mediated gene transfer targeting the nervous system in giant axonal neuropathy [abstract]. Mol Ther. 2020;28(4S1):24-25. Abstract no. 49.

Busner J, Targum SD. The Clinical Global Impressions Scale: applying a research tool in clinical practice. Psychiatry (Edgmont). 2007;4(7):28-37.

Cairns NJ, Bigio EH, Mackenzie IR, et al. Neuropathologic diagnostic and nosologic criteria for frontotemporal lobar degeneration: consensus of the Consortium for Frontotemporal Lobar Degeneration. Acta Neuropathol. 2007;114 (1):5-22.

Carrasquillo MM, Nicholson AM, Finch N, et al. Genome-wide screen identifies rs646776 near sortilin as a regulator of progranulin levels in human plasma. Am J Hum Genet. 2010;87(6):890-7.

Chang MC, Srinivasan K, Friedman BA, et al. Progranulin deficiency causes impairment of autophagy and TDP-43 accumulation. J Exp Med. 2017;214(9):2611-28.

Chitramuthu BP, Bennett HPJ, Bateman A. Progranulin: a new avenue towards the understanding and treatment of neurodegenerative disease. Brain. 2017;140(12):3081-104.

Cruts M, Gijselinck I, van der Zee J, et al. Null mutations in progranulin cause ubiquitin-positive frontotemporal dementia linked to chromosome 17q21. Nature. 2006;442(7105):920-4.

Department of Health and Human Services (DHHS), Food and Drug Administration (FDA), Center for Drug Evaluation and Research (US). Draft guidance. Guidance for Industry. Suicidal ideation and behavior: prospective assessment of occurrence in clinical trials. August 2012 [Cited 27 June 2019] [16 screens]. Available from: https://www.fda.gov/media/79482/download.

Evers BM, Rodriguez-Navas C, Tesla RJ, et al. Lipidomic and transcriptomic basis of lysosomal dysfunction in progranulin deficiency. Cell Rep. 2017;20(11):2565-74.

Galimberti D, Fumagalli GG, Fenoglio C, et al. Progranulin plasma levels predict the presence of GRN mutations in asymptomatic subjects and do not correlate with brain atrophy: results from the GENFI study. Neurobiol Aging. 2018:245.e9-245.

Gass J, Cannon A, Mackenzie IR, et al. Mutations in progranulin are a major cause of ubiquitin-positive frontotemporal lobar degeneration. Hum Mol Genet. 2006;15(20):2988-3001.

Ghidoni R, Paterlini A, Benussi L. Circulating progranulin as a biomarker for neurodegenerative diseases. Am J Neurodegener Dis. 2012;1(2):180-90.

Gollan TH, Weissberger GH, Runnqvist E, et al. Self-ratings of spoken language dominance: A Multi-lingual Naming Test (MINT) and preliminary norms for young and aging Spanish-English bilinguals. Biling (Camb Engl). 2012;15(3):594-615.

Gorno-Tempini ML, Brambati SM, Ginex V, et al. The logopenic/phonological variant of primary progressive aphasia. Neurology. 2008;71(16):1227-34.

Gorno-Tempini ML, Hillis AE, Weintraub S, et al. Classification of primary progressive aphasia and its variants. Neurology. 2011;76(11):1006-14.

Gray SJ, Nagabhushan Kalburgi S, McCown TJ, et al. Global CNS gene delivery and evasion of anti-AAV-neutralizing antibodies by intrathecal AAV administration in non-human primates. Gene Ther. 2013;20(4):450-9.

Guven G, Bilgic B, Tufekcioglu Z, et al. Peripheral GRN mRNA and serum progranulin levels as a potential indicator for both the presence of splice site mutations and individuals at risk for frontotemporal dementia. J Alzheimers Dis. 2019;67(1):159-67.

Hakim AM, Mathieson G. Dementia in Parkinson disease: a neuropathologic study. Neurology. 1979;29(9 Pt 1):1209-14.

He Z, Ismail A, Kriazhev L, et al. Progranulin (PC-cell-derived growth factor/acrogranin) regulates invasion and cell survival. Cancer Res. 2002;62(19):5590-6.

Hinderer C, Bell P, Vite CH, et al. Widespread gene transfer in the central nervous system of cynomolgus macaques following delivery of AAV9 into the cisterna magna. Mol Ther Methods Clin Dev. 2014;1:14051.

Hinderer C, Bell P, Katz N, et al. Evaluation of intrathecal routes of administration for adeno-associated viral vectors in large animals. Hum Gene Ther. 2018;29(1):15-24.

Hodges JR, Davies R, Xuereb J. et al. Survival in frontotemporal dementia. Neurology. 2003;61(3):349-54.

Hordeaux J, Hinderer C, Goode T, et al. Toxicology study of intra-cisterna magna adeno-associated virus 9 expressing human alpha-l-iduronidase in rhesus macaques. Mol Ther Methods Clin Dev. 2018;10:79-88.

Johnson JK, Diehl J, Mendez MF, et al. Frontotemporal lobar degeneration: demographic characteristics of 353 patients. Arch Neurol. 2005;62(6):925-30.

Kao AW, McKay A, Singh PP, et al. Progranulin, lysosomal regulation and neurodegenerative disease. Nat Rev Neurosci. 2017;18(6):326-33.

Kertesz A, Davidson W, Munoz DG. Clinical and pathological overlap between frontotemporal dementia, primary progressive aphasia and corticobasal degeneration: the pick complex. Dement Geriatr Cogn Disord. 1999;10(suppl 1):46-9.

Kertesz A, McMonagle P, Blair M, et al. The evolution and pathology of frontotemporal dementia. Brain. 2005;128(Pt 9):1996-2005.

Knopman DS, Kramer JH, Boeve BF, et al. Development of methodology for conducting clinical trials in frontotemporal lobar degeneration. Brain. 2008;131(Pt 11):2957-68.

Knopman DS, Roberts RO. Estimating the number of persons with frontotemporal lobar degeneration in the US population. J Mol Neurosci. 2011;45(3):330-35.

Lui H, Zhang J, Makinson S, et al. Progranulin deficiency promotes circuit-specific synaptic pruning by microglia via complement activation. Cell. 2016;165(4):921-35.

Mackenzie IR, Rademakers R. The molecular genetics and neuropathology of frontotemporal lobar degeneration: recent developments. Neurogenetics. 2007;8(4):237-48.

Mackenzie IR, Neumann M. FET proteins in frontotemporal dementia and amyotrophic lateral sclerosis. Brain Res. 2012;1462:40-3.

Manns MP, Czaja AJ, Gorham JD, et al. Diagnosis and management of autoimmune hepatitis. Hepatology. 2010;51(6):2193-213.

Matsumura N, Mandai M, Miyanishi M, et al. Oncogenic property of acrogranin in human uterine leiomyosarcoma: direct evidence of genetic contribution in in vivo tumorigenesis. Clin Cancer Res. 2006;12(5):1402-11.

Mendell JR, Al-Zaidy S, Shell R, et al. Single-dose gene-replacement therapy for spinal muscular atrophy. N Engl J Med. 2017;377(18):1713-22.

Mendoza TR, Williams LA, Shi Q, et al. The Treatment-induced Neuropathy Assessment Scale (TNAS): a psychometric update following qualitative enrichment. J Patient Rep Outcomes. 2020;4(1):15. doi:10.1186/s41687-020-0180-8.

Meyer K, Ferraiuolo L, Schmelzer L, et al. Improving single injection CSF delivery of AAV9-mediated gene therapy for SMA: a dose-response study in mice and nonhuman primates. Mol Ther. 2015;23(3):477-87.

Mingozzi F, Hasbrouck NC, Basner-Tschakarjan E, et al. Modulation of tolerance to the transgene product in a nonhuman primate model of AVV-mediated gene transfer to liver. Blood. 2007;110(7):2334-2341.

Miyagawa T, Brushaber D, Syrjanen J, et al. Use of the CDR® plus NACC FTLD in mild FTLD: Data from the ARTFL/LEFFTDS consortium. Alzheimers Dement. 2019;S1552-5260(19)34084-1.

Miyagawa T, Brushaber D, Syrjanen J, et al. Utility of the global CDR plus NACC FTLD rating and development of scoring rules: data from the ARTFL/LEFFTDS Consortium. Alzheimers Dement. 2020;16(1):106-117.

Nasreddine ZS, Phillips NA, Bédirian V, et al. The Montreal Cognitive Assessment, MoCA: a brief screening tool for mild cognitive impairment. J Am Geriatr Soc. 2005;53(4):695-9.

Neary D, Snowden JS, Gustafson L, et al. Frontotemporal lobar degeneration: a consensus on clinical diagnostic criteria. Neurology. 1998;51(6):1546-54.

Nevoret LM, Cho Y, Escolar ML, et al. Interim data from the first human RGX-121 gene therapy trial for the treatment of severe MPS II (Hunter Syndrome) [abstract]. Mol Ther. 2020;28(4S1):271. Abstract no. 641.

Nunnemann S, Last D, Schuster T, et al. Survival in a German population with frontotemporal lobar degeneration. Neuroepidemiology. 2011;37(3-4):160-5.

Ohno K, Samaranch L, Hadaczek P, et al. Kinetics and MR-based monitoring of AAV9 vector delivery into cerebrospinal fluid of nonhuman primates. Mol Ther Methods Clin Dev. 2018;13:47-54.

Onyike CU, Diehl-Schmid J. The epidemiology of frontotemporal dementia. Int Rev Psychiatry. 2013;25(2):130-7.

Pidala J, Hamadani M, Dawson P, et al. Randomized multicenter trial of sirolimus vs prednisone as initial therapy for standard-risk acute GVHD: The BMT CTN 1501 trial. Blood. 2020;135(2):97-107.

Posner K, Brent D, Lucas C, et al. Columbia-suicide severity rating scale (C-SSRS). Menands (NY): The Research Foundation for Mental Hygiene, Inc, 2008 (Recent version 23 June 2010) [Cited 23 July 2019] [3 screens]. Available from: http://cssrs.columbia.edu/wp-content/uploads/C-SSRS_Pediatric-SLC_11.14.16.pdf.

Ramsingh AI, Gray SJ, Reilly A, et al. Sustained AAV9-mediated expression of a non-self protein in the CNS of non-human primates after immunomodulation. PLoS ONE. 2018;13(6):e0198154.

Samaranch L, Salegio EA, San Sebastian W, et al. Adeno-associated virus serotype 9 transduction in the central nervous system of nonhuman primates. Hum Gene Ther. 2012;23(4):382-89.

Samulski JR, Muzyczka N. AAV-mediated gene therapy for research and therapeutic purposes. Ann Rev Virol. 2014;1(1):427-51.

Serrero G. Autocrine growth factor revisited: PC-cell-derived growth factor (progranulin), a critical player in breast cancer tumorigenesis. Biochem Biophys Res Commun. 2003;308(3):409-13.

Sha SJ, Miller ZA, Min SW, et al. An 8-week, open-label, dose-finding study of nimodipine for the treatment of progranulin insufficiency from GRN gene mutations. Alzheimers Dement (NY). 2017;3(4):507-12.

Sorrentino NC, Maffia V, Strollo S, et al. A comprehensive map of CNS transduction by eight recombinant adeno-associated virus serotypes upon cerebrospinal fluid administration in pigs. Mol Ther. 2016;24(2):276-86.

Tang W, Lu Y, Tian QY, et al. The growth factor progranulin binds to TNF receptors and is therapeutic against inflammatory arthritis in mice. Science. 2011;332(6028):478-84.

Tao J, Ji F, Wang F, et al. Neuroprotective effects of progranulin in ischemic mice. Brain Res. 2012;1436:130-6.

Teichmann M, Kas A, Boutet C, et al. Deciphering logopenic primary progressive aphasia: a clinical, imaging and biomarker investigation. Brain. 2013;136(Pt 11):3474-88.

Tolnay M, Probst A. Frontotemporal lobar degeneration - tau as a pied piper? Neurogenetics. 2002;4(2):63-75.

Valdez C, Wong YC, Schwanke M, et al. Progranulin-mediated deficiency of cathepsin D results in FTD and NCL-like phenotypes in neurons derived from FTD patients. Hum Mol Genet. 2017;26(24):4861-72.

Ward ME, Chen R, Huang HY, et al. Individuals with progranulin haploinsufficiency exhibit features of neuronal ceroid lipofuscinosis. Sci Transl Med. 2017;9(385):eaah5642.

Wang I-F, Guo B-S, Liu Y-C, et al. Autophagy activators rescue and alleviate pathogenesis of a mouse model with proteinopathies of the TAR DNA-binding protein 43. Proc Natl Acad Sci USA. 2012;109(37):15024-29.

Weinberg MS, Samulski JR, McCown TJ. Adeno-associated virus (AAV) gene therapy for neurological disease. Neuropharmacology. 2013;69:82-8.

Young JJ, Lavakumar M, Tampi D, et al. Frontotemporal dementia: latest evidence and clinical implications. Ther Adv Psychopharmacol. 2018;8(1):33-48.

Zerah M, Piguet F, Colle MA, et al. Intracerebral gene therapy using AAVrh.10-hARSA recombinant vector to treat patients with early-onset forms of metachromatic leukodystrophy: preclinical feasibility and safety assessments in nonhuman primates. Hum Gen Ther Clin Dev. 2015;26(2):113-24.

Zhou X, Paushter DH, Feng T, et al. Regulation of cathepsin D activity by the FTLD protein progranulin. Acta Neuropathol. 2017;134(1):151-3.

13 Appendices

13.1 Appendix: Schedule of Events

Table 13-1 Schedule of Events – Main Study Period

Visit Description	Screening/Baseline[a,b]			Treatment										Unscheduled
	Day							Month						
	-35 to -2	Check-In -1[c]	0[d]	1[e]	7	14	21	1	2	3	6	9	12/ ET[f]	
**Visit Window in Days**					±1	±2	±2	±7	+ 14	±7[g]				
**Administrative Procedures**														
Informed consent[h]	X													
Study partner consent[i]	X													
Issue patient identification card		X												
Inclusion/exclusion criteria	X	X												
Medical and family history and demographics	X													
Inpatient stay		X[c]	X[d]	X[e]										
**Clinical Assessments and Procedures**														
Height, weight, BMI, and waist circumference[j]	X								X	X	X	X	X	X
Physical examination[k]	X		X			X			X	X	X		X	X
Vital signs[l]	X	X	X	X	X	X	X	X	X	X	X	X	X	X
Prior and concomitant medication review	X	X	X	X	X	X	X	X	X	X	X	X	X	X
Adverse events	X[m]	X[m]	X	X	X	X	X	X	X	X	X	X	X	X
Adverse reactions	X	X	X	X	X	X		X	X	X	X	X	X	X
[redacted]														

**Visit Description**	**Screening/Baseline[a,b]**			**Treatment**										**Unscheduled**
	**Day**							**Month**						
	**-35 to -2**	**Check-In -1[c]**	**0[d]**	**1[e]**	**7**	**14**	**21**	**1**	**2**	**3**	**6**	**9**	**12/ ET[f]**	
**Visit Window in Days**					**±1**	**±2**	**±2**	**±7**	+ 14	**±7[g]**				
Brain MRA[no]	X													
DEXA scan[p]	X												X	
12-lead electrocardiogram[q]	X								X	X	X	X	X	X
Neurological examination[k]	X							X	X	X	X		X	X
[redacted]	[redacted]										[redacted]		[redacted]	[redacted]
MoCA	X								X	X	X	X	X	X
Benson Complex Figure	X										X		X	X
Category Fluency	X										X		X	X
CGI-S	X													
CGI-I											X		X	X
MINT	X										X		X	X
TMT-A, TMT-B	X										X		X	X
Digit Span Test (forwards and backwards)	X										X		X	X
C-SSRS (baseline)	X													
C-SSRS (since last visit)								X	X	X	X	X	X	X
TNAS	X							X	X	X	X		X	X

Visit Description	Screening/Baseline[a,b]			Treatment										Unscheduled
	Day							Month						
	-35 to -2	Check-In -1[c]	0[d]	1[e]	7	14	21	1	2	3	6	9	12/ ET[f]	
**Visit Window in Days**					±1	±2	±2	±7	+ 14	±7[g]				
**Laboratory Assessments**														
HBsAg, HCVAb, and HIV	X													
Hematology, blood chemistry (incl lipid panel and coagulation[r])	X	X			X	X	X	X	X	X	X	X	X	X
HbA1c[r,s]	X	X							X	X				X
Fructosamine[r]		X			X									X
Urinalysis	X	X			X	X	X	X	X	X	X	X	X	X
Urine biomarker	X							X	X	X	X	X	X	X
Pregnancy test[t]	X	X	X						X		X	X	X	
FSH (post-menopausal women only)[u]	X													
TSH and T4	X												X	X
Urine drug screen	X	X												
MTB test[v]	X													
Blood AAV9 and PGRN ELISpot	X				X	X		X	X	X	X	X	X	X
Blood sample for anti-AAV9 and anti-PGRN antibodies	X				X	X		X	X	X	X	X	X	X
Blood sample for full *GRN* sequencing[a]	X													

Visit Description	Screening/Baseline[a,b]			Treatment										Unscheduled
	Day							Month						
	-35 to -2	Check-In -1[c]	0[d]	1[e]	7	14	21	1	2	3	6	9	12/ ET[f]	
**Visit Window in Days**					±1	±2	±2	±7	+ 14	±7[g]				
Blood and urine sample(s) for future biomedical research (optional)[w]	X								X				X	
Lumbar puncture and CSF collection, including optional sample for future biomedical research[x]	X								X				X	X
Blood sample for biomarkers[y]	X							X	X	X	X	X	X	X
[redacted]														
Sirolimus trough levels					X	X	X	X	X	X				X
**Investigational Product Procedures**														
Pre-cisternal puncture anesthesiology check			X											
CSF from ICM collection[z]			X											
Investigational product administration[d]			X											
IV corticosteroid administration[c]			X											
Corticosteroid dispensation[c]				X										
Monitor compliance with immunosuppressive regimen (corticosteroids and/or sirolimus)					X	X	X	X	X	X				
Sirolimus dispensation[e]			X	X	X	X	X	X	X	X				

**Visit Description**	**Screening/Baseline[a,b]**			**Treatment**										**Unscheduled**
	**Day**							**Month**						
	**-35 to -2**	**Check-In -1[c]**	**0[d]**	**1[e]**	**7**	**14**	**21**	**1**	**2**	**3**	**6**	**9**	**12/ ET[f]**	
**Visit Window in Days**					±1	±2	±2	±7	+ 14	±7[g]				
Pneumococcal pneumonia vaccine[aa]	X													
Shingles vaccine[bb]	X													

Abbreviations: AAV9, adeno-associated virus serotype 9; AE, adverse event; BMI, body mass index; CDR plus NACC FTLD, Clinical Dementia Rating staging instrument plus National Alzheimer's Coordinating Center frontotemporal lobar degeneration domains; CGI-I, Clinical Global Impressions-Improvement; CGI-S, Clinical Global Impressions-Severity; CLIA, Clinical Laboratory Improvement Amendments; CSF, cerebrospinal fluid; C-SSRS, Columbia Suicide Severity Rating Scale; DEXA, dual-energy x-ray absorptiometry; ECG, electrocardiogram; ELISpot, enzyme-linked immunospot; FSH, follicle-stimulating hormone; GFAP, glial fibrillary acidic protein; *GRN*, progranulin gene; HbA1c, hemoglobin A1c; HbsAg, hepatitis B surface antigen; HCVAb, hepatitis C virus antibody; HIV, human immunodeficiency virus; ICM, intracisternal magna; LP, lumbar puncture; MoCA, Montreal Cognitive Assessment; MRA, magnetic resonance angiography; MRI, magnetic resonance imaging; MTB, *Mycobacterium tuberculosis*; NfL, neurofilament light chain; PGRN, progranulin protein; SAE, serious adverse events; T4, thyroxine; TMT-A, Trail Making Test Part A; TMT-B, Trail Making Test Part B; TNAS Treatment-Induced Neuropathy Assessment Scale; TSH, thyrotropin.

a. At Screening, patients that meet all criteria will be tested for full GRN sequencing by the central laboratory. Documentation of prior testing demonstrating the presence of at least 1 pathogenic GRN mutation from a CLIA-approved laboratory or an ex-US CLIA-equivalent certified laboratory that is verified by the Investigator is allowed so the patient can continue screening without waiting for central laboratory results. However, central laboratory confirmation of GRN is required prior to investigational product administration. Results from the external laboratory should be filed in the patient's medical record.

b. The protocol allows for a 35-day Screening Period, which may be extended with prior Sponsor approval.

c. Patients will complete Day -1 study assessments and, after the collection of blood samples, will receive a methylprednisolone pulse of 1 g IV on Day 0 (sites will be allowed to administer the methylprednisolone pulse at Day -1 depending on site preference and timing of the procedure), then prednisone 30 mg for 14 days, which will then be tapered over the ensuing 7 days. At the Investigator's discretion, higher doses or a longer taper of corticosteroids may be used (Section 5.5.1.3). A single sirolimus loading dose of 6 mg will be administered on Day 1 (with a window of Day -3 to Day -1) followed by 2 mg/day from Day 0 to Day 90 to maintain a trough level of 4 ng/mL (range 2 to 8 ng/mL). The sirolimus dose will be tapered over the next 15 to 30 days. Depending on local site requirements, patients have the option to complete an overnight inpatient stay from Day -1 to Day 0.

d. Investigational product will be administered as a single dose into the cisterna magna via suboccipital injection by an interventional radiologist or neurosurgeon. NOTE: Prior to injection, a volume of intracisternal fluid equivalent to the PR006A dosing volume will be removed. Patients will remain under observation for 24 hours (overnight inpatient stay) after PR006A administration.

e. Patients will be discharged on Day 1 from the study site after completion of the visit assessments.

f. If a patient who received investigational product withdraws prematurely from the study for any reason, study site personnel should make every effort to complete the full panel of assessments scheduled for the end of treatment visit. Patients who withdraw during the treatment period will complete the Month 12 (Year 1) assessments, while patients who withdraw during the follow-up period will complete the Month 60 assessments (Section 4.2.2). If the termination visit is more than 3 months prior to the scheduled Month 12 assessment, or more than 6 months prior to the Month 60 assessment for subjects who discontinue participation after Month 12, in addition to performing termination visit assessments, the investigator and study site personnel should make every effort to complete the Month 12 or Month 60 assessments, respectively, at the originally scheduled time points.

g. Visit window can be extended to +/- 14 days with prior Sponsor approval. All results from the prior visits must be at the site prior to the next visit.

h. Patients willing to provide blood and CSF samples for future biomedical research will be required to sign an optional additional section of the main ICF.

i. Consent for the patient and the study partner are not required to be obtained on the same day; however, consent must be obtained from each patient and their study partner before entering the study.

j. Height will be measured at Screening only. Waist circumference measurement should be made at the approximate midpoint between the lower margin of the last palpable rib and the top of the iliac crest. The tape should be snug around the body, but not pulled so tight that it is constricting. The mean of 2 measurements will be recorded.

k. Full physical and neurological examinations will be performed at Screening and Month 2. Brief symptom-based physical examinations and neurological examinations will be performed at all other time points, unless a full physical examination and/or neurological examination is deemed necessary by the Investigator.

l. Vital signs will be taken after a 5-minute seated rest and will include orthostatic blood pressure, pulse rate, respiratory rate, and oral or axillary body temperature. Vital sign measurements should be performed prior to any blood draws, whenever possible.

m. After signed written informed consent is provided but prior to ICM administration of investigational product, only AEs and SAEs determined to be related to a protocol-mandated intervention will be reported. All other AEs and SAEs during this time period will be recorded as medical history. After the ICM administration of investigational product, all AEs and SAEs will be reported.

n. Screening MRIs will be read locally and centrally; MRAs will only be read locally. Post-baseline brain MRIs for safety evaluation will be performed without contrast unless specifically requested by the Investigator.

o. At the Month 1 visit, only a safety brain MRI will be performed.

p. DEXA scan of the hip and spine should be performed only in patients not meeting osteoporosis exclusion criteria but who have a known medical history of osteoporosis or osteopenia or with multiple risk factors for osteoporosis.

q. The 12-lead ECGs will be performed after a 10-minute supine rest and will include heart rate, QRS, and QT interval and will be read locally. The Screening 12-lead ECG should be performed in triplicate. The 12-lead ECGs should be performed prior to any blood draws, whenever possible.

r. During Screening and until the end of the corticosteroid taper, blood chemistry will be performed fasting.

s. For patients receiving prednisone for more than 3 months, HbA1c will be measured at the end of prednisone therapy.

t. A serum pregnancy test will be performed at Screening and for any positive urine pregnancy tests. Urine pregnancy tests will be performed at Day -1 or Day 0 (before investigational product administration), and every 3 months for the first 12 months, for women of childbearing potential.

u. An FSH test will be performed at Screening for women who are post-menopausal with spontaneous amenorrhea for at least 2 years.

v. A negative Screening test for MTB or documented negative MTB test is required within 1 year prior to Screening.

w. Whole blood (for DNA, RNA, serum, plasma, and/or peripheral blood mononuclear cells) will be collected from patients who have consented to provide optional samples for future biomedical research.

x. [redacted]

y. [redacted]

z. [redacted].

aa. Patients must have evidence of a pneumococcal pneumonia vaccination coverage at Screening, and patients not previously vaccinated should receive pneumococcal vaccine administration at least 4 weeks prior to sirolimus loading dose.

bb. Patients must have evidence of shingles vaccination coverage at Screening and patients not previously vaccinated should receive shingles vaccine at least 4 weeks prior to sirolimus loading dose.

Table 13-2 Schedule of Events – Follow-Up Period

Visit Description	Follow-Up							
	Month							
	18	24	30	36	42	48	54	60/ Unsched-uled/ ET[a]
**Visit Window in Days**	±14 days; may be extended with prior sponsor approval							
**Clinical Assessments and Procedures**								
Physical examination[b]	X	X	X	X	X	X	X	X
Vital signs[c]	X	X	X	X	X	X	X	X
Prior and concomitant medication review	X	X	X	X	X	X	X	X
Adverse events	X	X	X	X	X	X	X	X
12-lead electrocardiogram[d]	X	X	X	X	X	X	X	X
Neurological examination[b]	X	X	X	X	X	X	X	X
[redacted]								
MoCA	X	X	X	X	X	X	X	X
Benson Complex Figure	X	X	X	X	X	X	X	X
Category Fluency	X	X	X	X	X	X	X	X
CGI-S	X	X	X	X	X	X	X	X
CGI-I	X	X	X	X	X	X	X	X
MINT	X	X	X	X	X	X	X	X
TMT-A, TMT-B	X	X	X	X	X	X	X	X
Digit Span Test (forwards and backwards)	X	X	X	X	X	X	X	X
C-SSRS (since last visit)	X	X	X	X	X	X	X	X
TNAS	X	X	X	X	X	X	X	X

**Visit Description**	**Follow-Up**							
	**Month**							
	**18**	**24**	**30**	**36**	**42**	**48**	**54**	**60/ Unsched -uled/ ET[a]**
**Visit Window in Days**	**±14 days; may be extended with prior sponsor approval**							
[redacted]								
**Laboratory Assessments**								
Blood sample for anti-AAV9 antibodies[f]	X	X						
Lumbar puncture and CSF collection (optional)[g]		X		X		X		X
Blood sample for biomarkers[h]	X	X	X	X	X	X	X	X
Hematology	X	X	X	X	X	X	X	X
Blood chemistry	X	X	X	X	X	X	X	X
Coagulation	X	X	X	X	X	X	X	X
Lipid panel	X	X	X	X	X	X	X	X
Urinalysis	X	X	X	X	X	X	X	X
Urine biomarker	X	X	X	X	X	X	X	X
Blood sample(s) for future biomedical research (optional)[i]	X	X	X	X	X	X	X	X

Abbreviations: AAV9, adeno-associated virus serotype 9; CDR plus NACC FTLD, Clinical Dementia Rating staging instrument plus National Alzheimer's Coordinating Center frontotemporal lobar degeneration domains; CGI-I, Clinical Global Impressions-Improvement; CGI-S, Clinical Global Impressions-Severity; CSF, cerebrospinal fluid; C-SSRS, Columbia Suicide Severity Rating Scale; MINT, Multilingual Naming Test; MoCA, Montreal Cognitive Assessment; PGRN, progranulin protein; TMT-A, Trail Making Test Part A; TMT-B, Trail Making Test Part B; TNAS, Treatment-Induced Neuropathy Assessment Scale.

Note: Follow-up visits may be performed by telephone or video conference if the patient is not available to return to the clinic. Safety assessments may be obtained remotely for review by the Investigator.

a. If a patient who received investigational product withdraws prematurely from the study for any reason, study site personnel should make every effort to complete the full panel of assessments scheduled for the end of treatment visit. Patients who withdraw during the treatment period will

complete the Month 12 (Year 1) assessments, while patients who withdraw during the follow-up period will complete the Month 60 assessments (Section 4.2.2). If the termination visit is more than 3 months prior to the scheduled Month 12 assessment, or more than 6 months prior to the Month 60 assessment for subjects who discontinue participation after Month 12, in addition to performing termination visit assessments, the investigator and study site personnel should make every effort to complete the Month 12 or Month 60 assessments, respectively, at the originally scheduled time points.

b. Brief symptom-based neurological examinations will be performed, unless a full neurological examination is deemed necessary by the Investigator.

c. Vital signs will be taken after a 5-minute seated rest and will include orthostatic blood pressure, pulse rate, respiratory rate, and oral or axillary body temperature. Vital sign measurements should be performed prior to any blood draws, whenever possible.

d. The 12-lead ECGs will be performed after a 10-minute supine rest and will include heart rate, QRS, and QT interval and will be read locally. The 12-lead ECGs should be performed prior to any blood draws, whenever possible.

e. Post-baseline brain MRIs for safety evaluation will be performed without contrast unless specifically requested by the Investigator.

f. Anti-AAV9 antibody testing will be performed only through Month 24.

g. [redacted]

h. [redacted]

i. Whole blood (for DNA, RNA, serum, plasma, and/or peripheral blood mononuclear cells) will be collected from patients who have consented to provide optional samples for future biomedical research.

13.2 Appendix: Recommended Sequence of Study Assessments

During Screening, *GRN* genotyping and eligibility assessments as per patient's medical and FTD history should be performed prior to proceeding with additional Screening assessments. Patients that meet all criteria will be tested for full *GRN* sequencing by the central laboratory; however, patients with a documented prior testing demonstrating the presence of at least 1 pathogenic *GRN* mutation may proceed with Screening until central laboratory results are available (Section 6.5). Whenever possible, scales (CDR plus NACC FTLD, CGI-S/I, C-SSRS, MoCA, etc.) should be completed at each visit prior to clinical assessments (vital signs, ECG, LP, urine and blood sampling). After the 1-month safety MRI, follow-up MRI scans may be done within a window of ±7 days from the clinic visit.
