# Supplementary material for: Progranulin AAV gene therapy for frontotemporal dementia: translational studies and phase 1/2 trial interim results
Source: Nat Med. 2024 May 14;30(5):1406–15. doi: 10.1038/s41591-024-02973-0 (PMC11108785; doi:10.1038/s41591-024-02973-0)
Supplement: Supplementary file 3 — Summary of changes to the clinical trial protocol. [file 41591_2024_2973_MOESM3_ESM.pdf]

Summary of Changes

Protocol Number: PRV-FTD101

A Phase 1/2 Ascending Dose Study to Evaluate the Safety and Effects on Progranulin Levels of PR006A in Patients with Fronto-Temporal Dementia with Progranulin Mutations (FTD-GRN)

Version of Protocol: 4.0

Date of Protocol: 15 July 2020

Date of Summary of Changes: 15 July 2020

CONFIDENTIAL

All financial and nonfinancial support for this study will be provided by Prevail Therapeutics, Inc. The concepts and information contained in this document or generated during this study are considered proprietary and may not be disclosed in whole or in part without the expressed, written consent of Prevail Therapeutics, Inc.

This study will be conducted according to the International Council for Harmonisation harmonised tripartite guideline E6(R2): Good Clinical Practice.

Protocol Version 4.0: (15 July 2020): Summary of Changes

This amendment is considered to be substantial based on the criteria set forth in Article 10(a) of Directive 2001/20/EC of the European Parliament and the Council of the European Union.

Overall Rationale for the Amendment

- The protocol was amended to optimize the immunosuppression regimen by including sirolimus and decreasing in dose and duration of prophylactic prednisone course.
- Modify staggering strategy to account for independent data monitoring committee (iDMC), Investigators and Sponsor review of Month 2 cerebrospinal fluid (CSF) safety results. 3 Month CSF sample will instead be collected at 2 Months (+2 weeks).
- Amend risk section to reflect information on potential for CSF inflammatory changes with adeno-associated virus serotype 9 (AAV9) gene therapy products.

Substantial changes from Version 3.0 (22 April 2020) to Version 4.0 (15 July 2020) are summarized in the following table. Additional minor changes are not included in this summary.

Section Number and Name	Description of Change
Synopsis, Section 2 Study Objectives and Endpoints, Section 7.1.1 Primary Safety Endpoints, Section 7.2.1 Secondary Efficacy Endpoints, Section 7.3.1 Exploratory Efficacy and Safety Endpoints (Baseline to Year 1), Section 7.3.2 Exploratory Efficacy and Safety Endpoints (Post-Year 1), Section 7.4 Sample Size Calculations	• Updated to include immunogenicity as a primary objective.<br>• Revised biomarker exploratory objective/endpoints to include lysosomal function and added urine and blood.<br>• Added safety endpoints/assessments.<br>• Adjusted change from baseline timing of some endpoint assessments.

Section Number and Name	Description of Change
Synopsis, Section 4.1.1 Inclusion Criteria, and Section 4.1.2 Exclusion Criteria	• Revised Inclusion Criterion 11 to include bilateral tubal ligation.<br>• Revised Inclusion Criterion 12 list of highly effective methods of contraception.<br>• Added Inclusion Criterion 18.<br>• Revised Exclusion Criterion 3 to include sirolimus and added uncontrolled hyperlipidemia or hypercholesterolemia, uncontrolled interstitial lung disease, and renal insufficiency.<br>• Revised Exclusion Criterion 5b to indicate that unstable autoimmune disease requiring chronic immunosuppression is exclusionary.<br>• Revised Exclusion Criterion 5n to include central nervous system (CNS) infections and revised the time frame to 12 weeks.<br>• Revised Exclusion Criterion 5o to add protocol-required immunosuppressant medications.<br>• Added Exclusion Criterion 5r.<br>• Revised Exclusion Criterion 6a to include a note about patients with Gilbert syndrome.<br>• Revised Exclusion Criterion 6d to remove chronic benign neutropenia as an exception.<br>• Revised Exclusion Criterion 6f to include other coagulopathy.<br>• Removed note from Exclusion Criterion 6i regarding successfully treated hepatitis C virus patients.<br>• Revised Exclusion Criterion 9 to allow shingles vaccine during the Screening Period.<br>• Revised Exclusion Criterion 13 to remove benzodiazepines.

Section Number and Name	Description of Change
Synopsis, Section 1.2.3 Rationale for Staggering of Patient Enrollment, Section 3.1.1 Year 1, Section 11.1.1 Independent Data Monitoring Committee	• Revised to indicate enrollment will be staggered by at least 8 weeks between the first 2 patients and then an additional 8 weeks between the second patient and the remaining 3 patients.<br>• Revised to indicate that the iDMC review will occur after at least 8 weeks after the second patient is dosed in Cohort 1.
Synopsis, Section 1.3 Risk Benefit, Section 2 Study Objective and Endpoints, Section 5.5.1.3 Immunosuppression Monitoring Criteria, Section 6.2.2.4 Risks Associated with AAV9-Based Therapy, Section 6.18 Treatment-induced Peripheral Neuropathy Scale, Section 7.1.1 Primary Safety Endpoints, Appendix 1 Section 13.1	• Replaced Rasch-Transformed Total Neuropathy Score clinical version (RT-TNSc) with Treatment-Induced Neuropathy Assessment Scale (TNAS).
Synopsis, Section 3.1.1 Year 1, Section 11.1.1 Independent Data Monitoring Committee	• Added text regarding enrollment at a lower dose level cohort.
Synopsis, Section 1.2.5 Rationale for Dosing, Section 3.1.1 Year 1, Section 5.5.1 Immunosuppressant Administration, Section 6.2.2.2 Risks Associated with the Use of Immunosuppressants	• Added administration of sirolimus and methylprednisone.<br>• Revised dosage and days of prednisone.
Section 1.2.3 Rationale for Staggering of Patient Enrollment	• Added additional justification for the 8-week monitoring period.<br>• Added text regarding enrollment termination based on biomarker review.
Section 1.2.5 Rationale for Dosing	• Added rationale for immunosuppressant regimen.

Section Number and Name	Description of Change
Section 1.3 Risk/Benefit	• Added information on safety and tolerability of products using AAV9.<br>• Added risk/benefit information on steroid-sparing immunosuppressive regimen consisting of corticosteroids and sirolimus.
Section 3.1.1 Year 1,<br>Section 11.1.1 Independent Data Monitoring Committee	• Removed list of safety assessments reviewed by the iDMC and replaced with a cross-reference to Section 2.
Section 4.1 Selection of Study Population	• Added text regarding determination of repeat screening assessments.
Section 5.5.1.3 Immunosuppression Monitoring Criteria	• Added information on monitoring sirolimus trough levels and monitoring for clinical signs or symptoms consistent with an immune response.
Section 5.5.6 Overdose Management	• Added section.
Section 5.5.7 Product Quality Complaint	• Added section.
Section 5.7.1 Excluded Medications and/or Procedures, Section 6.2.2.2 Risks Associated with the Use of Immunosuppressants, Table 13-1 Schedule of Events – Main Study Period	• Added shingles vaccination requirements.
Section 5.7.1 Excluded Medications and/or Procedures	• Added information on following all routinely scheduled immunizations.<br>• Added exclusionary medications during the period of sirolimus administration.
Section 5.7.2 Restricted Medications and/or Procedures	• Added strong inducers and inhibitors of CYP3A4/P-gp, including grapefruit juice and over the counter medications at least 7 days prior to or at least 48 hours after rapamycin administration.

Section Number and Name	Description of Change
Section 6.1.1 CDR Plus NACC FTLD	• Added text indicating that a Clinical Dementia Rating staging instrument plus National Alzheimer's Coordinating Center frontotemporal lobar degeneration domains (CDR plus NACC FTLD) global score will be calculated.
Section 6.2 Pharmacokinetic Assessments, Section 7.7.3.1 Exploratory Efficacy Endpoints	• Removed section and removed mention of measures as pharmacokinetic.
Section 6.2.1.1 Definitions of Adverse Events	• Added prescheduled or elective procedure or routinely scheduled treatment to list of events that are not considered adverse events.
Section 6.2.1.2 Serious Adverse Event	• Clarified definition of a serious adverse event.
Section 6.2.1.7 Expedited Reporting Requirements	• Added section.
Section 6.2.1.8 Suspected Unexpected Serious Adverse Reactions	• Removed text regarding comparison of suspected unexpected serious adverse reaction (SUSAR).
Section 6.2.2.2 Risks Associated with Use of Immunosuppressants	• Updated adverse events potentially associated with use of systemic corticosteroids.<br>• Updated list of assessments to be monitored at study visits occurring during corticosteroid usage and after prednisone discontinuation.<br>• Updated list of treatment-emergent adverse events (TEAEs) which may be associated with an immunosuppressant regimen.
Section 6.2.2.4 Risks Associated with AAV9-Based Therapy	• Added information on safety and tolerability of products using AAV9.<br>• Added information on pleocytosis and increased protein in CSF.<br>• Added information on risk mitigation of potential capsid and transgene related immune reactions.<br>• Added information on CSF pleocytosis.

Section Number and Name	Description of Change
Section 6.5 Laboratory Analyses	• Added sirolimus trough concentrations.<br>• Added anti-progranulin protein (PGRN) antibody and PGRN- enzyme-linked immunospot (ELISpot) to immunogenicity assays.
Section 6.6 Antibody/Biomarker Evaluation in Blood, Section 6.7 Biomarker Evaluation in Urine	• Added biomarkers for evaluation in blood and in urine.
Section 6.7 Biomarker Evaluation in Urine	• Section added
Section 6.8 Cerebrospinal Fluid Evaluations	• Added anti-PGRN antibodies and lysosomal function.
Section 6.9 Sampling for Future Biomedical Research	• Added genetic parameters.
Section 6.12 Physical Examinations, Section 6.13 Height, Weight, Body Mass Index, and Waist Circumference, Section 6.14 Neurological Examinations, Section 6.15 Magnetic Resonance Imaging and Magnetic Resonance Angiography	• Clarified that new or worsened clinically significant abnormal findings will be recorded as adverse events (AEs)/serious adverse events (SAEs).
Section 7.6 Description of Subgroups to be Analyzed	• Added phenotypic presentation of symptoms.
Section 11.1.2 Monitoring of the Study	• Added reference to the monitoring plan.
Section 11.2.1	• Removed sentence referring to Emergency Action Plan.
Section 12 Reference List	• Added the following references: Bharucha-Goebel et al 2020; Mendoza et al 2020; Mingozzi et al 2007; Miyagawa et al 2020; Nevoret et al 2020; Pidala et al 2020; Ramsingh et al 2018, Wang et al 2012.<br>• Removed the following references: Binda et al 2020; Cornblath et al 1999.

Section Number and Name	Description of Change
Section 13.1 Appendix: Schedule of Events; Table 13-1 Schedule of Events – Main Study Period	• Added visit on Day 21.<br>• Adjusted visit windows.<br>• Added assessments to Month 2.<br>• Added lipid panel to visits after Screening.<br>• Added PGRN.<br>• Added urine sample for future biomedical research.<br>• Added urine biomarkers.<br>• Added sirolimus trough levels.<br>• Added intravenous corticosteroid administration.<br>• Adjusted visit where corticosteroid is dispensed.<br>• Added shingles vaccine and accompanying footnote.<br>• Updated footnotes c, x, and z.<br>• Added footnote g regarding visit windows and adjusted lettering of subsequent footnotes.
Section 13.1 Appendix: Schedule of Events; Table 13-2 Schedule of Events – Follow-Up Period	• Added urine biomarkers.<br>• Updated footnote g.
Throughout	• Typographical, administrative, and/or formatting changes were also made to improve the clarity of the document.
